# Transcriptional profiling of *Zygosaccharomyces bailii* early response to acetic acid or copper stress mediated by ZbHaa1

**DOI:** 10.1038/s41598-018-32266-9

**Published:** 2018-09-20

**Authors:** Miguel Antunes, Margarida Palma, Isabel Sá-Correia

**Affiliations:** iBB-Institute for Bioengineering and Biosciences, Department of Bioengineering, Instituto Superior Técnico, Universidade de Lisboa, 1049-001 Lisbon, Portugal

## Abstract

The non-conventional yeast species *Zygosaccharomyces bailii* is remarkably tolerant to acetic acid, a highly important microbial inhibitory compound in Food Industry and Biotechnology. ZbHaa1 is the functional homologue of *S. cerevisiae* Haa1 and a bifunctional transcription factor able to modulate *Z. bailii* adaptive response to acetic acid and copper stress. In this study, RNA-Seq was used to investigate genomic transcription changes in *Z. bailii* during early response to sublethal concentrations of acetic acid (140 mM, pH 4.0) or copper (0.08 mM) and uncover the regulatory network activated by these stresses under ZbHaa1 control. Differentially expressed genes in response to acetic acid exposure (297) are mainly related with the tricarboxylic acid cycle, protein folding and stabilization and modulation of plasma membrane composition and cell wall architecture, 17 of which, directly or indirectly, ZbHaa1-dependent. Copper stress induced the differential expression of 190 genes mainly involved in the response to oxidative stress, 15 ZbHaa1-dependent. This study provides valuable mechanistic insights regarding *Z. bailii* adaptation to acetic acid or copper stress, as well as useful information on transcription regulatory networks in pre-whole genome duplication (WGD) (*Z. bailii*) and post-WGD (*S. cerevisiae*) yeast species, contributing to the understanding of transcriptional networks’ evolution in yeasts.

## Introduction

*Zygosaccharomyces bailii* is described as the most problematic food spoilage yeast due to its remarkable high tolerance to weak acids, namely acetic acid^1^. Compared to *Saccharomyces cerevisiae*, *Z. bailii* displays a three-fold higher tolerance to this acid^2^. This marked difference has brought much interest in uncovering the molecular mechanisms underlying *Z. bailii* tolerance to acetic acid stress, compared with the model yeast *S. cerevisiae* (a topic recently reviewed by Palma *et al*.^3^). A large part of what is currently known regarding the global molecular mechanisms underlying *S. cerevisiae* response and tolerance to sub-lethal or lethal concentrations of acetic acid comes from the consolidation and exploitation of diverse functional genomic approaches employed throughout the last two decades^3^. However, the utilization of this kind of approaches in a non-conventional yeast species such as *Z. bailii* is still scarce, partially due to the fact that only recently the annotated genome sequences of *Z. bailii* strains^4,5^ or *Z. bailii*-derived hybrid strains^6,7^ were released. This genomic data, not only provided new insights into the genetic and physiological traits of *Z. bailii sensu lato* clade, but also rendered available fundamental genomic information for the elucidation of tolerance mechanisms to acetic acid at a genome-wide scale^3^.

Different functional genomic-based approaches have been conducted to examine the biological processes involved in *Z. bailii* adaptation and tolerance to acetic and lactic acids, specifically, two-dimensional gel electrophoresis (2DE)-based expression proteomics^8,9^, metabolomics^10^, plasma membrane lipidomics^11^ and transcriptomics^7^. However, the genome-wide regulation of transcriptional alterations occurring in *Z. bailii* in response to acetic acid-induced stress is still unexplored. To date, only two transcription factors were demonstrated as being involved in *Z. bailii* tolerance to acetic acid, specifically, ZbMsn4^12^, the single homologue of *S. cerevisiae* Msn4 and Msn2 general stress response activators^13^, and ZbHaa1^14^, the homologue of *S. cerevisiae* transcription factor Haa1, the master regulator required for the direct or indirect activation of 80% of the acetic acid-responsive genes in *S. cerevisiae*^15–17^. Haa1 was first identified as a Cup2 (alias Ace1) paralogue based on sequence homology to the Cu-activated DNA binding domain and N-terminal Zn module^18^. However, contrarily to what the sequence homology to Cup2 would indicate, the function of Haa1 is not affected by the copper status of the cell^18^. Haa1-mediated transcriptional activation requires its interaction with the DNA binding sequence 5′-(G/C)(A/C)GG(G/C)G-3′, designated Haa1 responsive element (HRE), present in the promoter region of acetic-acid-responsive genes^17^. ZbHaa1 was found to be required for the adaptive response and tolerance to both acetic acid and copper stress by *Z. bailii*, activating the transcription of genes homologous to *S. cerevisiae* Haa1 and Cup2 targets, under acetic acid- or copper-induced stress, respectively^14^. Therefore, ZbHaa1 was proposed as a bifunctional transcription factor, assuming the functions of *S. cerevisiae* paralogues Haa1 and Cup2 originated after the whole genome duplication (WGD) event^14^.

The aim of the present study is to examine the alterations occurring in the transcriptome profile of *Z. bailii* IST302 cells during early response to acetic acid- or copper- induced stress mediated by ZbHaa1. The strain IST302 was used herein since its annotated genome was recently released, is haploid, and much more amenable to genetic manipulation and physiological studies than other studied strains^5^. This study allowed the identification of ZbHaa1-dependent regulons active during the early adaptive response to acetic acid- or copper- induced stress. It also provides useful information to allow the comparison of regulatory networks in a pre-WGD yeast species (*Z. bailii*) and a post-WGD species (*S. cerevisiae*) and to gain insights into the evolution of transcriptional networks in yeasts.

## Results

### Effect of acetic acid or copper stress in the growth of *Z. bailii* IST302 and derived mutant with the *ZbHAA1* gene deleted

**Figure 1:**
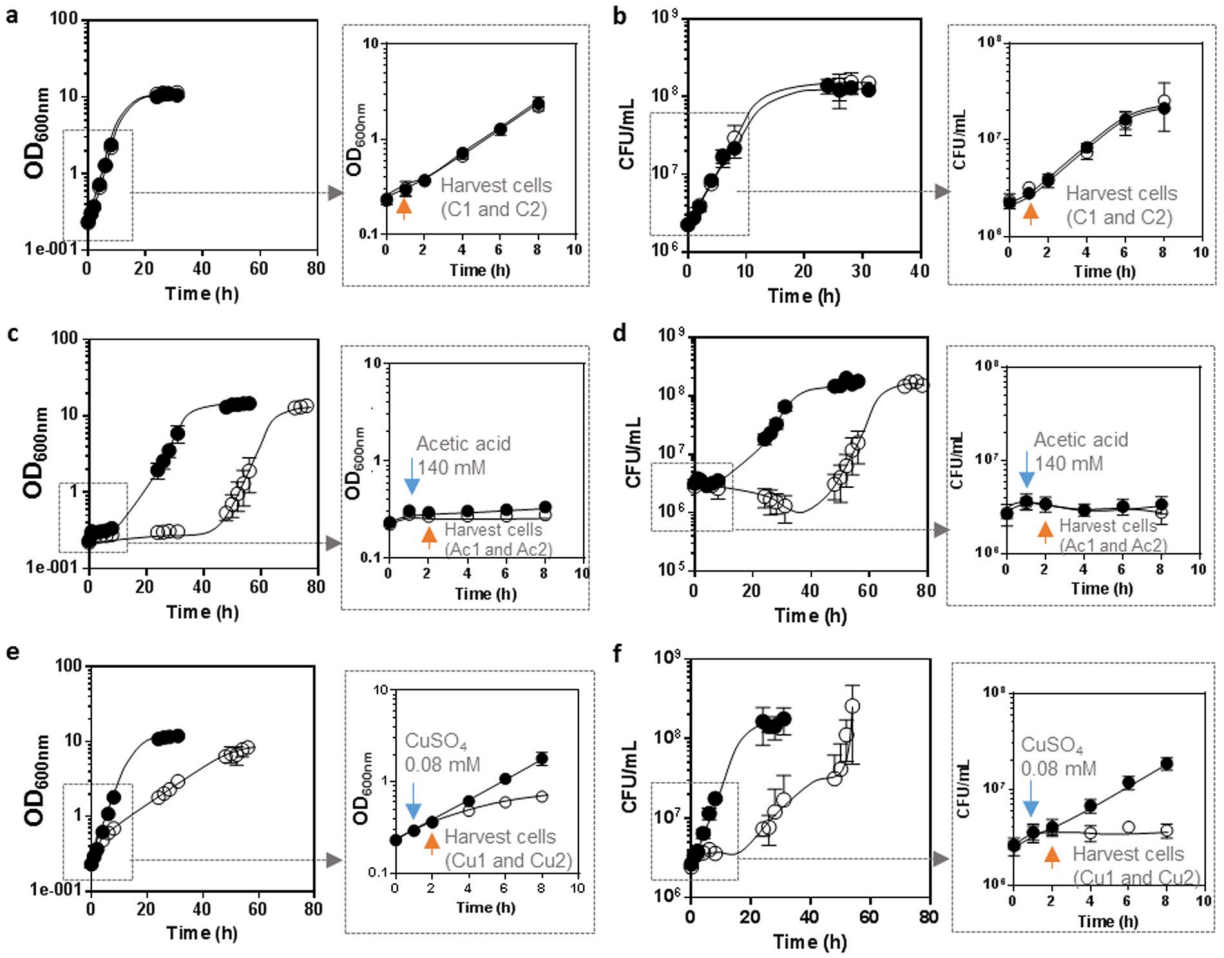
Growth curves of *Z*. *bailii* IST302 (●) and derived deletion mutant *Zbhaa1*∆ (○) based on culture optical density (OD 600 nm) (**a**,**c** and **e**) and on the viable cell concentration (CFU/mL) (**b**,**d** and **f**). Yeast cells were cultivated in MM medium at pH 4.0 (**a** and **b**) or in the same medium supplemented with acetic acid (**c** and **d**) or CuSO_4_ (**e** and **f**). Acetic acid or CuSO_4_ were added after 1 hour of cultivation of exponential cells grown under standardized conditions in MM medium to a final concentration of 140 mM or 0.08 mM, respectively. A detailed view of the first eight hours of cultivation is shown on the right of each graph. Cells were harvested for mRNA-Seq analysis before acetic acid or copper supplementation and 1 hour after acetic acid or copper addition.

In order to evaluate the genome-wide transcriptional changes occurring during early response of *Z. bailii* IST302 and of the derived deletion mutant *Zbhaa1***∆** to acetic acid (140 mM, pH 4.0) or CuSO_4_ (hereafter designated as copper) (0.08 mM, pH 4.0) stress, unadapted exponentially growing cells of the two strains were inoculated under standardized conditions (Fig. 1a,b). After 1 hour of cultivation, acetic acid (Fig. 1c,d) or copper (Fig. 1e,f) were added to the culture medium and the cells collected after 1 hour of exposure to the respective stress. The effects of these stressing conditions in the growth curves of the strains under study were characterized in detail.

The latency periods for the two strains examined when exposed to acetic acid were very distinct, with the parental strain displaying a latency period of about 10 hours while the mutant *Zbhaa1***∆** showed a latency period of approximately 40 hours (Fig. 1c,d). During this period of adaptation, while the concentration of viable cells did not change significantly immediately after acetic acid supplementation of the parental strain culture medium (Fig. 1d), the mutant *Zbhaa1***∆** cell population gradually lost viability until growth resumption after 40 hours of cultivation with acetic acid (Fig. 1d). The differences observed in the more rapid resumption of the exponential growth under acetic acid stress in the parental strain is an indication that, as previously described for Haa1 in *S. cerevisiae*^15^, ZbHaa1 plays an essential role during the period of adaptation to this stress.

Upon exposure to copper stress, in both the parental and derived mutant strains, no apparent latency phase was observed based on culture optical density (Fig. 1e), but the maximum specific growth rate of both strains decreased. Furthermore, based on the growth curves assessed by the concentration of viable cells, a latency period was identified for the mutant strain after copper supplementation, characterized by the maintenance of the concentration of viable cells (Fig. 1f). It should be noted the aggregation of cells from both strains upon copper exposure was registered by microscopic observation and this factor might have interfered with the assessment of culture optical density and colony forming units (Supplementary Fig. S1). When cultivated in either minimal or rich media, *Z. bailii* IST302 does not form cellular aggregates as it was previously reported^5^. Nevertheless, the difference in the growth curves of both strains is remarkable and an indication that ZbHaa1 also plays an essential role in adaptation and tolerance to copper stress, as observed for acetic acid-induced stress and as previously reported^14^.

### Transcriptional profiling of the early response of *Z. bailii* IST302 to acetic acid- or copper- induced stress

The analysis of the changes occurring in the transcriptome of the parental strain when exposed to acetic acid or copper stress, compared with the control condition, led to the identification of the differently expressed genes (DEGs), using as cut-off values a Fold change > 1.50 and an FDR < 0.05. The identified genes were submitted to a Gene Ontology (GO) term enrichment analysis by running a Fisher Exact Test with Blast2GO^19^.

For the concentration of acetic acid tested (140 mM at pH 4.0), 297 genes were found to exhibit significant changes in the transcription levels when compared to the unstressed condition. From these genes, 66 were found to have increased mRNA levels (presumably upregulated) while 231 genes were identified as exhibiting lower mRNA levels (presumably downregulated) than the unstressed cells (Supplementary Tables S1 and S2). Regarding the parental strain, during the early adaptive response to copper stress (0.08 mM), 190 genes were identified as producing different mRNA levels when compared to the control condition. Among these, 121 genes were found to be upregulated while 69 genes were downregulated (Supplementary Tables S3 and S4).

**Figure 2:**
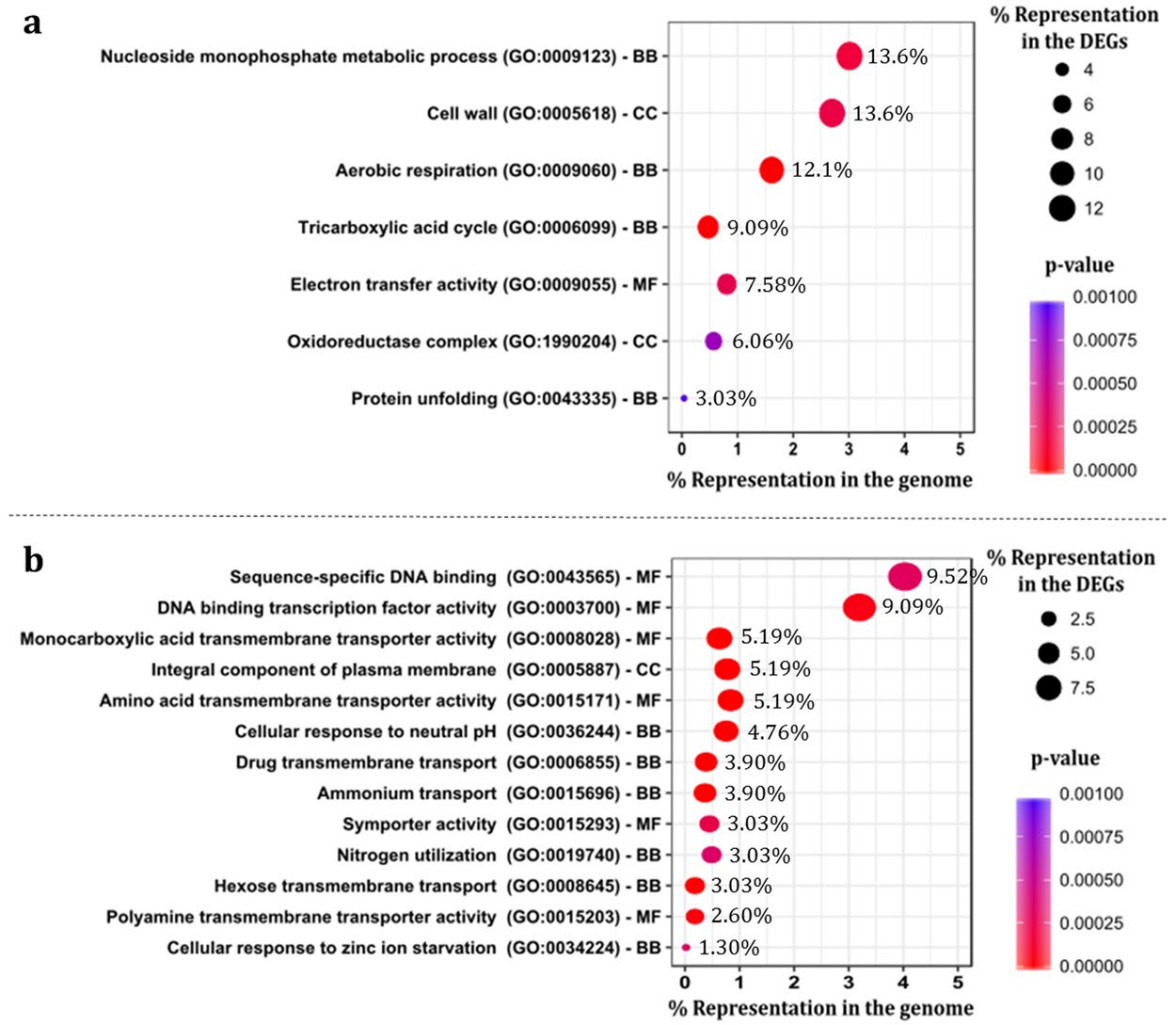
Enriched GO terms associated with the differently expressed genes in the early response to acetic acid stress. Dot plots displaying the percentage of differently expressed genes (DEGs) attributed to a GO term. (**a**) Upregulated genes in *Z. bailii* IST302 during exposure to acetic acid stress. (**b**) Downregulated genes in *Z. bailii* IST302 during exposure to acetic acid stress. The GO terms are sorted by molecular function (MF), cellular component (CC) and biological process (BP). The numeral percentages of DEGs assigned to each GO term were calculated based on the total number of upregulated (66) or downregulated (231) genes during exposure to acetic acid stress.

The most prominent enriched GO terms associated with the upregulated genes during *Z. bailii* exposure to acetic acid stress included the “Aerobic respiration” term, which includes genes related with the “Tricarboxylic acid cycle” (homologous to *S. cerevisiae ACO1*, *CIT1*, *LAT1*, *MDH1*, *SDH2* and *SHH3*); “Protein unfolding” (includes genes homologues of *S. cerevisiae HSP26*, *HSP42*, *HSP78*, *HSP104* and *SSA3*); and “Cell wall” (includes genes homologues of *S. cerevisiae ECM33* and *HSP150*) (Fig. 2a).

The GO term enrichment analysis regarding the downregulated genes during *Z. bailii* IST302 exposure to acetic acid stress revealed that the highest number of genes are associated with sequence-specific DNA binding, having several genes in common with the “DNA binding transcription factor activity” term, and including 15 transcription factors (homologous to *S. cerevisiae ADR1*, *CAT8*, *CST6*, *GAT1*, *GSM1*, *MET32*, *MIG2*, *NDT80*, *NRG2*, *ROX1*, *SFL1*, *SOK2*, *YRM1*, and *ZAP1*) (Fig. 2b). Other prominent enriched GO terms comprise plasma and mitochondrial membrane components, for example, the GO terms “Monocarboxylic acid transmembrane transporter activity” (includes homologues of *S. cerevisiae CRC1, MCH4* and *YHL008C*) and “Amino acid transmembrane transporter activity” (includes homologues of *S. cerevisiae BAP3*, *CAN1*, *GAP1* and *OPT1*).

**Figure 3:**
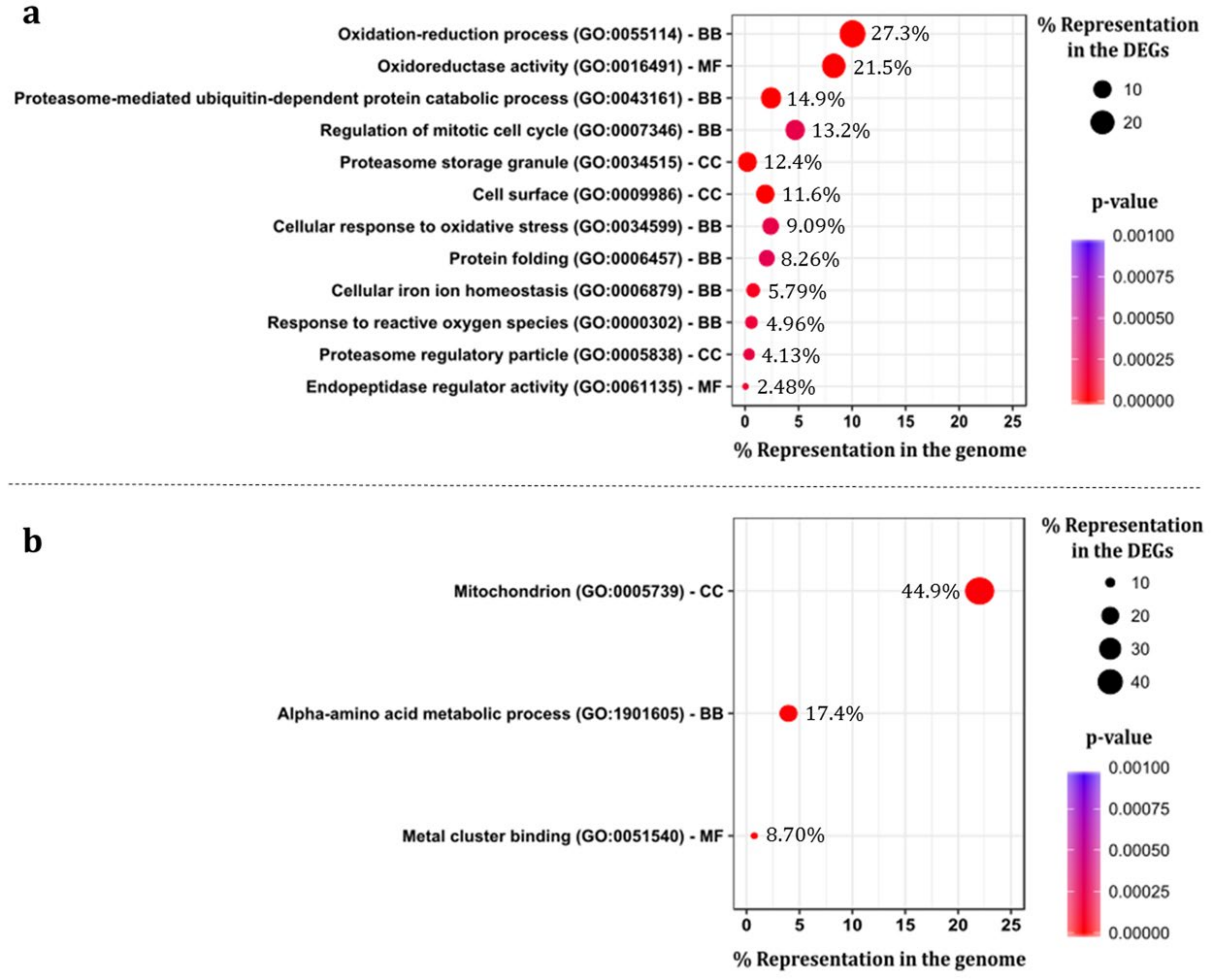
Enriched GO terms associated with the differently expressed genes in the early response to copper stress. Dot plots displaying the percentage of differently expressed genes (DEGs) attributed to a GO term. (**a**) Upregulated genes in *Z. bailii* IST302 during exposure to copper stress. (**b**) Downregulated genes in *Z. bailii* IST302 during exposure to copper stress. The GO terms are sorted by molecular function (MF), cellular component (CC) and biological process (BP). The numeral percentages of DEGs assigned to each GO were calculated based on the total number of upregulated (121) or downregulated (69) genes during exposure to copper stress.

The GO term associated with the highest number of genes found to be upregulated during *Z. bailii* IST302 exposure to copper stress is “Oxidation-reduction process”. Among the 33 genes related with this GO term, are the homologues of *S. cerevisiae* genes involved in processes such as the detoxification of oxygen radicals (*GRX2*, *HMX1, SOD1, TRX3* and *TSA1*), the transport of heavy metals (*FET3* and *FRE3*), and alcoholic fermentation enzymes (*ADH1* and *ADH3*). Furthermore, the GO term corresponding to “Proteasome-mediated ubiquitin-dependent protein catabolic process” was associated with 18 genes, most of them also associated with the “Regulation of mitotic cycle term”. Among these, genes homologous to *S. cerevisiae* genes coding for subunits of the 20 S proteasome (*PRE2–10*, *PUP1–3*, and *SCL1*) were identified which suggests degradation of proteins resulting from copper-induced oxidative damage (Fig. 3a).

The most prominent GO term associated with the downregulated genes identified from *Z. bailii* IST302 exposure to copper stress is “Mitochondrion”. This term is associated with 31 genes, including 8 genes also related to the metabolism of amino acids and genes encoding mitochondrial ribosomal proteins (homologous to *S. cerevisiae MRP13, MRPL23, RSM26, SWS2* and *YML6*) (Fig. 3b).

### Transcriptional profiling of the early response of the deletion mutant *Zbhaa1*∆ to acetic acid- or copper- induced stress

Sudden exposure of the *Zbhaa1***∆** deletion mutant to acetic acid stress, compared with unstressed cells, led to the identification of alterations in the transcription levels of 56 genes, one of which found to be upregulated while 55 were downregulated (Supplementary Table S5). Some of these genes have putative *S. cerevisiae* homologues previously described as having a role in acetic acid tolerance mechanisms and regulated by Haa1^16^. These include *HSP26*, *HSP30, HSP42, HSP78, HSP104, YGP1, HRK1 and YRO2*^16^. Under the selected conditions, exposure of the *Zbhaa1***∆** deletion mutant to copper stress led to the identification of changes in the mRNA levels from a much larger number of genes (1301), when compared with the unstressed cells. Among these, 663 were found to have increased mRNA levels, while 638 exhibited decreased mRNA levels (Supplementary Tables S6 and S7). The *CRS5* homologue is the sole gene from the DEGs whose homologue was previously described as being regulated by Cup2 upon copper stress^20^.

### Identification of the ZbHaa1-dependent genes transcriptionally activated upon acetic acid or copper exposure

**Figure 4:**
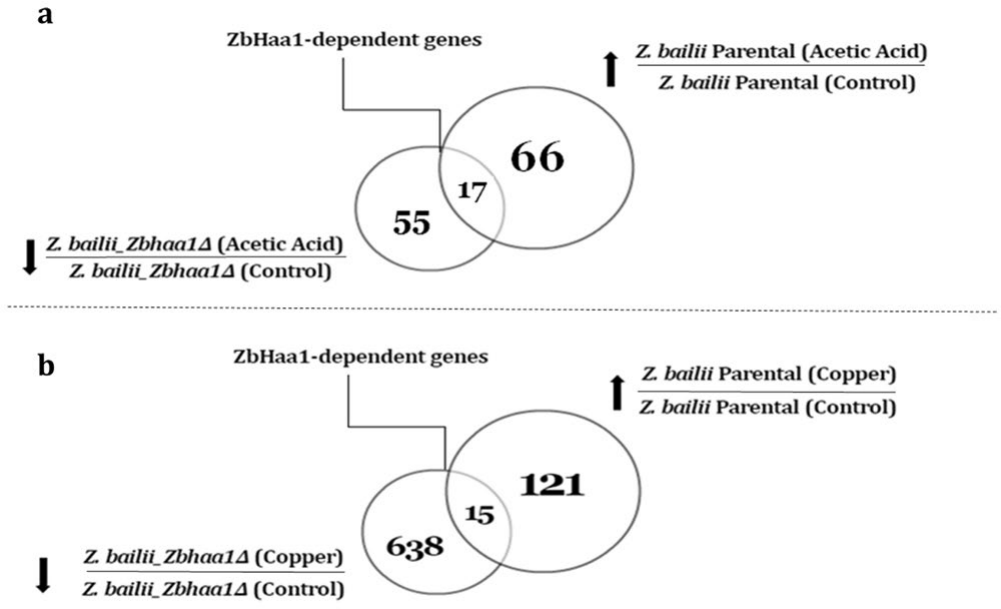
Number of ZbHaa1-dependent genes activated in *Z. bailii* IST302 during exposure to acetic acid or copper stress. (**a**) Venn diagram depicting the number of upregulated genes (up arrow) in the parental strain, downregulated genes (down arrow) in the deletion mutant *Zbhaa1***∆** during exposure to acetic acid stress, and the common differently expressed genes (DEGs) between them (ZbHaa1-dependent genes). (**b**) Venn diagram depicting the number of upregulated genes (up arrow) in the parental strain, downregulated genes (down arrow) in the deletion mutant *Zbhaa1***∆** during exposure to copper stress, and the common DEGs between them (ZbHaa1-dependent genes).

The next step in the dataset analysis was the identification of the genes whose regulation is dependent on the expression of the ZbHaa1 transcription regulator, homologue of *S. cerevisiae* Haa1 transcription activator. These were selected based on the upregulated genes in the parental strain upon exposure to each stress relative to the control condition and, among these, the genes that were downregulated in the *Zbhaa1***∆** deletion mutant upon stress exposure relative to the parental strain exposed to the same stress (Fig. 4). Genes fulfilling these conditions are listed in Tables 1 and 2 for acetic acid or copper stress exposure, respectively.

**Table 1:** ZbHaa1 regulon in *Z. bailii* IST302 response to acetic acid stress.

ORF	*S. cerevisiae* Homologue	$\frac{{\boldsymbol{(}}{\bf{mRNA}}\,{\bf{Wt}}{\boldsymbol{)}}{\bf{Control}}}{{\boldsymbol{(}}{\bf{mRNA}}\,{\bf{Wt}}{\boldsymbol{)}}{{\bf{AA}}}^{{\boldsymbol{(}}{\bf{1}}{\boldsymbol{)}}}}$	$\frac{{\boldsymbol{(}}{\bf{mRNA}}\,{\bf{Z}}{\bf{b}}{\bf{h}}{\bf{a}}{\bf{a}}{\bf{1}}{\boldsymbol{)}}{\bf{Control}}}{{\boldsymbol{(}}{\bf{mRNA}}\,{\bf{Z}}{\bf{b}}{\bf{h}}{\bf{a}}{\bf{a}}{\bf{1}}{\boldsymbol{)}}{{\bf{AA}}}^{{\boldsymbol{(}}{\bf{1}}{\boldsymbol{)}}}}$	Description
ZBIST_3442	*HSP26*	8.74	−3.21	Small heat shock protein (sHSP) with chaperone activity
ZBIST_0079	*HSP26*	3.33	−2.81	Small heat shock protein (sHSP) with chaperone activity
ZBIST_5024	N/A	1.69	−1.09	Related to mitochondrial outer membrane protein *OM45*
ZBIST_4572	N/A	1.24	−1.48	Related to altered inheritance of mitochondria protein 19 (*AIM19*), mitochondrial
ZBIST 3873	N/A	1.22	−1.47	Related to sorbose reductase homolog *SOU2*
ZBIST_2207	*SSA3*	1.22	−1.18	ATPase involved in protein folding and the response to stress
ZBIST_4116	*HSP42*	1.20	−1.16	Small heat shock protein (sHSP) with chaperone activity
ZBIST_1688	*HSP104*	1.18	−1.07	Disaggregase; heat shock protein that cooperates with Ydj1 (Hsp40) and Ssa1 (Hsp70) to refold and reactivate previously denatured, aggregated proteins
ZBIST_0509	*YGP1*	1.17	−2.38	Cell wall-related secretory glycoprotein; induced by nutrient deprivation-associated growth arrest and upon entry into stationary phase
ZBIST_0021	*MMF1*	1.07	−1.65	Mitochondrial protein required for transamination of isoleucine
ZBIST_5112	*ARR3*	0.96	−2.27	Plasma membrane metalloid/H^ +^ antiporter
ZBIST_3490	N/A	0.85	−1.13	Related to protein *FMP16*, mitochondrial
ZBIST_4760	*HSP78*	0.85	−0.80	Oligomeric mitochondrial matrix chaperone; cooperates with Ssc1 in mitochondrial thermotolerance after heat shock; able to prevent the aggregation of misfolded proteins as well as resolubilize protein aggregates
ZBIST_0039	N/A	0.83	−1.90	Uncharacterized protein
ZBIST_2306	*MCR1*	0.81	−0.70	Mitochondrial NADH-cytochrome b5 reductase; involved in ergosterol biosynthesis
ZBIST_5070	N/A	0.78	−2.10	Uncharacterized protein
ZBIST_4902	*MDH1*	0.78	−0.78	Mitochondrial malate dehydrogenase; catalyzes interconversion of malate and oxaloacetate; involved in the tricarboxylic acid (TCA) cycle; phosphorylated

^(1)^AA- Acetic Acid stress

ZbHaa1-dependent genes in *Z. bailii* IST302 response to acetic acid stress. Expression values are represented as log_2_-fold change. Gene function descriptions were taken from *Saccharomyces* Genome Database (SGD).

**Table 2:** ZbHaa1 regulon in *Z. bailii* IST302 response to copper stress.

ORF	*S. cerevisiae* Homologue	$\frac{{\boldsymbol{(}}{\bf{mRNA}}\,{\bf{Wt}}){\bf{Control}}}{{\boldsymbol{(}}{\bf{mRNA}}\,{\bf{Wt}}{\boldsymbol{)}}{{\bf{Cu}}}^{{\boldsymbol{(}}{\bf{1}}{\boldsymbol{)}}}}$	$\frac{{\boldsymbol{(}}{\bf{mRNA}}\,{\bf{Z}}{\bf{b}}{\bf{h}}{\bf{a}}{\bf{a}}{\bf{1}}{\boldsymbol{)}}{\bf{Control}}}{{\boldsymbol{(}}{\bf{mRNA}}\,{\bf{Z}}{\bf{b}}{\bf{h}}{\bf{a}}{\bf{a}}{\bf{1}}{\boldsymbol{)}}{{\bf{Cu}}}^{{\boldsymbol{(}}{\bf{1}}{\boldsymbol{)}}}}$	Description
ZBIST_1696	N/A	4.50	−3.09	Related to cation transport ATPase
ZBIST_3714	N/A	4.13	−2.36	Uncharacterized protein
ZBIST_3713	*CRS5*	2.80	−1.22	Copper-binding metallothionein; required for wild-type copper resistance
ZBIST_4639	*ZRT1*	2.67	−5.16	High-affinity zinc transporter of the plasma membrane; responsible for the majority of zinc uptake;
ZBIST_1985	*TMT1*	2.09	−1.11	Trans-aconitate methyltransferase; cytosolic enzyme that catalyzes the methyl esterification of 3-isopropylmalate
ZBIST_4824	N/A	1.19	−0.89	Uncharacterized protein
ZBIST_3873	N/A	1.07	−0.60	Related to sorbose reductase homolog SOU2
ZBIST_2268	*CLB5*	0.92	−1.10	B-type cyclin involved in DNA replication during S phase; activates Cdc28 to promote initiation of DNA synthesis;
ZBIST_1594	*GDH3*	0.83	−0.77	NADP(+)-dependent glutamate dehydrogenase; synthesizes glutamate from ammonia and alpha-ketoglutarate;
ZBIST_0903	*ADH3*	0.82	−1.55	Mitochondrial alcohol dehydrogenase isozyme III; involved in the shuttling of mitochondrial NADH to the cytosol under anaerobic conditions and ethanol production
ZBIST_2835	N/A	0.81	−1.36	Uncharacterized protein
ZBIST_4280	*GOR1*	0.81	−1.33	Glyoxylate reductase; null mutation results in increased biomass after diauxic shift;
ZBIST_4165	*FRE3*	0.75	−0.70	Ferric reductase; reduces siderophore-bound iron prior to uptake by transporters; expression induced by low iron levels
ZBIST_1042	*MDH2*	0.64	−1.50	Cytoplasmic malate dehydrogenase; one of three isozymes that catalyze interconversion of malate and oxaloacetate;
ZBIST_1788	*SFC1*	0.63	−0.65	Mitochondrial succinate-fumarate transporter; transports succinate into and fumarate out of the mitochondrion; required for ethanol and acetate utilization

^(1)^Cu- Copper stress

ZbHaa1-dependent genes in *Z. bailii* IST302 adaptation to copper stress. Expression values are represented as log_2_-fold change. Gene function descriptions were taken from *Saccharomyces* Genome Database (SGD).

#### Genes activated by ZbHaa1 under acetic acid stress

The elimination of *ZbHAA1* gene led to a reduction in the transcription levels from 17 out of the 66 genes that were activated in the early response to acetic acid exposure in the parental strain (Fig. 4, Supplementary Tables S1 and S5). This set of 17 genes are presumably regulated by the transcription factor ZbHaa1 either as direct or indirect targets (Table 1). Among these genes, three have sequence homology to *S. cerevisiae* genes reported to be directly activated by Haa1: *HSP26* (ORFs ZBIST_0079 and ZBIST_3442) and *YGP1* (ORF ZBIST_0509)^16^. Differently from *S. cerevisiae*, *Z. bailii* has four copies of *HSP26* homologues (ORFs ZBIST_0079, ZBIST_3334, ZBIST_3442 and ZBIST_4487). However, only the two aforementioned *HSP26* homologues (ORFs ZBIST_0079 and ZBIST_3442) were found to be transcriptionally activated and displayed the highest fold-change registered (Supplementary Table S1). The *Z. bailii YGP1* homologue, putatively encoding a cell-wall related secretory glycoprotein expressed in response to nutrient limitation^21^, may play a role in the remodeling of the cell wall structure and contribute to *Z. bailii* tolerance to acetic acid. This gene was shown to be activated by ZbHaa1 through Real Time Reverse Transcription-Polymerase Chain Reaction (RT-PCR) in a previous study, corroborating the obtained result^14^. Most of the genes considered here to be ZbHaa1-dependent in the early response of *Z. bailii* IST302 to sudden acetic acid exposure are homologous to genes encoding chaperones or co-chaperones involved in protein folding and stabilization. Other genes include the following: *MMF1*, encoding a mitochondrial matrix factor, having a possible role in mtDNA maintenance, since the deletion of this gene results in the loss of mtDNA and a decreased growth rate^22^; *ARR3*, encoding a metalloid/H^+^ antiporter, which is mainly involved in arsenite and antimonite detoxification^23^; *MCR1*, which codes for a mitochondrial NADH cytochrome b5 reductase, having an important role in the defense against oxidative stress, as it functions as a NADH-D-erythroascorbyl free radical reductase^24^; and *MDH1*, coding for the mitochondrial malate dehydrogenase, which converts malate to oxaloacetate in the TCA cycle^25^.

#### Genes activated by ZbHaa1 under copper stress

The genes whose transcriptional regulation upon copper stress is dependent on ZbHaa1 transcription factor were identified as described for acetic acid stress. Elimination of the *ZbHAA1* gene led to a reduction in the mRNA levels from 15 out of the 121 genes that were activated in the parental strain exposed to copper stress (Fig. 4, Supplementary Tables S3 and S7). These genes were classified as ZbHaa1-dependent, that is, activated either directly or indirectly by this transcription factor (Table 2). The already reported ZbHaa1 activation of the homologue of the copper-binding metallothionein encoding gene *CRS5* (ORF ZBIST_3713) in *Z. bailii*^14^ was herein confirmed. Among the identified ZbHaa1-dependent genes in the early response to copper (Table 2) is the ORF ZBIST_1696, putatively encoding a cation transport ATPase and the glutamate dehydrogenase Gdh3 encoding gene homologue (ZBIST_1594), important for the biosynthesis of glutathione, a hydroxyl radical scavenger^26^.

### Genes activated by copper and acetic acid stress

The comparison of genes that were upregulated in the parental strain during the early response to acetic acid or copper stress (Supplementary Tables S1 and S3) allowed the identification of 13 genes common to these two yeast responses. Most of these genes are homologous to *S. cerevisiae* genes that code for chaperones or co-chaperones (*HSP26, SSA3, HSP42*, and *HSP104*) necessary for protein folding and stabilization. Other genes are the homologues of (i) *TFS1*, presumably encoding an anionic phospholipid binding protein, which influences the regulation of the protein kinase A (PKA) signaling pathway, being responsible for the inhibition of the vacuolar protease CPY (carboxypeptidase Y)^27^. Expression of *TFS1* in *S. cerevisiae* is described to be elevated in response to oxidative stress^28^; (ii) *ARN2*, encoding an *S. cerevisiae* transporter required for the uptake of iron carried by the siderophore triacetylfusarinine C^29^; (iii) *BTN2*, required in *S. cerevisiae* for protein transport, regulation of pH and protein folding^30–32^; iv) *CIT1*, encoding in *S. cerevisiae* a citrate synthase, the rate-limiting enzyme of the TCA cycle^33^. Furthermore, the set of genes considered to be modulated by ZbHaa1 under acetic acid or copper stress in *Z. bailii* were also compared. From the 17 and 15 genes found to be ZbHaa1-dependent for acetic acid and copper stress, respectively, the ORF ZBIST_3873 was the only one in common. The function of this putative gene is unknown, and there is no *S. cerevisiae* homologue. However, based on its sequence similarity to *Candida albicans SOU2*, it can be hypothesized that it is related to an oxidoreductase which utilizes NADP(H) as a co-factor, although its function is still unknown^34^.

### *In silico* search for a putative ZbHaa1 DNA-binding motif

Taking into account the promoter sequences of *Z. bailii* genes homologous to *S. cerevisiae* genes considered to be directly activated by Haa1^16^ or Cup2^35^, that is, 4 from the 32 genes found in this work to be ZbHaa1-dependent (ORFs ZBIST_0079, ZBIST_3442, homologues of *S. cerevisiae HSP26*, ZBIST_0509, homologue of *S. cerevisiae YGP1*, and ZBIST_3713, homologue of *S. cerevisiae CRS5*), a putative ZbHaa1 DNA-binding motif was predicted using the Improbizer algorithm. Since only 4 genes are described to be regulated by Cup2 in *S. cerevisiae*, and only a few genes were found in common in the *S. cerevisiae* Haa1 and Cup2 regulons and dependent on ZbHaa1, the final number of promoters considered for the analysis was necessarily low in order to increase the confidence in the *in silico* predicted motif. Among the obtained motifs (data not shown), the one with the highest score (10.742) was 5′-(A/C)GGG(A/C)G(A/G)(C/T)(G/T)-3′ (Supplementary Fig. S2a). This motif was confirmed to be present in the promoter regions of seven genes found in this study to be ZbHaa1-dependent (ORFs ZBIST_0079, ZBIST_0509, ZBIST_2207, ZBIST_3442, ZBIST_3490, ZBIST_3713 and ZBIST_5024) (p-value ≤ 0.0001) (Supplementary Fig. S2b). Four of these genes’ promoters were those used for the *in silico* motif prediction. The three additional genes (ORFs ZBIST_2207, ZBIST_3490 and ZBIST_5024) that emerged from this subsequent analysis are homologues of *S. cerevisiae SSA3*, related to *FMP16*, and related to *OM45*, respectively. However, none of them is documented as directly activated by Haa1 or Cup2 in *S. cerevisiae*. Remarkably, the identified motif for ZbHaa1 was found to contain within the DNA binding sequence motif of *S. cerevisiae* Haa1 (5′-(G/C)(A/C)GG(G/C)G-3′)^17^ from position 4 to 9 (Supplementary Fig. S2a).

## Discussion

*Z. bailii* is remarkably tolerant to the yeast growth inhibitor acetic acid. The manipulation of regulatory gene networks that govern acetic acid stress tolerance in this species is essential to control acetic acid tolerance. However, this regulatory information is currently very poorly described. Herein is reported for the first time the identification of transcriptional alterations occurring during the early response of *Z. bailii* IST302 to acetic acid or copper stress and the elucidation of the regulatory network controlled by the transcription factor ZbHaa1 (homologous to *S. cerevisiae* Haa1 and Cup2 paralogues).

**Figure 5:**
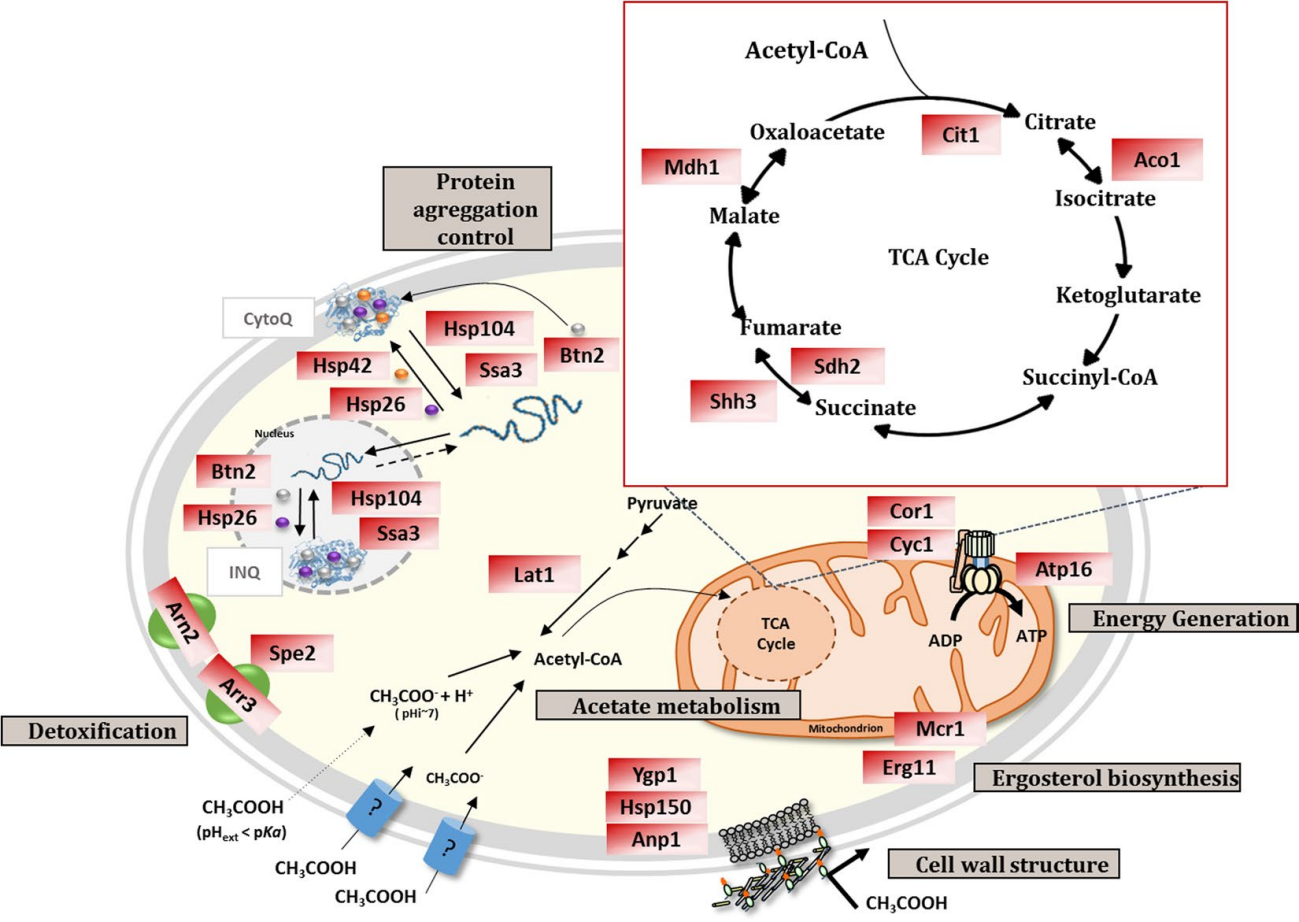
Model representing the mechanisms proposed to underlie *Z. bailii* IST302 response to acetic acid in a growth medium with glucose and acetic acid. The suggested *Z. bailii* response mechanisms to acetic acid stress include the metabolization of acetate in the presence of glucose through the TCA cycle, energy generation mechanisms, control of protein folding and stabilization and modulation of the cell wall architecture. Red boxes indicate the proteins whose encoding genes were found in this study to be upregulated in *Z. bailii* cells grown in a glucose medium supplemented with acetic acid. Details on the proteins’ function and their putative involvement in the adaptive process are described in the text.

The mRNA levels from *Z. bailii* genes homologous to *S. cerevisiae* genes involved in the tricarboxylic-acid pathway (*MDH1, ACO1, CIT1, SHH3*, and *SDH2*), energy production (*COR1, CYC1*, and *ATP16*), protein folding and stabilization (*HSP26, HSP42, HSP78, HSP104, SSA3*, and *BTN2*), biosynthesis of ergosterol (*ERG11* and *MCR1*), detoxification (*ARN2*, *SPE2*, and *ARR3*) and cell wall modulation (*YGP1, ANP1, ECM33*, and *HSP150*) were found to increase under sudden exposure to acetic acid. Taking into account the results derived from the RNA-Seq analysis, a schematic model representing the candidate processes leading to the adaptive response of *Z. bailii* IST302 to acetic acid stress is proposed (Fig. 5). Increased transcription levels from genes of the tricarboxylic acid pathway is most likely an indication of acetic acid metabolization in the presence of glucose, as previously reported^8,36^. This is considered a mechanism that can alleviate the stress caused by acetic acid by contributing to decreased acetic acid levels and increased energy production required for energy-dependent acetic acid stress detoxification mechanisms. The upregulation of genes coding for proteins involved in protein folding and stabilization, such as heat shock proteins, is an evidence of protein denaturation and misfolding, possibly as a result of intracellular acidification^37^ and oxidative stress^38,39^ induced upon acetic acid stress, likely contributing to the alleviation of the stress by refolding and reactivating the function of denatured proteins. Remarkably, four copies of *HSP26* homologues are present in *Z. bailii* IST302 genome. This high number of copies together with the registered strong increase in the transcription levels of two of them upon acetic acid stress suggest that these chaperone proteins might be relevant in *Z. bailii* tolerance to acetic acid. Significant alterations were also observed in the transcription levels from genes involved in iron metabolism (*ARN2, FET4, FRE2*, and *FRE3*) during the early adaptation of *Z. bailii* IST302 to acetic acid stress. The alteration in the expression of genes involved in iron metabolism also occurs upon weak acid stress in *S. cerevisiae* and *Z. parabailii*^7,40,41^; however, further studies are still required to understand the underlying mechanism and contribution to *Z. bailii* tolerance to acetic acid.

Among the *Z. bailii* upregulated genes upon sudden exposure to acetic acid stress is *HAL5*, encoding a putative protein kinase involved in sodium and lithium tolerance. Disruption of this gene in *S. cerevisiae* leads to increased sensitivity to cations and low pH, and impairs K^+^ uptake, having a possible role in the regulation of the K^+^ transporters Trk1 and Trk2^42^. Furthermore, the downregulation of the outward-rectifier potassium channel encoding gene homologue *TOK1* was observed in this work. This gene is described to presumably allow outward current to flow more easily than an inward current in *S. cerevisiae*^43^. A study conducted in *S. cerevisiae* has reported the positive influence of K^+^ availability in yeast tolerance to acetic acid, since cells display an increased tolerance when increasing concentrations of K^+^ are present in the growth medium^41^. Altogether, this study suggests that *Z. bailii* tolerance to acetic acid may also be improved by increasing K^+^ in the growth medium. Another interesting observation is the downregulation of several genes related to the biodegradation of fatty acids, such as the transcription factor *ADR1*, genes involved in their transport (*ANT1, PXA1, CRC1* and *AGP2*), peroxisomal matrix protein import (*PEX4, PEX12* and *PEX21*), and in β-oxidation (*POT1* and *POX1*)^44^. A possible physiological reason for this downregulation can be attributed to a higher need of fatty acids availability for sphingolipids biosynthesis, found to be increased in *Z. bailii* upon acetic acid stress exposure and having an impact in acetic acid tolerance^11^.

The rapid response of *Z. bailii* to acetic acid also suggests the reduction of mRNA levels from genes involved in cellular transport of nutrients and metals, such as phosphate (*PHO84*), zinc (*ZAP1, ZRT1* and *IZH1*), ammonium (*MEP3*, *ATO2* and *ATO3*), purines (*FCY2*), oligo-peptides (*PTR2* and *OPT1*) and amino acids (*GAP1, GAT1, STP3, BAP3, PUT4, UGA4, SHH4, BUL1, CAN1* and *VBA1*). This is indicative of the occurrence of substantial alterations in the plasma membrane transport function. In addition, acetic acid also appears to induce genes influencing cell wall architecture in *Z. bailii* such as *ANP1, ECM33*, *HSP150*, and *YGP1*. Changes in expression of genes involved in cell wall modulation upon acetic acid stress or low pH conditions (in particular *YGP1* and *HSP150*) have been reported in several studies in *S. cerevisiae*^16,40,45,46^ and more recently in the *Z. parabailii* response to lactic acid stress^7^.

**Figure 6:**
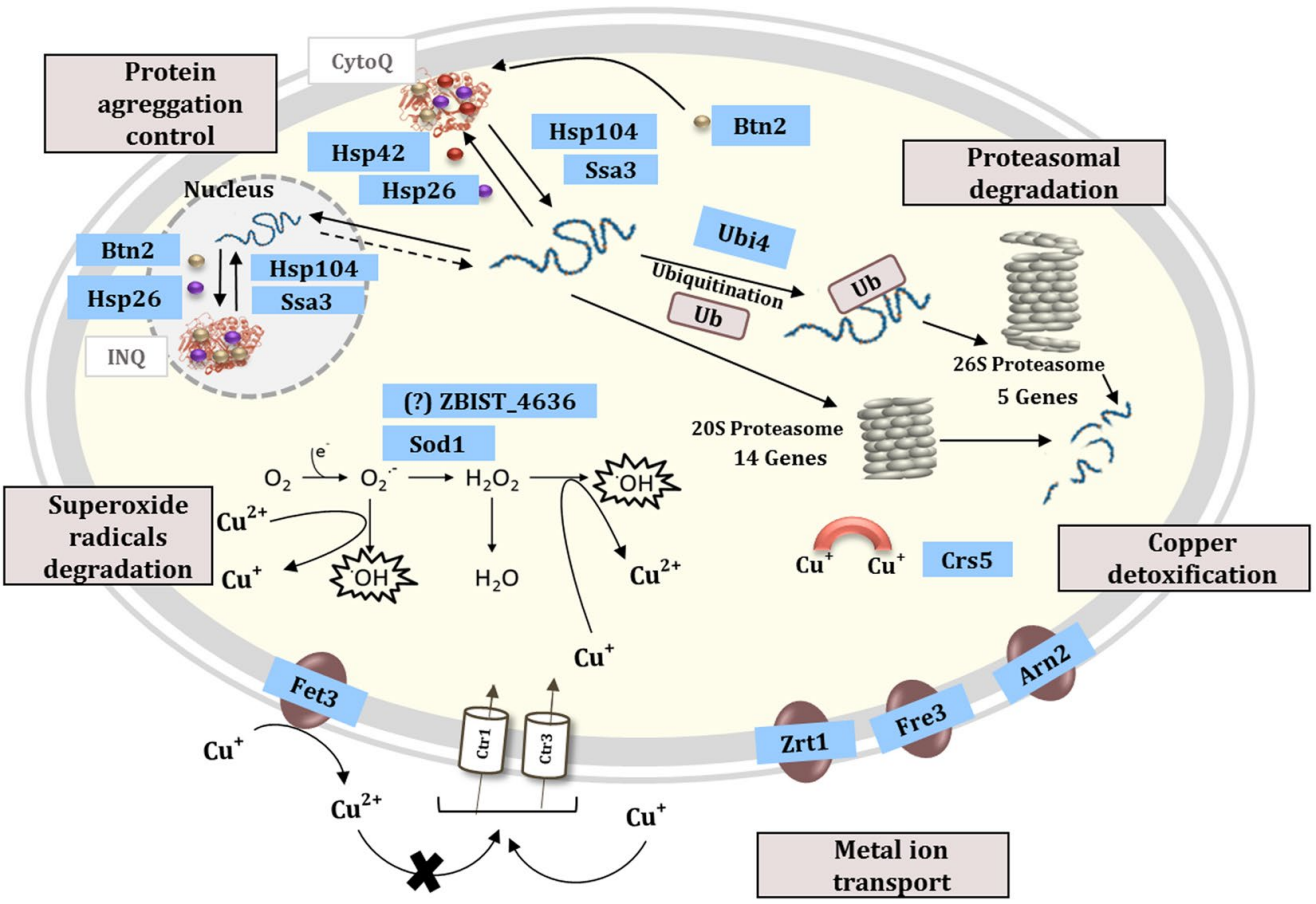
Model representing the mechanisms proposed to underlie *Z. bailii* IST302 response to copper. The suggested *Z. bailii* response mechanisms to copper stress include mechanisms of detoxification from superoxide radicals, scavenging of copper ions, control of the protein folding and stabilization and proteasomal proteolysis in response to oxidative stress. Blue boxes indicate the proteins whose encoding genes were found in this study to be upregulated in *Z. bailii* cells grown in a glucose medium supplemented with copper. Details on the proteins’ function and their putative involvement in the adaptive process are described in the text.

Regarding the adaptive response of *Z. bailii* to copper stress, taking into account the results derived from the RNA-Seq analysis, a schematic model, representing the possible processes leading to this response, is proposed in Fig. 6. The association of 18 genes to the proteasome mediated ubiquitin-dependent catabolic processes indicates the occurrence of protein denaturation derived from copper toxicity. Consistent with this idea is the increased expression of 10 genes involved in protein folding and stabilization, including chaperones and co-chaperones, such as *AHA1*, *HCH1*, *HSP26, HSP42, HSP104, SBA1*, and *SSA3*. Copper is described to be able to induce the formation of reactive oxygen species (ROS), which damage cellular components, such as proteins, nucleic acids, and membrane lipids, and the related cellular processes^47^. Some of the upregulated genes found in *Z. bailii* upon sudden copper exposure are related to the transport of heavy metal ions (*ARN2, FET3, FRE3, SIT1, SOD1*, and *ZRT1*). Fet3 is described to confer copper tolerance in *S. cerevisiae* since it is able to oxidize Cu^+^ into Cu^2+^, consequently limiting copper uptake through the Ctr1 transporter, since copper transport by this high-affinity copper transporter in *S. cerevisiae* is dependent on the previous reduction of the extracellular copper by the metalloreductases Fre1 and Fre2^48^. In *S. cerevisiae*, Fre3 is described to reduce siderophore-bound iron, whose uptake can be made by transporters such as Arn2 and Sit1^49^. Oxidative stress is documented in *S. cerevisiae* to result in the oxidation of [4Fe-4S] enzymes leading to their inactivation and the displacement of iron causing further oxidative damage. The superoxide dismutase encoded by *SOD1* has a protective role on these enzymes and prevents the accumulation of iron intracellularly^49,50^. The activation of iron transport system components suggested in the present work could, to a certain extent, allow the reconstitution of [4Fe-4S]-enzymes inactivated by oxidative stress and hindrance of copper uptake.

Copper stress induced the decrease in the mRNA levels from genes involved in the metabolism of amino acids, as well as genes associated with mitochondrial translation and respiration process. Previous studies have shown a decrease in the expression of genes related to the respiratory function and amino acids metabolic processes in *S. cerevisiae*, as a consequence of copper-induced oxidative stress^51^.

It is interesting to take notice of the different number of differently expressed genes that was found to be much higher in the mutant strain during copper stress (1301) when compared with the parental (190), or during exposure to acetic acid stress (56). These differences cannot, however, be easily explained or compared, considering that, even though we tried to use concentrations that produced an equivalent toxic effect, these stresses are inherently different and lead to significantly distinct responses in *Z. bailii*. This is evident by looking at the growth curves (based on medium turbidity and CFU/mL) of the mutant strain exposed to acetic acid or copper stress (Fig. 1b,f). Moreover, the effect of copper at the genomic expression level is apparently less specific than the effect of acetic acid, presumably due to its marked effect as a ROS inducer and the unspecific oxidation of DNA, proteins, and lipids by ROS.

**Figure 7:**
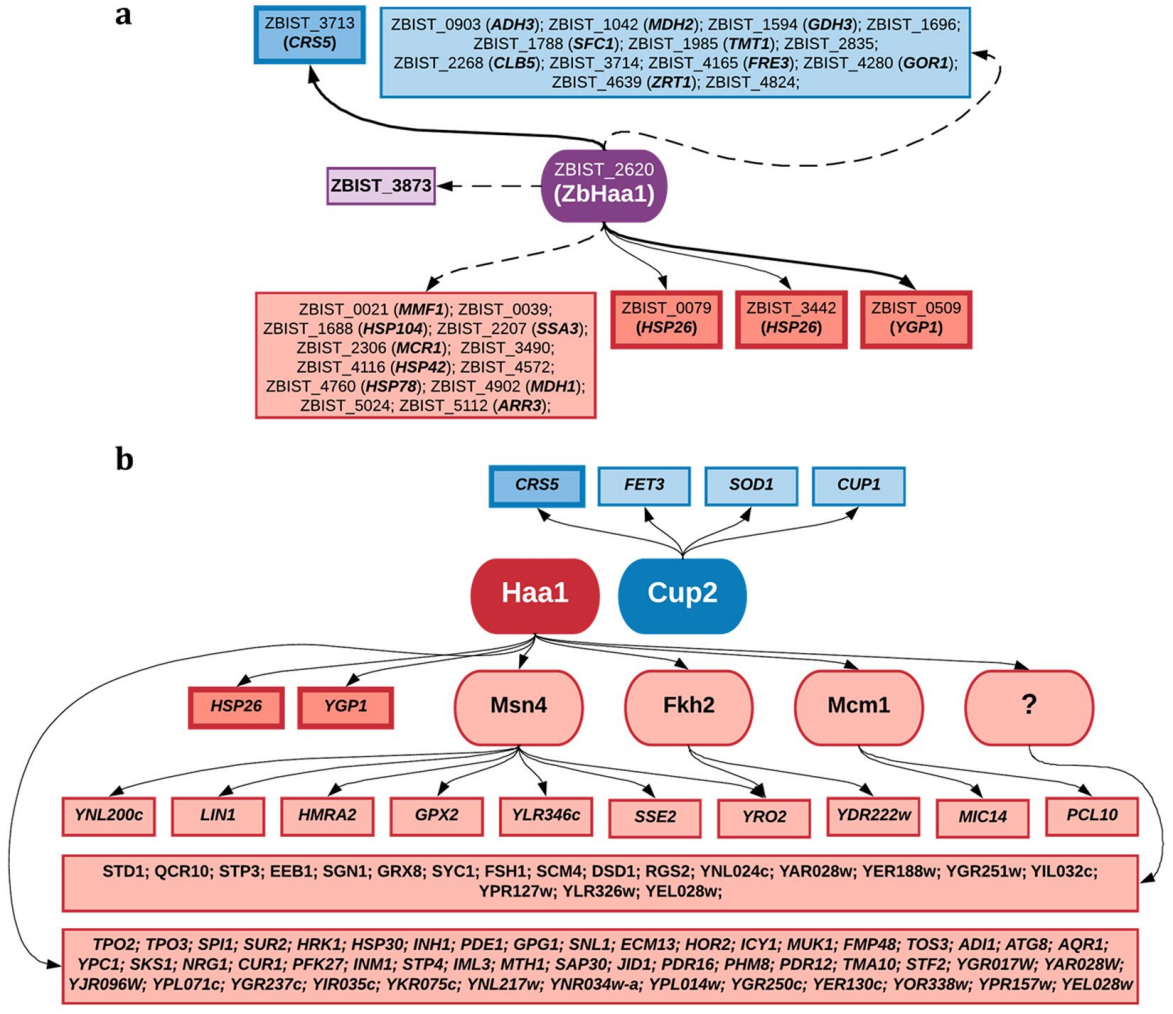
Putative regulatory networks underlying ZbHaa1-dependent gene activation in response to acetic acid or copper stress in *Z bailii*, and Haa1 and Cup2 gene activation in *S. cerevisiae* during acetic acid or copper stress, respectively. (**a**) ZbHaa1-dependent putative regulatory network. The model was assembled based on the RNA-Seq results obtained in this study and previous gene transcription studies in *Z. bailii*^14^. The displayed regulatory associations are based on the described regulatory networks of *S. cerevisiae* transcription factors Haa1 and Cup2 activated during acetic acid and copper stress responses, respectively. Represented inside boxes are the genes found to be activated in response to acetic acid (red) or copper (blue) stress in a ZbHaa1-dependent manner. *S. cerevisiae* homologous genes are shown inside brackets. Genes proposed to be transcriptionally activated under the dependence of ZbHaa1 and whose homologue in *S. cerevisiae* was described to be directly activated by Haa1 are represented inside darker boxes surrounded by thicker lines. Genes considered to be activated in a ZbHaa1-dependent manner in response to both stresses are represented inside purple boxes. Previously described regulatory associations in *S. cerevisiae* are represented by an arrow (→). *Z bailii* genes found to have stress-induced-altered transcription levels herein and in previous studies are represented by a bold arrow (→). Novel transcriptional associations are represented by a dashed arrow (→). (**b**) Haa1 and Cup2 putative regulatory networks. These networks were assembled based on documented proposed associations of Haa1 under acetic acid stress in *S. cerevisiae* (represented in red)^17^ and known targets of Cup2 under copper stress in *S. cerevisiae* (represented in blue)^35^. Genes proposed to be transcriptionally activated under the dependence of ZbHaa1 and whose homologue in *S. cerevisiae* was described to be directly activated by Haa1 are represented inside darker boxes surrounded by thicker lines. Other transcription factors presumably regulated by Haa1 and documented to regulate acetic acid-induced genes are represented inside rounded boxes. Genes identified as putative Haa1 indirect targets and whose activation is presumably dependent on unidentified transcription factors are shown associated with the transcription factor designated “?”.

A putative regulatory network dependent on the transcription factor ZbHaa1, presumably involved in the activation of genes responsible for the early transcriptional response to acetic acid or copper, is proposed in Fig. 7a. The hypothesized regulatory network was based on the results from the present study, on previously described transcriptional alterations of specific ZbHaa1-dependent genes in *Z. bailii* exposed to acetic acid stress^14^, and the information available for *S. cerevisiae* gene and genomic transcriptional regulation in the YEASTRACT database^52^. These putative regulatory associations reveal genes described to be regulated by the *S. cerevisiae* transcription factors Haa1 and Msn4 during weak acid stress, suggesting similarities in *S. cerevisiae* and *Z. bailii* adaptive responses (Fig. 7). From these networks it is also possible to find the ZbHaa1-dependent activation of the *CRS5* homologue, a gene described to be activated by Cup2 in *S. cerevisiae* in response to copper stress. This gene, together with the *YGP1* homologue (also found in this work to be ZbHaa1-dependent), is documented to have significant alterations in the mRNA levels in *Z. bailii* under copper or acetic acid stress, respectively^14^. This is consistent with the results herein presented reinforcing the previously reported bifunctionality of ZbHaa1^14^ since this transcription factor was shown to be required and have regulatory functions in the adaptive response of *Z. bailii* to acetic acid and copper stress. In *S. cerevisiae*, Haa1 and Cup2 are described as having distinct and independent functions, not showing cross-regulation^17,18^. The bifunctionality of ZbHaa1 could possibly be related to some traits of the protein. Specifically, ZbHaa1 has the same protein sequence length as Haa1 sharing 50% sequence identity, and displays the last Cys residue (residue 101) in the copper regulatory domain (CuRD), which is absent in Haa1, presumably allowing the formation of the polycopper cluster demonstrated to be required for Cup2 function^18^.

The in silico search for the predicted ZbHaa1 DNA-binding motif sequence, led to the identification of the sequence 5′-(A/C)GGG(A/C)G(A/G)(C/T)(G/T)-3′with the DNA binding sequence documented for *S. cerevisiae* Haa1 (5′-(G/C)(A/C)GG(G/C)G-3′)^17^ contained within. Since three of the four *Z. bailii* promoter sequences in the motif analysis used were from the list of genes activated under acetic acid stress, this could have biased the results and the reduced number of sequences used to identify *in silico* the motif also limits the value of the prediction. However, both of the aforementioned decision criteria were paramount in order to increase the confidence in the results, and reflect the reduced number of genes described to be regulated by Cup2 and the similarities among the genes proposed to be directly activated by Haa1 in *S. cerevisiae* and the genes considered herein to be ZbHaa1-dependent. Overall, independently of the obtained results, further studies are required to experimentally confirm the ZbHaa1 DNA binding sequence and thus allow a better understanding of the evolutionary changes that took place in the ancestral *HAA1/CUP2* orthologue following the WGD event.

In this study, it was possible to provide novel transcriptomic information regarding the genome-wide transcriptional changes occurring in the highly acetic acid resistant *Z. bailii* IST302 early response to acetic acid or copper stress. Furthermore, the ZbHaa1-dependent regulons active under acetic acid or copper stress conditions were proposed. The information gathered in this work provides new insights regarding the mechanisms underlying ZbHaa1-dependent acetic acid response, which will be useful to guide genome manipulation to develop more robust industrial strains, potentiating *Z. bailii* as a cell factory, or to guide the rational improvement of actions involving weak acid preservatives to be taken for spoilage prevention in the food industry. This study also provides interesting information deserving further analysis regarding the evolution of transcription factors and regulatory networks in pre-WGD and post-WGD yeast species.

## Materials and Methods

### Yeast strains and growth conditions

The prototrophic parental strain *Zygosaccharomyces bailii* IST302 and derived deletion mutant *Zbhaa1***∆**^14^ were used. Both strains were maintained at −80 °C in appropriate media supplemented with 15% (v/v) glycerol. Prior to use, the strains were transferred onto agar plates (2% (w/v) agar) with YPD medium (1% (w/v) yeast extract (Difco), 2% (w/v) peptone (Difco), 2% (w/v) glucose (Merck)) and grown for 24 h at 30 °C to prepare the inocula. Yeast strains were batch cultured at 30 °C, with orbital agitation (250 rpm), in liquid mineral medium (MM) containing: 0.17% (w/v) yeast nitrogen base (YNB) without amino acids or (NH_4_)_2_SO_4_ (Difco), 2% (v/v) glucose (Merck) and 0.265% (NH_4_)_2_SO_4_ (Merck), at pH 4.0. To assess the transcriptional response to acetic acid or copper in *Z. bailii* IST302 and derived deletion mutant *Zbhaa1***∆** by RNA sequencing (RNA-Seq), extraction and purification of RNA from those strains, cultivated both in the absence of stress (C1 and C2) or exposed to acetic acid stress (Ac1 and Ac2) or copper stress (Cu1 and Cu2) was performed. Cells of the parental and *Zbhaa1***∆** derived mutant strains were cultivated in MM medium until mid-exponential phase was reached (Optical density at 600 nm 0.6 ± 0.05) and subsequently re-inoculated in three flasks for each strain (C1, Ac1, and Cu1 for *Z. bailii* IST302 parental strain and C2, Ac2, and Cu2 for *Zbhaa1***∆** derived mutant) at an initial optical density of 0.2 ± 0.01 in the same unsupplemented media. After 1 hour of growth, *Z. bailii* IST302 (sample C1) and derived deletion mutant *Zbhaa1***∆** (sample C2) cultures were collected and set as the control conditions; at this point, acetic acid (adjusted to pH 4.0) or CuSO_4_ were added to the two remaining flasks of each strain culture to a final concentration of 140 mM or 0.08 mM, respectively. Acetic acid- or copper-stressed cells were harvested 1 hour after the addition of either acetic acid (samples Ac1 and Ac2, for the parental strain and *Zbhaa1***∆** strain, respectively) or copper (samples Cu1 and Cu2 for the parental strain and *Zbhaa1***∆** strain, respectively). Cells were collected by centrifugation (8000 rpm, 10 min) at 4 °C, washed twice with cold water, the pellets frozen in liquid nitrogen and then kept at −80 °C until RNA extraction.

### RNA extraction, library preparation, and sequencing

Three independent experiments were carried out. RNA extraction was performed using a modified hot phenol method^53^. Purification of RNA and DNA digestion with DNAse was performed using the commercial kit *RNA Clean & Concentrator™*-*5* (Zymo Research). Purified RNA samples were subsequently checked for quality on Fragment Analyzer (Advanced Analytical), using High Sensitivity RNA Analysis Kit (Advanced Analytical). The library preparation was performed by the Genomics Unit at Instituto Gulbenkian de Ciência (Oeiras, Portugal) using QuantSeq™ 3′ mRNA-Seq Library Prep Kit for Illumina (FWD) (Lexogen) and sequencing was performed in an Illumina HiSeq. 3000 system at the Centre for Genomic Regulation (Barcelona), obtaining 50 bp reads toward the poly(A) tail corresponding directly to the mRNA sequence.

### Transcriptomic data analysis

The pipeline implemented in this work was based on Lexogen’s recommendations for Quant-Seq. 3′mRNA data analysis. RNA-Seq analysis started with the quality trimming of the sequences using BBDuk (from the BBTools package) and quality analysis with FastQC^54^ of the raw read files obtained. The obtained libraries had an average size of 7.9 million reads, with the smallest having 6.2 million reads and the biggest 10 million. After assuring that there were no problems or relevant biases with the data, the alignment of the raw reads with a reference sequence of *Z. bailii* IST302^5^ was made. For this task, the alignment algorithm Spliced Transcripts Alignment to a Reference (STAR)^55^ was used. This algorithm allows an ultra-fast and highly accurate alignment of RNA-Seq reads to a reference genome. Following mapping of the RNA-Seq raw reads to the reference genome, the number of reads that map to a certain gene or transcript was measured making use of the script HTSeq-count, integrated in the HTSeq package, in the intersection nonempty mode^56^. Only genes achieving at least one count per million (cpm) in at least three samples from the same condition were kept for further analysis. Differential gene expression analysis was performed using the Bioconductor software package edgeR, based on the negative binomial distribution^57^. In order to consider the differences across the samples, the trimmed mean of M-values (TMM) scaling normalization was performed. Selection of the differently expressed genes was made by keeping only the entries with an adjusted p-value or false discovery rate (FDR) below 0.05 and a fold change above 1.5-fold. Posterior Gene Ontology (GO) term enrichment analyses were performed using the Blast2GO software^19^, producing a custom GO annotation for *Z. bailii* IST302 and running the Fisher Exact Test.

### *In silico* prediction of ZbHaa1 DNA-binding motif

The search for a putative ZbHaa1 motif sequence was performed using the Improbizer algorithm, which makes use of a variation of the expectation maximization algorithm^58^. This analysis only considered the promoter sequences of *Z. bailii* genes, whose transcription regulation under acetic acid or copper stress was found in this work to be ZbHaa1-dependent and that are homologues of *S. cerevisiae* genes previously considered to be directly activated by Haa1 or Cup2. Following these criteria, 4 from the 32 *Z. bailii* genes considered to be ZbHaa1-dependent upon exposure to acetic or copper stress were selected for the analysis (ORFs ZBIST_0079, ZBIST_0509, ZBIST_3442 and ZBIST_3713).

To find occurrences of the found motif in the promoters of the 32 genes considered to be ZbHaa1-dependent during the *Z. bailii* response to acetic acid or copper stress, the highest scored motif was used as input in the program ‘Find Individual Motif Occurrences’ (FIMO)^59^.

## Electronic supplementary material

Supplementary Information

## Data Availability

The datasets generated and analysed during the current study are available in NCBI’s Gene Expression Omnibus (GEO) repository, under the accession number GSE113552.
