# Supplementary material for: Transcriptional profiling of Zygosaccharomyces bailii early response to acetic acid or copper stress mediated by ZbHaa1
Source: Sci Rep. 2018 Sep 20;8:14122. doi: 10.1038/s41598-018-32266-9 (PMC6147978; doi:10.1038/s41598-018-32266-9)
Supplement: Supplementary file 1 — Supplementary Information [file 41598_2018_32266_MOESM1_ESM.pdf]

Supplementary information

Transcriptional profiling of *Zygosaccharomyces bailii* early response to acetic acid or copper stress mediated by ZbHaa1

Miguel Antunes, Margarida Palma, Isabel Sá-Correia

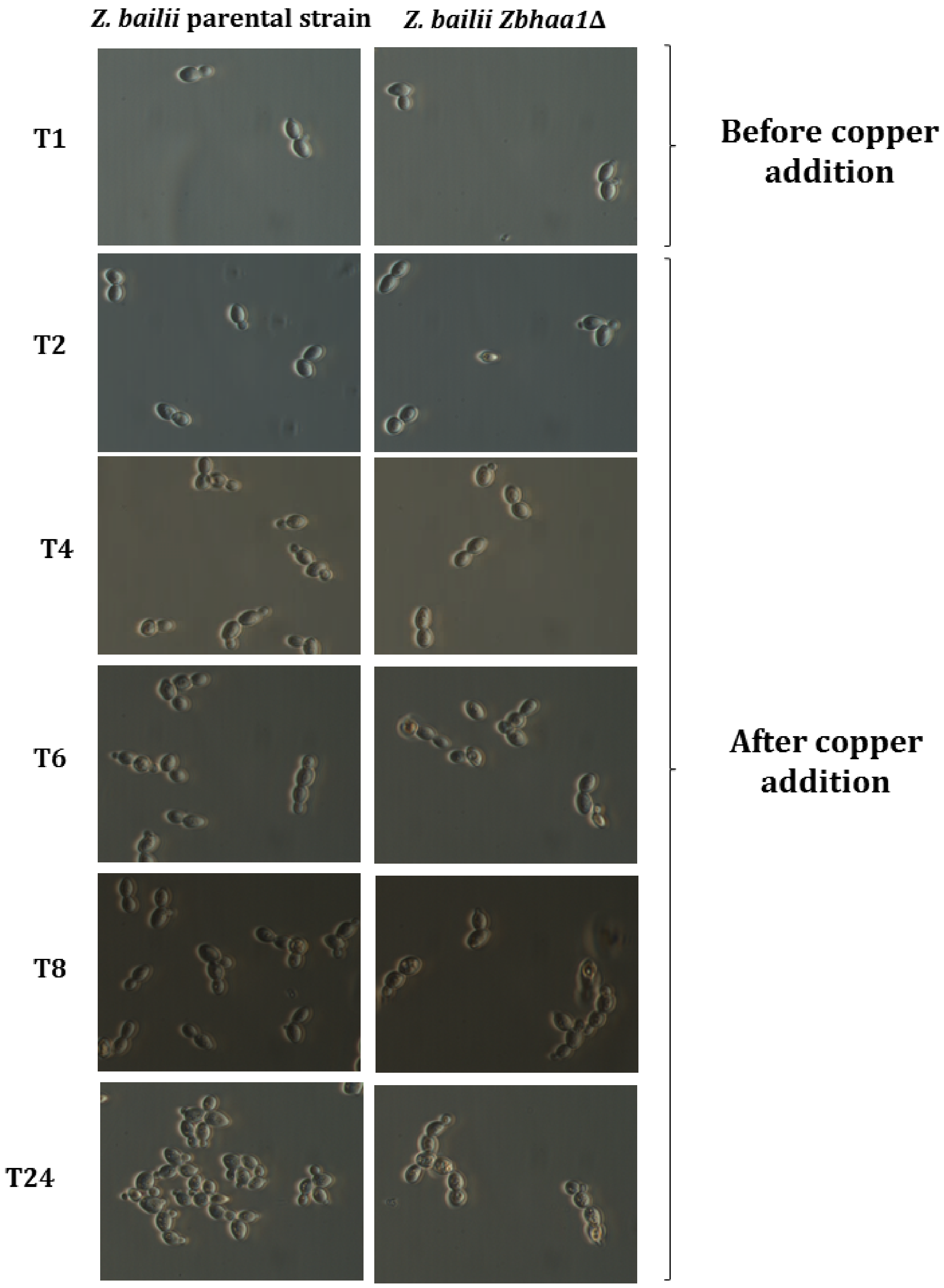

Supplementary Figure S1. Microscopic observation of *Zygosaccharomyces bailii* IST302 parental and derived deletion mutant *Zbhaa1*Δ upon copper stress. Unadapted exponentially growing cells of both strains were grown in MM medium and after 1 hour of cultivation, copper was added to the culture medium. The formation of cell aggregates was periodically observed during 24 hours using a Zeiss R Axioplan microscope (×1000 magnification). *Z. bailii* IST302 was shown not to aggregate when cultivated in either minimal or rich media [5].

a

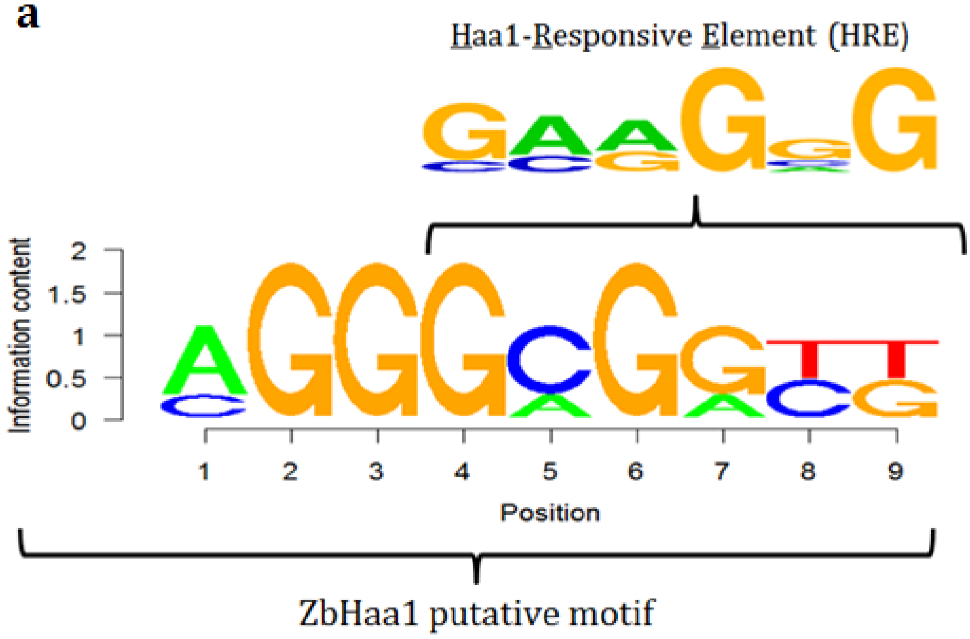

b

Motif	Sequence Name	Strand	Start	End	p-value	Matched Sequence
MGGGMGRYK	ZBIST_0079	+	682	690	9.01E-06	AGGGCGGTT
MGGGMGRYK	ZBIST_3713	+	844	852	1.05E-05	CGGGCGGCG
MGGGMGRYK	ZBIST_0079	+	166	174	1.86E-05	AGGGAGGCG
MGGGMGRYK	ZBIST_5024	-	233	241	1.86E-05	AGGGAGGCG
MGGGMGRYK	ZBIST_3442	+	414	422	3.17E-05	AGGGAGGCT
MGGGMGRYK	ZBIST_0509	+	863	871	3.17E-05	AGGGCGATG
MGGGMGRYK	ZBIST_3490	-	898	906	3.17E-05	AGGGCGACT
MGGGMGRYK	ZBIST_2207	+	813	821	6.57E-05	AGGGAGACT

Supplementary Figure S2. ZbHaa1 predicted motif sequence logo. (a) The sequence logo for the ZbHaa1 DNA binding motif sequence predicted in silico was plotted using the obtained position weight matrix. The Haa1-Responsive Element (HRE) from *S. cerevisiae*, which is contained within the predicted sequence, is also shown. **(b)** List of ORFs found to contain in their promoter region binding sites for the predicted ZbHaa1 DNA binding motif sequence.

Supplementary Table S1. Genes found to have increased mRNA levels (upregulated) in the *Z. bailii* IST302 parental strain upon sudden exposure to acetic acid stress compared with unstressed cells.

Z. bailii ORF	logFC (1)	logCPM (2)	PValue	FDR (3)	*S. cerevisiae* Homologue
ZBIST_3442	8.729419	3.0268939	1.79E-35	2.93E-32	*HSP26*
ZBIST_0079	3.307553	3.1628756	3.98E-18	1.78E-15	*HSP26*
ZBIST_5024	1.677933	8.2521984	6.94E-11	1.42E-08	*N/A*
ZBIST_0843	1.398236	4.9853194	1.96E-10	3.33E-08	*N/A*
ZBIST_4572	1.226386	7.8559731	9.34E-05	0.003703	*N/A*
ZBIST_3873	1.202736	7.0359948	3.64E-11	7.79E-09	*N/A*
ZBIST_2207	1.200679	6.3293249	1.61E-11	3.59E-09	*SSA3*
ZBIST_4116	1.182061	5.3221093	2.97E-08	3.56E-06	*HSP42*
ZBIST_1688	1.164517	6.9035211	8.09E-11	1.53E-08	*HSP104*
ZBIST_0509	1.153135	6.8658139	5.16E-08	5.4E-06	*YGP1*
ZBIST_4169	1.11627	6.7991353	3.4E-08	3.88E-06	*LAT1*
ZBIST_2654	1.087785	3.7873518	1.36E-05	0.00075	*RDI1*
ZBIST_0021	1.057352	3.8093123	2.66E-05	0.001349	*MMF1*
ZBIST_0755	1.043538	5.8801265	5.07E-08	5.4E-06	*BTN2*
ZBIST_2268	1.027389	5.5151288	4.56E-07	3.8E-05	*CLB5*
ZBIST_0280	1.025602	4.0829154	0.002426	0.045373	*AVT3*
ZBIST_5041	1.001349	7.6494402	0.00072	0.018543	*N/A*
ZBIST_2640	0.996961	8.9906119	2.64E-06	0.000183	*CYC1*
ZBIST_3271	0.980095	6.7445106	1.05E-05	0.000607	*ERG11*
ZBIST_5112	0.944859	6.3867428	1.73E-07	1.61E-05	*ARR3*
ZBIST_3929	0.916888	6.6566325	2.86E-07	2.51E-05	*LEU1*
ZBIST_1752	0.88761	4.3345747	0.000122	0.004585	*ATH1*
ZBIST_0599	0.840886	10.29752	1.97E-05	0.001032	*RPS28b*
ZBIST_3490	0.834102	6.1790462	1.39E-05	0.000761	*N/A*
ZBIST_4760	0.829551	6.3159768	6.65E-06	0.000425	*HSP78*
ZBIST_0039	0.818394	5.3861568	0.001588	0.033542	*N/A*
ZBIST_4539	0.81686	5.7265744	6.07E-05	0.002551	*SDH2*
ZBIST_4248	0.807582	6.6869887	1.91E-06	0.000136	*GIS2*
ZBIST_3988	0.803459	5.6745293	0.000226	0.007351	*N/A*
ZBIST_2306	0.795432	8.4106546	1.06E-05	0.000607	*MCR1*
ZBIST_4465	0.776017	3.8258251	0.001675	0.034611	*DHH1*
ZBIST_4782	0.773185	3.6617527	0.002187	0.041461	*N/A*
ZBIST_2636	0.771291	6.2594079	9.43E-05	0.003713	*ANP1*
ZBIST_1024	0.765359	7.9059548	3.26E-05	0.001558	*ADK1*
ZBIST_5070	0.764034	6.6432593	0.000573	0.015606	*N/A*
ZBIST_4902	0.762019	8.689291	9.31E-06	0.000558	*MDH1*
ZBIST_3414	0.74001	9.9542179	1.63E-05	0.000882	*CIT1*
ZBIST_1711	0.737264	8.6214773	6.66E-06	0.000425	*ACO1*
ZBIST_2189	0.724004	5.0919801	0.000198	0.006703	*ASE1*
ZBIST_1223	0.722157	5.2413274	0.000131	0.00473	*POS5*
ZBIST_2657	0.720058	8.6982129	1.68E-05	0.000887	*ARF2*
ZBIST_2201	0.713744	4.2871583	0.001193	0.026911	*PRS2*
ZBIST_0687	0.691111	4.5390849	0.001913	0.037789	*DON1*
ZBIST_3893	0.690201	10.218057	0.000119	0.004503	*RPL38*
ZBIST_1739	0.6785	9.4895931	0.000988	0.023701	*TIM9*
ZBIST_3292	0.668875	4.3995581	0.002589	0.047513	*YOX1*
ZBIST_0497	0.667239	6.3444569	4.74E-05	0.002139	*N/A*

ZBIST_0058	0.657506	6.4361988	0.000638	0.016685	*RBD2*
ZBIST_0844	0.656394	10.325898	0.000945	0.02291	*SHH3*
ZBIST_0037	0.656173	8.2460314	0.000338	0.009905	*TUF1*
ZBIST_0648	0.654284	6.5520389	0.00041	0.011645	*KSP1*
ZBIST_1316	0.651806	4.9811805	0.001998	0.038996	*N/A*
ZBIST_4440	0.642268	7.4864419	0.000337	0.009905	*HCR1*
ZBIST_2552	0.636736	6.5118815	0.000211	0.007055	*ARN2*
ZBIST_1806	0.630891	8.4979631	0.001151	0.026581	*COR1*
ZBIST_2846	0.629708	8.7633368	0.000861	0.021277	*ECM33*
ZBIST_4299	0.627695	6.2527659	0.0006	0.015903	*TFS1*
ZBIST_2854	0.625334	5.1001294	0.002525	0.046779	*TEC1*
ZBIST_0453	0.625212	5.858664	0.000372	0.010771	*SPE2*
ZBIST_2934	0.62392	9.761093	0.00213	0.040918	*N/A*
ZBIST_2265	0.62074	5.7758618	0.000463	0.012951	*N/A*
ZBIST_1833	0.612216	5.9665647	0.000286	0.008635	*HAL5*
ZBIST_1418	0.607828	6.2128303	0.000242	0.007616	*N/A*
ZBIST_4700	0.598434	7.9560333	0.001386	0.030442	*ATP16*
ZBIST_5073	0.596166	7.7940335	0.002205	0.041461	*PRP2*
ZBIST_2464	0.585268	5.4344173	0.001613	0.033542	*N/A*

(1) logFC - logarithm base 2 of fold change.
(2) logCPM - logarithm base 2 of counts per million.
(3) FDR - false discovery rate

Supplementary Table S2. Genes found to have decreased mRNA levels (downregulated) in the *Z. bailii* IST302 parental strain upon sudden exposure to acetic acid stress compared with unstressed cells.

Z. bailii ORF	logFC [(1)]	logCPM [(2)]	PValue	FDR [(3)]	*S. cerevisiae* Homologue
ZBIST_3145	-5.373223	4.886217	6.02E-52	1.48E-48	*N/A*
ZBIST_4788	-5.205696	9.238294	1.28E-09	2.1E-07	*N/A*
ZBIST_2710	-3.729939	3.026704	3.43E-18	2.11E-15	*N/A*
ZBIST_1449	-3.560708	10.67411	1.85E-11	4.14E-09	*N/A*
ZBIST_3799	-3.531667	0.519905	0.000192	0.006582	*N/A*
ZBIST_4935	-3.425719	7.734946	8.11E-56	3.99E-52	*N/A*
ZBIST_2194	-3.37909	0.947498	1.23E-05	0.000737	*N/A*
ZBIST_5023	-3.343291	3.674926	6.36E-20	6.26E-17	*N/A*
ZBIST_3841	-3.016466	4.357228	3.89E-24	4.79E-21	*N/A*
ZBIST_4974	-2.982861	0.71229	0.000245	0.008016	*CSI1*
ZBIST_0390	-2.972254	1.053577	2.34E-05	0.001263	*STN1*
ZBIST_3425	-2.81246	0.612571	0.000717	0.018295	*N/A*
ZBIST_3419	-2.668223	4.577845	6.24E-08	7.58E-06	*ATO2*
ZBIST_3097	-2.645348	1.34768	1.53E-05	0.000887	*N/A*
ZBIST_1850	-2.641491	1.995651	6.96E-08	7.97E-06	*N/A*
ZBIST_1856	-2.620234	0.829983	0.000402	0.012196	*N/A*
ZBIST_1885	-2.447767	4.477054	4.83E-19	3.39E-16	*GAS3*
ZBIST_4184	-2.400827	2.882413	1.42E-10	2.81E-08	*QDR2*
ZBIST_1551	-2.396118	4.96097	1.76E-11	4.12E-09	*PUT4*
ZBIST_2981	-2.339635	5.958776	8.41E-15	3.19E-12	*POT1*
ZBIST_3042	-2.277364	2.985254	4.61E-10	8.1E-08	*N/A*
ZBIST_0141	-2.272978	3.790102	1.52E-07	1.63E-05	*N/A*
ZBIST_1828	-2.261918	1.581537	2.53E-05	0.001342	*MCD4*
ZBIST_5127	-2.233173	1.843512	1.09E-05	0.000675	*PEP1*
ZBIST_0957	-2.204127	3.97145	1.69E-13	4.63E-11	*GAL7*
ZBIST_1261	-2.200105	5.123721	1.57E-19	1.28E-16	*ECM2*
ZBIST_4143	-2.192683	1.547475	5.08E-05	0.002358	*RAD10*
ZBIST_1185	-2.187226	5.383737	2.88E-17	1.58E-14	*PEX21*
ZBIST_4904	-2.050485	2.608474	1.79E-07	1.84E-05	*GPA1*
ZBIST_1396	-2.03056	4.482667	6.28E-14	1.89E-11	*KNH1*
ZBIST_1805	-1.991687	2.047118	1.51E-05	0.000886	*N/A*
ZBIST_3609	-1.966204	1.742147	0.000107	0.004166	*N/A*
ZBIST_2195	-1.941685	4.501432	2.87E-12	7.06E-10	*N/A*
ZBIST_1321	-1.876545	4.193714	7.05E-11	1.45E-08	*STL1*
ZBIST_2930	-1.868221	1.776788	0.00027	0.008734	*NDT80*
ZBIST_4481	-1.857435	1.333012	0.001121	0.025319	*MEI5*
ZBIST_3734	-1.830015	2.573066	5.46E-06	0.00039	*AGP2*
ZBIST_3173	-1.782606	2.236093	6.01E-05	0.002639	*FLR1*
ZBIST_4920	-1.781132	8.47573	0.002606	0.04858	*AQY3*
ZBIST_1746	-1.700366	6.183792	4.53E-14	1.49E-11	*N/A*
ZBIST_0429	-1.675642	1.455517	0.002067	0.041862	*TDP1*
ZBIST_2061	-1.648259	2.120923	0.000141	0.005261	*PEX4*
ZBIST_4991	-1.637144	5.572378	1.98E-12	5.12E-10	*N/A*
ZBIST_0044	-1.629572	6.683845	7.99E-17	3.58E-14	*MIG2*
ZBIST_2523	-1.625685	2.958998	2.27E-05	0.001244	*N/A*
ZBIST_3426	-1.621862	4.727163	2.75E-11	5.89E-09	*N/A*
ZBIST_1337	-1.619134	4.98135	1.08E-05	0.000675	*YBR238C*

ZBIST_4285	-1.60465	3.043639	2.77E-06	0.000207	*ISC1*
ZBIST_1595	-1.602788	8.309159	4.86E-17	2.39E-14	*N/A*
ZBIST_1629	-1.60122	6.125561	6.51E-14	1.89E-11	*YHL008C*
ZBIST_2752	-1.592199	3.108706	3.24E-06	0.000238	*N/A*
ZBIST_1606	-1.589569	2.748795	1.04E-05	0.000675	*SFL1*
ZBIST_4666	-1.568212	1.86849	0.000644	0.017137	*N/A*
ZBIST_3418	-1.551564	7.738106	2.13E-15	8.72E-13	*ATO2*
ZBIST_1907	-1.550499	9.597124	1.04E-05	0.000675	*GAL2*
ZBIST_3431	-1.528915	4.230515	2.16E-08	2.87E-06	*GLC3*
ZBIST_3836	-1.516754	2.65687	4.18E-05	0.002055	*N/A*
ZBIST_1127	-1.513548	2.947326	1.22E-05	0.000737	*UCC1*
ZBIST_2740	-1.503142	2.625088	5.31E-05	0.00242	*MLH3*
ZBIST_5011	-1.492601	3.350009	6.67E-06	0.000469	*CAT8*
ZBIST_0305	-1.491042	2.628006	6.56E-05	0.002834	*HSM3*
ZBIST_1911	-1.478456	3.090061	3.19E-05	0.001633	*ERC1*
ZBIST_1259	-1.464623	2.565955	0.000141	0.005261	*RKM1*
ZBIST_2819	-1.447439	3.540963	1.51E-06	0.000126	*N/A*
ZBIST_2744	-1.434357	2.651351	9.26E-05	0.003767	*N/A*
ZBIST_4235	-1.412946	2.678043	0.000151	0.005509	*SLA2*
ZBIST_2058	-1.390491	5.889464	2.78E-10	5.27E-08	*N/A*
ZBIST_2880	-1.37896	4.44645	4.1E-08	5.32E-06	*CRC1*
ZBIST_0234	-1.376582	3.905643	1.26E-06	0.000109	*MMM1*
ZBIST_4547	-1.374872	7.047709	1.36E-09	2.16E-07	*SKS1*
ZBIST_4112	-1.372011	3.126631	2.66E-05	0.001394	*ECM3*
ZBIST_4723	-1.364539	2.240303	0.00086	0.021071	*APC11*
ZBIST_2713	-1.352071	3.930398	1.07E-05	0.000675	*PXA1*
ZBIST_2974	-1.349863	2.541072	0.000357	0.01105	*IMP2'*
ZBIST_2659	-1.341895	4.682113	6.32E-08	7.58E-06	*SNF3*
ZBIST_4949	-1.335254	3.536306	1.1E-05	0.000675	*YLL058W*
ZBIST_1552	-1.33483	4.927694	8.72E-08	9.75E-06	*CIN1*
ZBIST_2206	-1.313318	2.682692	0.000246	0.008016	*AAR2*
ZBIST_4030	-1.309809	2.253075	0.001909	0.039472	*N/A*
ZBIST_4344	-1.304387	2.246199	0.002407	0.045747	*KAR4*
ZBIST_2551	-1.302087	2.834477	0.000415	0.012445	*FRE2*
ZBIST_1422	-1.289184	6.420208	2.79E-09	4.17E-07	*TDA1*
ZBIST_4973	-1.287846	3.523018	3.62E-05	0.001819	*PEX12*
ZBIST_2077	-1.286747	4.942531	1.31E-07	1.43E-05	*MCH4*
ZBIST_5139	-1.276982	2.603835	0.00051	0.014507	*N/A*
ZBIST_2564	-1.26458	4.261981	1.3E-06	0.00011	*GUT1*
ZBIST_2297	-1.260108	2.789398	0.000354	0.011043	*NAS2*
ZBIST_2987	-1.257333	3.510968	4.61E-05	0.002205	*N/A*
ZBIST_0863	-1.25674	2.852568	0.00033	0.010403	*N/A*
ZBIST_5098	-1.254316	6.144829	8.04E-09	1.13E-06	*MATALPHA1*
ZBIST_5050	-1.243828	2.736153	0.000621	0.016804	*YRM1*
ZBIST_0196	-1.235542	3.62091	9.17E-05	0.003762	*N/A*
ZBIST_3176	-1.23243	3.14424	0.000231	0.00762	*FLR1*
ZBIST_1400	-1.230067	7.140869	9.73E-10	1.65E-07	*FBP1*
ZBIST_2797	-1.216271	4.060134	4.49E-06	0.000325	*YIA6*
ZBIST_4186	-1.215402	2.703042	0.001046	0.024513	*MTQ1*
ZBIST_1271	-1.202393	3.257945	0.000154	0.005542	*PUS6*

ZBIST_0249	-1.192371	3.670021	8.05E-05	0.003387	*SAG1*
ZBIST_1438	-1.18076	2.450833	0.002144	0.043081	*DYN3*
ZBIST_1980	-1.17323	7.486322	3.29E-10	6E-08	*PFK27*
ZBIST_3187	-1.169479	2.787445	0.002185	0.043192	*BRR6*
ZBIST_2614	-1.164479	4.010677	1.91E-05	0.001081	*ICL2*
ZBIST_1388	-1.158371	6.725694	3.62E-09	5.24E-07	*ADR1*
ZBIST_5123	-1.157317	3.316526	0.000157	0.005589	*PDR12*
ZBIST_4740	-1.15609	3.798472	4.15E-05	0.002055	*THI20*
ZBIST_3772	-1.137732	4.255028	2.09E-05	0.001158	*N/A*
ZBIST_2975	-1.132551	7.389992	2.43E-09	3.74E-07	*GUT2*
ZBIST_3381	-1.12813	4.515656	1.5E-05	0.000886	*IRC20*
ZBIST_4123	-1.124209	2.593371	0.002001	0.041046	*THI72*
ZBIST_1685	-1.1125	5.569081	2.62E-06	0.000198	*TPO1*
ZBIST_1910	-1.105377	5.367317	7.92E-07	6.96E-05	*YNG2*
ZBIST_2958	-1.104073	3.97569	7.02E-05	0.003006	*WHI5*
ZBIST_5015	-1.103556	2.893322	0.00111	0.025319	*YKU70*
ZBIST_0773	-1.093583	4.026025	4.55E-05	0.002196	*VBA1*
ZBIST_4634	-1.092293	3.58997	0.000337	0.010559	*N/A*
ZBIST_0049	-1.087484	5.400479	0.000457	0.013383	*POX1*
ZBIST_5101	-1.085602	3.232213	0.0007	0.018026	*FET4*
ZBIST_4799	-1.08144	4.663632	1.05E-05	0.000675	*RCH1*
ZBIST_2040	-1.081219	8.14084	2.41E-06	0.000189	*GAP1*
ZBIST_1394	-1.078675	6.549173	2.16E-06	0.000175	*STP3*
ZBIST_4266	-1.077257	3.14277	0.000918	0.022031	*IST1*
ZBIST_3364	-1.072594	6.010875	2.04E-07	2.05E-05	*GSY1*
ZBIST_1840	-1.072122	3.678651	0.000298	0.009523	*HNM1*
ZBIST_2284	-1.068352	3.975013	0.000104	0.004136	*HFI1*
ZBIST_4988	-1.064246	3.428691	0.000367	0.011298	*OPT1*
ZBIST_2243	-1.060219	6.114534	4.1E-07	3.74E-05	*MEP3*
ZBIST_3686	-1.057112	2.844484	0.002028	0.041412	*N/A*
ZBIST_2448	-1.048028	4.544286	1.74E-05	0.000996	*N/A*
ZBIST_2891	-1.045865	4.341476	0.000293	0.00944	*RGS2*
ZBIST_4639	-1.043397	7.758469	1.27E-08	1.74E-06	*ZRT1*
ZBIST_4152	-1.025813	4.886935	2.7E-05	0.001398	*RPM2*
ZBIST_0993	-1.024498	6.301795	5.54E-07	4.96E-05	*IZH1*
ZBIST_0531	-1.016864	7.100088	6.52E-08	7.65E-06	*GSF2*
ZBIST_3259	-1.011368	4.616629	5.82E-05	0.002602	*MCM5*
ZBIST_3690	-1.010656	3.771024	0.001038	0.024443	*ROX1*
ZBIST_0205	-1.007296	4.962904	4.93E-05	0.002332	*OKP1*
ZBIST_3700	-1.000503	3.138521	0.002337	0.044902	*HOS1*
ZBIST_4345	-0.99382	4.473062	8.31E-05	0.003466	*N/A*
ZBIST_0587	-0.993598	4.366849	0.000188	0.006504	*N/A*
ZBIST_1764	-0.990282	7.006688	2.31E-07	2.23E-05	*BNA2*
ZBIST_2590	-0.98904	4.615534	5.41E-05	0.002441	*TAF3*
ZBIST_3061	-0.984693	4.712448	7.78E-05	0.003302	*HYM1*
ZBIST_3890	-0.983736	4.41725	0.000131	0.004994	*N/A*
ZBIST_2726	-0.982211	3.458344	0.000982	0.023468	*RRD2*
ZBIST_3170	-0.981015	9.325083	3.08E-07	2.92E-05	*TPO1*
ZBIST_0757	-0.980286	4.210295	0.000139	0.005247	*CBF2*
ZBIST_1360	-0.975837	5.752163	0.00023	0.00762	*GSM1*

ZBIST_3812	-0.974895	4.050521	0.000177	0.006167	*ARH1*
ZBIST_4810	-0.972153	4.052412	0.000445	0.013104	*ATG1*
ZBIST_1215	-0.965062	4.898877	0.000109	0.004225	*N/A*
ZBIST_4985	-0.964013	5.242555	3.35E-05	0.0017	*SOK2*
ZBIST_0909	-0.962675	3.737883	0.001112	0.025319	*FUR4*
ZBIST_1617	-0.961788	4.170672	0.000492	0.014184	*TOK1*
ZBIST_4167	-0.95185	4.817542	5.87E-05	0.002602	*FRE3*
ZBIST_3689	-0.948696	6.856372	2.42E-06	0.000189	*VID24*
ZBIST_3520	-0.943834	6.588125	1.69E-06	0.000139	*ZRT1*
ZBIST_0955	-0.941926	4.472573	0.000229	0.00762	*GAL1*
ZBIST_2632	-0.939707	3.709725	0.001458	0.031471	*BUL1*
ZBIST_3586	-0.937245	4.055684	0.000729	0.018457	*CST6*
ZBIST_3717	-0.936937	4.38503	0.000321	0.010191	*MUD1*
ZBIST_4159	-0.93227	4.288449	0.000616	0.016764	*PHO84*
ZBIST_2436	-0.922975	3.451433	0.00218	0.043192	*N/A*
ZBIST_0692	-0.914953	3.458969	0.002513	0.047395	*MGA1*
ZBIST_3824	-0.913485	4.304791	0.000644	0.017137	*UGA4*
ZBIST_0204	-0.908792	3.76721	0.002261	0.044508	*N/A*
ZBIST_2830	-0.906264	5.477164	9.02E-05	0.003729	*BAP3*
ZBIST_3830	-0.901412	4.233196	0.000404	0.012196	*N/A*
ZBIST_3017	-0.887844	3.882841	0.00106	0.024618	*ECM7*
ZBIST_2328	-0.886087	5.980855	1.96E-05	0.001096	*SSU1*
ZBIST_2554	-0.884118	4.369622	0.000712	0.018246	*N/A*
ZBIST_4506	-0.879828	5.087505	0.000158	0.005604	*N/A*
ZBIST_2101	-0.876685	4.076415	0.000643	0.017137	*N/A*
ZBIST_0753	-0.87504	4.436474	0.000424	0.012615	*KRE6*
ZBIST_5065	-0.8742	3.976025	0.00118	0.026281	*YIL166C*
ZBIST_4244	-0.862513	5.323964	0.000152	0.005509	*NRD1*
ZBIST_1927	-0.858034	4.452908	0.000865	0.021085	*SSH4*
ZBIST_1053	-0.855396	4.4703	0.00113	0.0254	*SKP2*
ZBIST_0711	-0.847131	9.046632	0.000666	0.017527	*OYE2*
ZBIST_2919	-0.839233	4.398767	0.001146	0.025643	*NAF1*
ZBIST_2764	-0.838279	4.348644	0.000693	0.018026	*MET32*
ZBIST_3880	-0.828361	4.579213	0.000741	0.018504	*SGE1*
ZBIST_1556	-0.827635	4.901722	0.000735	0.018457	*SOG2*
ZBIST_4879	-0.824776	3.993322	0.001724	0.036102	*MRPL23*
ZBIST_1300	-0.824417	4.248971	0.001194	0.026464	*IPK1*
ZBIST_4923	-0.818485	4.840383	0.001586	0.033801	*YPL088W*
ZBIST_4270	-0.814468	4.029662	0.002164	0.043192	*CAN1*
ZBIST_4592	-0.811815	5.834574	0.001211	0.026613	*N/A*
ZBIST_3870	-0.806563	4.49349	0.00123	0.026902	*TNA1*
ZBIST_2124	-0.802251	4.052478	0.002293	0.044734	*RIM20*
ZBIST_0834	-0.799288	4.757361	0.000995	0.023665	*ZAP1*
ZBIST_4789	-0.797214	6.159633	9.94E-05	0.003976	*N/A*
ZBIST_1133	-0.79634	8.308164	9.37E-06	0.000641	*HTA2*
ZBIST_4441	-0.791404	5.580065	0.000783	0.01926	*N/A*
ZBIST_3428	-0.789438	6.004834	0.000161	0.005677	*SNF3*
ZBIST_1158	-0.77562	4.367331	0.002042	0.041541	*UGA4*
ZBIST_4132	-0.771156	6.944543	6.37E-05	0.002776	*DLD3*
ZBIST_1994	-0.768761	4.605125	0.002582	0.048512	*SPO71*

ZBIST_2605	-0.767559	5.534644	0.000464	0.013504	*CIT3*
ZBIST_4996	-0.750765	5.907459	0.001028	0.024317	*PDR15*
ZBIST_3582	-0.749517	4.833635	0.001601	0.033967	*N/A*
ZBIST_4119	-0.748069	4.649896	0.002319	0.044902	*DIA1*
ZBIST_1389	-0.742365	7.004017	0.000125	0.004788	*MDM30*
ZBIST_4874	-0.737918	5.63421	0.00121	0.026613	*PDR15*
ZBIST_2279	-0.734841	4.908438	0.001932	0.039792	*N/A*
ZBIST_4756	-0.72818	4.937891	0.001625	0.034197	*BUB3*
ZBIST_3358	-0.726269	5.482432	0.001883	0.039101	*N/A*
ZBIST_2565	-0.702103	5.412359	0.002297	0.044734	*GAT1*
ZBIST_1262	-0.700105	6.203408	0.000397	0.012123	*NRG2*
ZBIST_5021	-0.695006	7.256035	0.000227	0.00762	*IMP4*
ZBIST_4205	-0.692531	5.368271	0.001741	0.036309	*PFK26*
ZBIST_2623	-0.69059	7.621075	0.000193	0.006582	*SUT1*
ZBIST_3577	-0.686648	5.0088	0.002592	0.048512	*N/A*
ZBIST_3521	-0.684191	9.960386	0.000659	0.017434	*PDR15*
ZBIST_2477	-0.682826	7.715425	0.000609	0.016764	*FCY2*
ZBIST_2358	-0.672698	6.704153	0.000425	0.012615	*SSU72*
ZBIST_2991	-0.672271	8.095375	0.000194	0.00659	*N/A*
ZBIST_1559	-0.664141	6.987775	0.000697	0.018026	*CLN3*
ZBIST_2872	-0.664031	6.837614	0.000616	0.016764	*ECM3*
ZBIST_4074	-0.664009	9.320715	0.000152	0.005509	*N/A*
ZBIST_3546	-0.65478	8.16517	0.000524	0.014837	*N/A*
ZBIST_0334	-0.650755	5.486822	0.002299	0.044734	*CIN4*
ZBIST_3549	-0.647506	6.012916	0.001626	0.034197	*N/A*
ZBIST_4458	-0.647052	5.879633	0.002331	0.044902	*HMX1*
ZBIST_1618	-0.642919	7.681505	0.000672	0.017588	*ATO3*
ZBIST_1748	-0.636364	6.339195	0.001078	0.024909	*BNA4*
ZBIST_2175	-0.630019	6.709264	0.001415	0.030691	*MTH1*
ZBIST_1448	-0.619602	6.674728	0.001472	0.031643	*PXR1*
ZBIST_1982	-0.603813	7.598643	0.001088	0.025019	*HRT1*
ZBIST_4580	-0.602355	6.232129	0.002486	0.047063	*FAP1*
ZBIST_2592	-0.596323	6.518064	0.002364	0.045092	*MRPS16*
ZBIST_2255	-0.594847	6.144583	0.0021	0.042363	*ANT1*

(1) logFC - logarithm base 2 of fold change.
(2) logCPM - logarithm base 2 of counts per million.
(3) FDR - false discovery rate

Supplementary Table S3. Genes found to have increased mRNA levels (upregulated) in the *Z. bailii* IST302 parental strain upon sudden exposure to copper stress compared with unstressed cells.

Z. bailii ORF	logFC [(1)]	logCPM [(2)]	PValue	FDR [(3)]	*S. cerevisiae* Homologue
ZBIST_3442	7.823515	2.205778	8.42E-09	5.2E-06	*HSP26*
ZBIST_1696	4.496473	7.11577	2.5E-18	1.23E-14	*N/A*
ZBIST_4636	4.310379	8.510022	2.39E-15	5.89E-12	*N/A*
ZBIST_3714	4.130869	3.159119	6.58E-11	6.5E-08	*N/A*
ZBIST_3713	2.799856	13.89999	1.07E-11	1.33E-08	*N/A*
ZBIST_4639	2.668715	9.990065	4.93E-14	8.11E-11	*ZRT1*
ZBIST_0079	2.476618	2.452213	1.51E-05	0.002763	*HSP26*
ZBIST_1985	2.090656	3.62308	6.22E-08	2.92E-05	*TMT1*
ZBIST_1233	2.014263	2.464921	0.000105	0.008361	*N/A*
ZBIST_2634	1.760227	9.243613	6.52E-09	5.2E-06	*N/A*
ZBIST_0576	1.618519	2.989571	0.000694	0.024632	*FLR1*
ZBIST_2107	1.607063	5.023772	2.03E-07	8.36E-05	*FRE3*
ZBIST_4635	1.45051	3.585837	1.22E-05	0.002319	*N/A*
ZBIST_4946	1.37821	7.350101	8.03E-09	5.2E-06	*N/A*
ZBIST_2207	1.369144	6.439242	1.51E-08	8.31E-06	*SSA3*
ZBIST_2536	1.297456	3.593518	0.000246	0.013531	*ARC1*
ZBIST_1600	1.259201	4.426722	1.74E-05	0.002917	*N/A*
ZBIST_1314	1.233493	10.00152	6.5E-08	2.92E-05	*FET3*
ZBIST_4824	1.192651	3.745682	0.000126	0.009341	*N/A*
ZBIST_1232	1.160372	4.582199	3.69E-05	0.004788	*N/A*
ZBIST_4305	1.070926	6.69427	0.000442	0.018501	*FRM2*
ZBIST_3873	1.067237	6.934981	4.29E-07	0.000163	*N/A*
ZBIST_4223	1.058935	7.061125	0.000529	0.020873	*SHP1*
ZBIST_1191	1.056463	3.766288	0.000672	0.024197	*MUP1*
ZBIST_2552	1.020678	6.747364	7.64E-07	0.000251	*ARN2*
ZBIST_1797	1.009705	11.3934	8.57E-06	0.002045	*SOD1*
ZBIST_0755	0.977263	5.832341	0.00012	0.009225	*BTN2*
ZBIST_1305	0.963121	4.585489	0.000101	0.008329	*PCL5*
ZBIST_1140	0.959655	4.911337	3.98E-05	0.005024	*MGT1*
ZBIST_2654	0.952244	3.683578	0.001944	0.047825	*RDI1*
ZBIST_3049	0.941975	4.068444	0.000262	0.013632	*SDS22*
ZBIST_2714	0.939463	7.122859	4.19E-06	0.001201	*N/A*
ZBIST_4116	0.939401	5.154194	6.65E-05	0.006519	*HSP42*
ZBIST_4133	0.935641	6.390907	6.67E-06	0.001733	*THI3*
ZBIST_2242	0.933551	4.141259	0.000982	0.031487	*N/A*
ZBIST_2268	0.92442	5.440316	1.94E-05	0.003082	*CLB5*
ZBIST_2715	0.917719	5.751994	8.81E-05	0.00763	*SPT10*
ZBIST_2632	0.914867	4.512804	0.000422	0.018298	*BUL1*
ZBIST_3053	0.900299	7.24382	4.46E-05	0.005236	*RPN13*
ZBIST_4866	0.898882	7.627759	3.53E-05	0.004761	*PUP1*
ZBIST_3560	0.898491	6.39031	1.12E-05	0.002319	*FCY2*
ZBIST_2210	0.882232	4.934524	0.000734	0.025529	*ATG8*
ZBIST_2340	0.861153	8.478076	2.59E-05	0.003877	*ZWF1*
ZBIST_4042	0.85785	5.429936	0.000331	0.015878	*N/A*
ZBIST_4766	0.847741	4.541763	0.000249	0.013531	*SIT1*
ZBIST_2671	0.842599	4.38836	0.000494	0.020002	*RNY1*
ZBIST_4814	0.83226	5.469439	0.000173	0.011315	*STR3*

ZBIST_1801	0.830583	8.488864	6.69E-05	0.006519	*PRE7*
ZBIST_1594	0.828167	11.08737	8.62E-05	0.0076	*GDH3*
ZBIST_2267	0.827287	4.586817	0.000768	0.026321	*N/A*
ZBIST_4135	0.827084	8.340171	6.74E-05	0.006519	*ARN2*
ZBIST_1083	0.825434	6.037323	6.12E-05	0.006428	*CDD1*
ZBIST_1003	0.824576	8.567065	9.37E-05	0.007973	*HCH1*
ZBIST_0347	0.824071	4.631579	0.000873	0.028912	*N/A*
ZBIST_0903	0.819982	8.676166	1.73E-05	0.002917	*ADH3*
ZBIST_1688	0.818083	6.66825	5.24E-05	0.005795	*HSP104*
ZBIST_2835	0.812063	5.863223	7.48E-05	0.006837	*N/A*
ZBIST_0500	0.810568	7.506169	3.37E-05	0.004756	*N/A*
ZBIST_4280	0.809392	10.65634	4.07E-05	0.005024	*GOR1*
ZBIST_2141	0.808058	6.655521	0.000199	0.011968	*SEM1*
ZBIST_0943	0.804957	5.209092	0.001866	0.04699	*ADH1*
ZBIST_1918	0.800097	8.008719	0.000269	0.01371	*N/A*
ZBIST_1958	0.777009	5.510824	0.000218	0.012988	*PSF3*
ZBIST_4542	0.775763	7.90324	4.25E-05	0.005113	*UBI4*
ZBIST_3475	0.771447	5.031716	0.000635	0.023633	*N/A*
ZBIST_0295	0.76842	4.641455	0.001347	0.03734	*N/A*
ZBIST_3008	0.76298	4.590647	0.00186	0.04699	*HXK1*
ZBIST_4165	0.754371	4.440518	0.001149	0.033948	*FRE3*
ZBIST_0926	0.751239	7.110071	3.57E-05	0.004761	*PDX3*
ZBIST_4081	0.750102	12.99739	0.000322	0.015566	*CPR1*
ZBIST_1575	0.749683	9.079261	0.000196	0.011968	*PRE10*
ZBIST_0799	0.739506	4.402658	0.001884	0.047212	*N/A*
ZBIST_3252	0.737964	5.621591	0.000449	0.018639	*ECI1*
ZBIST_0877	0.737952	9.522299	0.000307	0.01514	*SBA1*
ZBIST_2148	0.736103	7.377317	0.000233	0.013376	*GCY1*
ZBIST_2153	0.732489	9.519747	0.000148	0.009976	*PFY1*
ZBIST_1433	0.725766	8.624018	0.001779	0.046218	*PRC1*
ZBIST_2142	0.722691	9.028687	0.000198	0.011968	*RPT5*
ZBIST_2935	0.722658	4.706475	0.001174	0.0345	*YNL134C*
ZBIST_4299	0.720262	6.299524	0.000248	0.013531	*TFS1*
ZBIST_4317	0.716791	9.003771	0.000107	0.008361	*GRX2*
ZBIST_3796	0.715539	6.907702	6.94E-05	0.006587	*PRE6*
ZBIST_3438	0.712698	6.325419	0.002079	0.049819	*RHB1*
ZBIST_5004	0.705806	9.236707	0.000174	0.011315	*DSK2*
ZBIST_3926	0.70553	8.560767	0.000198	0.011968	*SCL1*
ZBIST_4217	0.705326	7.882935	0.000259	0.013606	*PUP3*
ZBIST_0705	0.703475	7.415334	0.000128	0.009341	*PUP2*
ZBIST_3759	0.702956	7.850502	0.00071	0.024847	*RPN7*
ZBIST_3640	0.700191	8.539127	0.000197	0.011968	*TPM1*
ZBIST_2066	0.695669	7.217684	0.000183	0.011745	*PRE9*
ZBIST_0497	0.690589	6.350673	9.93E-05	0.008307	*N/A*
ZBIST_1694	0.688209	4.951063	0.000938	0.030654	*HIF1*
ZBIST_3545	0.680506	7.858802	0.000272	0.013711	*TRX3*
ZBIST_2312	0.679552	6.146251	0.000236	0.013376	*UMP1*
ZBIST_1116	0.676529	8.895813	0.001087	0.032709	*AHA1*
ZBIST_3835	0.675367	8.737428	0.000314	0.015351	*UBP6*
ZBIST_1054	0.668558	5.606677	0.000642	0.023633	*GAS4*

ZBIST_0166	0.662294	11.99097	0.000748	0.025804	*TAL1*
ZBIST_0917	0.651275	6.852576	0.000511	0.020351	*ETR1*
ZBIST_0240	0.647446	9.626318	0.000344	0.016051	*PRE3*
ZBIST_3184	0.645743	8.423683	0.000267	0.01371	*PRE4*
ZBIST_4146	0.644681	9.073926	0.000358	0.016051	*PRE8*
ZBIST_1042	0.64225	7.650646	0.00023	0.013376	*MDH2*
ZBIST_4561	0.6406	6.68176	0.000297	0.01482	*PBI2*
ZBIST_1401	0.637887	5.649649	0.00158	0.042627	*PBP4*
ZBIST_4136	0.62917	6.562976	0.001517	0.041147	*N/A*
ZBIST_1788	0.627305	6.842054	0.000347	0.016051	*SFC1*
ZBIST_0627	0.627006	7.48968	0.000442	0.018501	*PRE5*
ZBIST_2425	0.620848	8.833115	0.001839	0.04699	*INH1*
ZBIST_3289	0.62016	10.5511	0.000976	0.031477	*TSA1*
ZBIST_3398	0.618903	7.833489	0.000796	0.027062	*LEU2*
ZBIST_4458	0.618714	6.421148	0.00108	0.032709	*HMX1*
ZBIST_4777	0.618666	6.922905	0.0009	0.029604	*PCM1*
ZBIST_2729	0.617689	8.604434	0.0008	0.027062	*PEP4*
ZBIST_1306	0.614915	7.002255	0.001056	0.03257	*N/A*
ZBIST_1863	0.61462	7.279318	0.001025	0.032513	*DDP1*
ZBIST_3754	0.614468	8.299375	0.00051	0.020351	*PRE2*
ZBIST_4845	0.608561	6.890721	0.001108	0.032955	*GDB1*
ZBIST_3414	0.605542	9.865249	0.001282	0.036157	*CIT1*
ZBIST_4751	0.600567	7.646325	0.000567	0.021698	*N/A*
ZBIST_4004	0.592148	7.666518	0.00124	0.035793	*N/A*

(1) logFC - logarithm base 2 of fold change.
(2) logCPM - logarithm base 2 of counts per million.
(3) FDR - false discovery rate

Supplementary Table S4. Genes found to have decreased mRNA levels (downregulated) in the *Z. bailii* IST302 parental strain upon sudden exposure to copper stress compared with unstressed cells.

Z. bailii ORF	logFC [(1)]	logCPM [(2)]	PValue	FDR [(3)]	*S. cerevisiae* Homologue
ZBIST_3690	-2.491078	3.404912	1.91E-06	0.00059	*ROX1*
ZBIST_0760	-1.539179	4.234835	0.000129	0.009341	*CWP1*
ZBIST_1699	-1.378345	2.754248	0.001047	0.032513	*N/A*
ZBIST_5023	-1.310158	3.935439	0.000638	0.023633	*N/A*
ZBIST_0460	-1.272329	4.349126	1.77E-05	0.002917	*YMR244W*
ZBIST_5106	-1.239531	5.754167	0.001738	0.045633	*FLO9*
ZBIST_4159	-1.190152	4.142995	8.38E-05	0.007517	*PHO84*
ZBIST_2058	-1.170935	5.869702	4.38E-06	0.001201	*N/A*
ZBIST_1804	-1.104149	3.886852	0.000478	0.019652	*ECM13*
ZBIST_4949	-1.096918	3.546996	0.000437	0.018501	*YLL058W*
ZBIST_1548	-1.09359	7.448134	7.52E-07	0.000251	*TYE7*
ZBIST_1422	-1.084702	6.401513	1.22E-05	0.002319	*TDA1*
ZBIST_1685	-0.992336	5.529813	0.000234	0.013376	*TPO1*
ZBIST_4879	-0.98178	3.883733	0.000423	0.018298	*MRPL23*
ZBIST_2308	-0.934719	7.816854	9.39E-06	0.002107	*YJL045W*
ZBIST_1185	-0.919892	5.629663	5.4E-05	0.005795	*PEX21*
ZBIST_0761	-0.918171	8.937998	0.000356	0.016051	*CWP1*
ZBIST_4547	-0.90965	7.1162	0.000153	0.010192	*SKS1*
ZBIST_0027	-0.886174	7.347084	8.7E-06	0.002045	*NCE103*
ZBIST_3315	-0.834658	4.331684	0.001186	0.034646	*PPM1*
ZBIST_0029	-0.831651	7.127222	1.14E-05	0.002319	*SGA1*
ZBIST_0957	-0.82429	4.262432	0.001403	0.038687	*GAL7*
ZBIST_2218	-0.817296	9.519184	2.41E-05	0.003715	*KAP123*
ZBIST_1394	-0.803572	6.559423	0.000133	0.009341	*STP3*
ZBIST_4152	-0.803144	4.891417	0.000537	0.02104	*RPM2*
ZBIST_1069	-0.797099	8.260316	0.000354	0.016051	*SHH4*
ZBIST_0039	-0.789851	4.594112	0.001993	0.04865	*N/A*
ZBIST_4518	-0.7897	10.01796	0.000145	0.00992	*MET5*
ZBIST_1711	-0.770768	7.868875	2.79E-05	0.004057	*ACO1*
ZBIST_3347	-0.7505	7.443105	6.69E-05	0.006519	*CLU1*
ZBIST_4345	-0.749022	4.490567	0.001257	0.036081	*N/A*
ZBIST_1471	-0.742067	8.546849	0.000134	0.009341	*MDN1*
ZBIST_0055	-0.740137	7.092795	0.000132	0.009341	*GCV2*
ZBIST_1359	-0.738042	4.886553	0.001851	0.04699	*DBP7*
ZBIST_5037	-0.730073	5.078289	0.001641	0.043776	*N/A*
ZBIST_0936	-0.726868	6.970738	4.59E-05	0.005268	*GCV1*
ZBIST_4702	-0.725025	6.257481	0.000105	0.008361	*UTP20*
ZBIST_3766	-0.72494	6.831222	5.39E-05	0.005795	*ACO1*
ZBIST_1164	-0.724331	8.522606	7.34E-05	0.006837	*ILV3*
ZBIST_3642	-0.719353	5.446743	0.000601	0.022808	*SWS2*
ZBIST_0397	-0.718796	6.887659	0.000254	0.013606	*N/A*
ZBIST_3001	-0.708622	9.439257	0.000258	0.013606	*FPR3*
ZBIST_0026	-0.702354	5.50702	0.000564	0.021698	*XBP1*
ZBIST_1148	-0.693706	9.743493	0.000247	0.013531	*LYS4*
ZBIST_1684	-0.690151	5.763054	0.00127	0.036157	*TPO1*
ZBIST_1883	-0.685681	10.62679	0.000626	0.023575	*GUA1*
ZBIST_3654	-0.677551	7.758127	0.000132	0.009341	*N/A*

ZBIST_3170	-0.666471	9.358127	0.000701	0.024722	*TPO1*
ZBIST_1815	-0.665541	9.324729	0.000438	0.018501	*CPA2*
ZBIST_1991	-0.660934	6.987743	0.000346	0.016051	*ARX1*
ZBIST_2019	-0.658772	8.962631	0.000849	0.028322	*URA2*
ZBIST_1562	-0.652377	7.237595	0.000367	0.016304	*CIR2*
ZBIST_0428	-0.651012	6.121197	0.000656	0.023826	*MET13*
ZBIST_2506	-0.648278	9.437488	0.000976	0.031477	*N/A*
ZBIST_3751	-0.647538	6.055744	0.000351	0.016051	*MRP13*
ZBIST_1323	-0.636426	5.260239	0.00183	0.04699	*DHR2*
ZBIST_3800	-0.631899	7.93665	0.000488	0.019905	*MDM38*
ZBIST_1793	-0.631475	5.762242	0.00081	0.027206	*RSM26*
ZBIST_4657	-0.628015	6.353684	0.000409	0.018006	*MEF1*
ZBIST_5089	-0.625066	7.936915	0.001043	0.032513	*YJL218W*
ZBIST_1629	-0.62465	6.352916	0.000564	0.021698	*YHL008C*
ZBIST_1593	-0.624037	6.193681	0.000647	0.023658	*BDH1*
ZBIST_3576	-0.619107	5.893617	0.001046	0.032513	*N/A*
ZBIST_2900	-0.618512	9.93386	0.00068	0.024314	*DBP2*
ZBIST_0152	-0.615632	6.064982	0.001044	0.032513	*MOT1*
ZBIST_3294	-0.599634	6.258729	0.001679	0.044553	*YML6*
ZBIST_0960	-0.58869	6.711126	0.001201	0.03488	*MAK5*
ZBIST_1211	-0.587005	5.980751	0.001279	0.036157	*WSC4*
ZBIST_0399	-0.585196	5.680414	0.001933	0.047825	*OXA1*

(1) logFC - logarithm base 2 of fold change.
(2) logCPM - logarithm base 2 of counts per million.
(3) FDR - false discovery rate

Supplementary Table S5. Genes found to be differently transcribed in the *Z. bailii* IST302 *Zbhaa1Δ* deletion mutant strain upon sudden exposure to acetic acid stress compared with unstressed cells.

Z. bailii ORF	logFC [(1)]	logCPM [(2)]	PValue	FDR [(3)]	*S. cerevisiae* Homologue
**Upregulated**					
ZBIST_0204	1.476705	4.153203	1.9E-07	3.86E-05	*N/A*
**Downregulated**					
ZBIST_5080	-7.986856	2.263865	1E-21	1.63E-18	*N/A*
ZBIST_3442	-3.263627	3.121905	5.2E-16	4.23E-13	*HSP26*
ZBIST_0079	-2.870891	3.168154	1.42E-14	8.64E-12	*HSP26*
ZBIST_2178	-2.478658	3.336569	6.06E-13	3.29E-10	*RSB1*
ZBIST_5053	-2.469447	2.664852	5E-10	1.75E-07	*HNM1*
ZBIST_0509	-2.458453	6.524974	2.59E-20	3.16E-17	*YGP1*
ZBIST_5112	-2.331458	5.98819	4.6E-29	2.25E-25	*ARR3*
ZBIST_5070	-2.18158	6.221575	1.11E-16	1.09E-13	*N/A*
ZBIST_5079	-2.086189	5.950721	4.45E-26	1.09E-22	*GCN4*
ZBIST_0039	-1.969667	5.016251	6.79E-11	3.02E-08	*N/A*
ZBIST_0882	-1.787714	4.372702	1.42E-09	4.61E-07	*YPS1*
ZBIST_0021	-1.712602	3.564914	2.8E-09	7.59E-07	*MMF1*
ZBIST_3849	-1.686646	1.643488	0.000338	0.031168	*PES4*
ZBIST_0876	-1.668071	8.781414	1.13E-10	4.6E-08	*N/A*
ZBIST_3445	-1.654336	4.503259	6.24E-07	0.000113	*FDH1*
ZBIST_4572	-1.541931	7.712364	1.24E-05	0.00163	*N/A*
ZBIST_3873	-1.53614	6.883699	2.41E-15	1.68E-12	*N/A*
ZBIST_5088	-1.524608	3.906084	2.99E-08	7.31E-06	*LDH1*
ZBIST_4502	-1.505274	2.412688	5.12E-05	0.006255	*N/A*
ZBIST_2471	-1.404556	3.748578	2.34E-06	0.000346	*ICL1*
ZBIST_4629	-1.257344	5.806797	1.8E-09	5.51E-07	*GRE2*
ZBIST_2207	-1.243973	6.261368	1.28E-10	4.82E-08	*SSA3*
ZBIST_0952	-1.226176	7.454023	1.09E-11	5.31E-09	*N/A*
ZBIST_4116	-1.223841	5.253957	3.69E-08	8.2E-06	*HSP42*
ZBIST_3490	-1.189855	6.005717	1.83E-08	4.7E-06	*N/A*
ZBIST_5024	-1.169729	8.349976	0.000247	0.024618	*N/A*
ZBIST_5037	-1.169383	4.712592	6.66E-08	1.41E-05	*N/A*
ZBIST_0459	-1.155203	3.768179	0.00013	0.015445	*HSP30*
ZBIST_1688	-1.139639	6.860312	2.51E-09	7.22E-07	*HSP104*
ZBIST_2833	-1.081446	9.795639	0.000349	0.031534	*TPI1*
ZBIST_0421	-1.048565	6.037377	3.19E-08	7.41E-06	*N/A*
ZBIST_2933	-1.014917	7.809033	6.7E-07	0.000117	*N/A*
ZBIST_4738	-0.997875	3.54495	0.000324	0.030438	*YAT1*
ZBIST_2514	-0.994742	8.350185	4.81E-06	0.000671	*PLB1*
ZBIST_0481	-0.98036	5.805412	5.68E-07	0.000107	*HRK1*
ZBIST_0124	-0.970684	6.87176	1.74E-06	0.000273	*N/A*
ZBIST_4323	-0.934356	5.886604	3.03E-06	0.000436	*GLK1*
ZBIST_4280	-0.927754	10.10438	4.93E-07	9.64E-05	*GOR1*
ZBIST_0507	-0.921902	5.768434	8.15E-07	0.000137	*YHR138C*
ZBIST_1394	-0.881956	5.540432	0.000154	0.017942	*STP3*
ZBIST_2094	-0.875952	4.180935	0.000198	0.021059	*TDA10*
ZBIST_4760	-0.870058	6.255107	2.38E-05	0.003062	*HSP78*
ZBIST_4004	-0.841489	7.296186	8.89E-07	0.000145	*N/A*
ZBIST_4902	-0.839228	8.604444	1.79E-06	0.000274	*MDH1*

ZBIST_2390	-0.82831	4.329719	0.000393	0.03485	*N/A*
ZBIST_0953	-0.812309	8.628359	9.51E-06	0.00129	*YRO2*
ZBIST_4813	-0.803163	5.223046	0.000164	0.018578	*N/A*
ZBIST_2306	-0.76455	8.368161	4.01E-05	0.005021	*MCR1*
ZBIST_3338	-0.760548	5.80843	0.000168	0.018595	*GIS4*
ZBIST_5106	-0.710354	6.061615	0.000471	0.041091	*FLO9*
ZBIST_4164	-0.67082	7.645535	0.000282	0.027512	*MSK1*
ZBIST_2304	-0.667887	9.629546	0.00024	0.024409	*GPM1*
ZBIST_5089	-0.652107	7.780816	0.000195	0.021059	*YJL218W*
ZBIST_1061	-0.631945	6.954371	0.000203	0.021135	*N/A*
ZBIST_3423	-0.614624	10.10594	0.000322	0.030438	*PGK1*

(1) logFC - logarithm base 2 of fold change.
(2) logCPM - logarithm base 2 of counts per million.
(3) FDR - false discovery rate

Supplementary Table S6. Genes found to have increased mRNA levels (upregulated) in the *Z. bailii* IST302 *Zbhaa1*Δ deletion mutant strain upon sudden exposure to copper stress compared with unstressed cells.

Z. bailii ORF	logFC (1)	logCPM (2)	PValue	FDR (3)	S. cerevisiae Homologue
ZBIST_2394	4.735609	3.441181	3.68E-08	6.72E-06	*N/A*
ZBIST_3442	4.537616	6.742882	0.00203	0.01206	*HSP26*
ZBIST_2207	4.497837	10.55207	4.32E-08	7.15E-06	*SSA3*
ZBIST_4116	3.833779	8.497837	2.6E-11	4.28E-08	*HSP42*
ZBIST_2632	3.793412	7.795285	2.46E-12	6.06E-09	*BUL1*
ZBIST_1688	3.396051	9.570637	6.33E-11	5.72E-08	*HSP104*
ZBIST_0755	3.232027	8.638836	4.99E-10	2.46E-07	*BTN2*
ZBIST_2935	3.152912	7.329704	1.21E-10	7.89E-08	*YNL134C*
ZBIST_4760	3.123977	8.028222	1.65E-10	9.07E-08	*HSP78*
ZBIST_3334	3.099709	3.090993	4.65E-06	0.000144	*HSP26*
ZBIST_1117	2.913124	8.002648	5.21E-11	5.72E-08	*N/A*
ZBIST_0711	2.805683	11.78763	7.6E-10	3.12E-07	*OYE2*
ZBIST_3403	2.734361	7.828423	1.28E-10	7.89E-08	*SIS1*
ZBIST_1918	2.67154	10.25961	4.62E-09	1.56E-06	*N/A*
ZBIST_0079	2.630277	5.010635	9.04E-09	2.25E-06	*HSP26*
ZBIST_1232	2.435391	6.740498	9.11E-09	2.25E-06	*N/A*
ZBIST_4078	2.40922	7.50622	4.84E-09	1.56E-06	*OYE2*
ZBIST_0817	2.180154	7.770947	1.44E-07	1.62E-05	*N/A*
ZBIST_2864	2.154132	10.75971	1.82E-08	3.46E-06	*N/A*
ZBIST_5024	2.127518	8.782049	5.38E-09	1.56E-06	*N/A*
ZBIST_4542	2.115575	9.680142	5.1E-09	1.56E-06	*UBI4*
ZBIST_2242	2.094771	5.933502	1.17E-07	1.51E-05	*N/A*
ZBIST_0127	2.032733	4.043427	2.71E-05	0.000441	*MUM2*
ZBIST_2860	2.005756	2.592707	0.000354	0.003079	*N/A*
ZBIST_2575	1.997149	10.56222	3.51E-07	2.88E-05	*HSP12*
ZBIST_2002	1.98423	9.547046	1.67E-08	3.46E-06	*YDR109C*
ZBIST_1116	1.956792	10.50766	3.77E-07	3.01E-05	*AHA1*
ZBIST_1072	1.947218	2.006689	0.006934	0.029608	*PUS5*
ZBIST_3841	1.916712	5.951889	3.78E-06	0.000128	*N/A*
ZBIST_1181	1.915002	2.620495	0.005524	0.02518	*N/A*
ZBIST_0204	1.909467	5.008929	1.44E-06	7.02E-05	*N/A*
ZBIST_1951	1.908882	2.22794	0.004322	0.021169	*TPP1*
ZBIST_2663	1.848951	8.393176	4.49E-08	7.15E-06	*CPR6*
ZBIST_1495	1.840406	5.773221	2.06E-06	8.54E-05	*N/A*
ZBIST_4758	1.836568	8.561062	1.35E-07	1.55E-05	*STI1*
ZBIST_1475	1.83255	5.806349	8.37E-07	4.86E-05	*N/A*
ZBIST_3175	1.81305	7.889067	1.28E-06	6.33E-05	*FLR1*
ZBIST_5055	1.7869	1.783326	0.00883	0.036109	*N/A*
ZBIST_2634	1.765765	11.03445	1.5E-06	7.24E-05	*N/A*
ZBIST_2658	1.756144	4.392325	2.77E-05	0.000445	*NUS1*
ZBIST_2103	1.753879	3.986236	2.14E-05	0.000379	*REC102*
ZBIST_4640	1.750958	9.426502	3.82E-08	6.74E-06	*YML131W*
ZBIST_4140	1.747704	10.14622	4.36E-07	3.21E-05	*ERO1*
ZBIST_0065	1.742768	13.55171	1.24E-06	6.33E-05	*HSP82*
ZBIST_0577	1.735395	3.703421	0.001284	0.008252	*OYE2*
ZBIST_4946	1.72891	9.013596	1.01E-07	1.38E-05	*N/A*
ZBIST_1233	1.722248	4.213365	0.00013	0.001394	*N/A*

ZBIST_0460	1.715782	4.663749	0.004269	0.021052	*YMR244W*
ZBIST_2184	1.715101	8.192608	1.23E-07	1.51E-05	*N/A*
ZBIST_1119	1.708425	5.010376	1.98E-06	8.49E-05	*ADR1*
ZBIST_1003	1.702689	10.03546	2.82E-06	0.000111	*HCH1*
ZBIST_3678	1.672121	7.06437	2.74E-07	2.42E-05	*FES1*
ZBIST_1666	1.66931	5.59481	3.63E-06	0.000128	*BYE1*
ZBIST_1305	1.668094	6.064781	3.24E-06	0.000124	*PCL5*
ZBIST_4767	1.654308	5.352608	5.62E-06	0.000164	*JLP1*
ZBIST_0459	1.653936	4.391759	3.08E-05	0.000481	*HSP30*
ZBIST_3637	1.650548	5.83325	0.000266	0.002445	*APJ1*
ZBIST_3176	1.650545	4.23245	3.19E-05	0.000492	*FLR1*
ZBIST_4186	1.649317	3.76751	6.58E-05	0.000822	*MTQ1*
ZBIST_3458	1.648551	2.523064	0.011746	0.044946	*N/A*
ZBIST_0694	1.633097	4.383257	6.04E-05	0.00077	*DNA2*
ZBIST_2390	1.626307	5.265923	3.63E-06	0.000128	*N/A*
ZBIST_4106	1.608251	6.89913	3.59E-06	0.000128	*CDC37*
ZBIST_4472	1.607641	5.671176	3.03E-05	0.000478	*ATG9*
ZBIST_0230	1.5943	8.382521	1.06E-07	1.41E-05	*CYR1*
ZBIST_3646	1.579006	4.115607	2.5E-05	0.000414	*MCH2*
ZBIST_1476	1.5649	14.37514	5.97E-05	0.000763	*AHP1*
ZBIST_1073	1.557004	6.096352	2.04E-07	2.1E-05	*SCC2*
ZBIST_0452	1.552373	6.337516	6.58E-06	0.000175	*GAD1*
ZBIST_0843	1.552229	5.730943	4.07E-06	0.000134	*N/A*
ZBIST_0988	1.524586	3.077792	0.001652	0.010167	*PAC11*
ZBIST_1692	1.521454	13.61661	1.13E-05	0.000251	*SSA1*
ZBIST_4493	1.516639	6.33768	7.37E-07	4.49E-05	*MCA1*
ZBIST_4131	1.510053	4.365167	0.000138	0.001447	*YPL088W*
ZBIST_2751	1.506605	6.116743	7.32E-07	4.49E-05	*N/A*
ZBIST_3260	1.491718	3.058978	0.000957	0.006625	*GAC1*
ZBIST_0142	1.486281	5.336822	1.59E-05	0.000319	*YPC1*
ZBIST_1150	1.482558	5.025415	1.39E-05	0.000293	*PRP42*
ZBIST_2033	1.475516	3.966686	0.00015	0.001541	*N/A*
ZBIST_0634	1.475443	9.555684	5.88E-06	0.000168	*MBF1*
ZBIST_3835	1.471328	9.977299	2.21E-06	9.08E-05	*UBP6*
ZBIST_1108	1.469838	5.741905	5.44E-06	0.000161	*UME6*
ZBIST_1180	1.469425	2.401819	0.005929	0.026437	*N/A*
ZBIST_4185	1.45297	10.37058	8.71E-07	5E-05	*YDJ1*
ZBIST_1997	1.446515	4.300905	0.000128	0.001379	*ISC10*
ZBIST_4379	1.444531	6.770743	7.77E-06	0.000195	*N/A*
ZBIST_1704	1.442053	4.868824	9.17E-06	0.000218	*SUA7*
ZBIST_1446	1.441756	3.138624	0.001887	0.01136	*FKS3*
ZBIST_4332	1.440385	5.705745	0.000117	0.001272	*SPS1*
ZBIST_5063	1.432575	7.113773	5.26E-06	0.000158	*PDC1*
ZBIST_0500	1.424096	8.761389	1.13E-06	5.96E-05	*N/A*
ZBIST_4708	1.421512	7.125672	1.02E-06	5.56E-05	*NTH2*
ZBIST_4305	1.42139	8.032455	0.000178	0.001792	*FRM2*
ZBIST_1891	1.413	7.300234	3.41E-07	2.88E-05	*GRE3*
ZBIST_1670	1.405639	6.312882	3.29E-06	0.000125	*HSE1*
ZBIST_3965	1.404185	6.615267	2.47E-05	0.000413	*AHC1*
ZBIST_0764	1.403883	6.280386	1.03E-06	5.56E-05	*N/A*

ZBIST_2054	1.403227	5.026433	1.9E-05	0.000353	*BTS1*
ZBIST_1817	1.402183	4.162136	0.000169	0.001714	*CCP1*
ZBIST_4636	1.396004	10.32645	5.44E-05	0.000714	*N/A*
ZBIST_1805	1.391039	2.633413	0.010111	0.040216	*N/A*
ZBIST_0375	1.386236	5.725822	1.69E-06	7.79E-05	*UBC13*
ZBIST_5069	1.380892	4.327524	9.56E-05	0.001092	*IMA2*
ZBIST_0891	1.372781	4.219426	0.000217	0.002066	*SLD2*
ZBIST_0650	1.368679	6.509912	1.84E-06	8.35E-05	*UBX5*
ZBIST_0264	1.363887	7.19578	2.85E-06	0.000112	*SNX4*
ZBIST_2108	1.362448	6.531756	3.81E-05	0.000559	*COS111*
ZBIST_1978	1.359489	7.684441	2.57E-06	0.000103	*N/A*
ZBIST_1936	1.358765	4.931003	8.34E-05	0.000973	*PAH1*
ZBIST_0877	1.349623	10.69806	4.66E-06	0.000144	*SBA1*
ZBIST_2729	1.349586	9.733275	7.77E-07	4.68E-05	*PEP4*
ZBIST_2537	1.342507	6.132603	1.12E-05	0.000251	*N/A*
ZBIST_4925	1.341111	6.316044	4.48E-06	0.000143	*YPL088W*
ZBIST_2791	1.323654	3.362079	0.003627	0.018707	*LGE1*
ZBIST_2107	1.323048	6.452252	0.000242	0.002267	*FRE3*
ZBIST_2787	1.322455	2.971705	0.007528	0.03168	*N/A*
ZBIST_4652	1.316835	5.956081	6.15E-06	0.000171	*OTU1*
ZBIST_3336	1.307014	6.789588	0.000395	0.003317	*YAP1*
ZBIST_4196	1.306751	3.839731	0.000881	0.006275	*VAC7*
ZBIST_3014	1.305183	3.332671	0.002883	0.015708	*N/A*
ZBIST_2309	1.303938	6.256014	4.84E-06	0.000147	*NPL4*
ZBIST_3199	1.300126	3.29903	0.00309	0.016615	*HAP2*
ZBIST_0712	1.299036	4.511308	0.000291	0.002612	*OYE2*
ZBIST_1789	1.297468	5.328571	3.22E-05	0.000495	*YJR096W*
ZBIST_2434	1.295249	7.78691	1.51E-06	7.24E-05	*YDL124W*
ZBIST_5084	1.292767	5.210335	0.000137	0.001436	*DUR1,2*
ZBIST_2788	1.286729	3.974309	0.002857	0.01558	*N/A*
ZBIST_1619	1.286628	2.876789	0.012504	0.047064	*N/A*
ZBIST_2340	1.285568	9.652606	1.98E-06	8.49E-05	*ZWF1*
ZBIST_3697	1.28504	4.749232	0.000112	0.001238	*N/A*
ZBIST_3256	1.284666	5.563958	1.04E-05	0.000236	*YLR278C*
ZBIST_2486	1.28449	10.44328	2.57E-05	0.000422	*PRB1*
ZBIST_2971	1.280368	4.371121	0.000217	0.002066	*N/A*
ZBIST_2609	1.279749	7.864173	9.09E-07	5.16E-05	*RPN11*
ZBIST_1699	1.270303	2.668113	0.012271	0.046448	*N/A*
ZBIST_4777	1.264556	7.989449	6.27E-06	0.000171	*PCM1*
ZBIST_3423	1.263141	11.64225	1.98E-05	0.000366	*PGK1*
ZBIST_2752	1.258318	4.099779	0.002571	0.014522	*N/A*
ZBIST_4751	1.25697	8.701161	1.12E-06	5.93E-05	*N/A*
ZBIST_2191	1.252929	8.387995	1.28E-06	6.33E-05	*ROG1*
ZBIST_3584	1.24204	4.492477	0.000425	0.003497	*N/A*
ZBIST_4541	1.240726	6.911572	2.27E-06	9.17E-05	*ECM29*
ZBIST_2985	1.238917	3.943014	0.000861	0.006186	*FLR1*
ZBIST_3283	1.230826	5.11188	3.61E-05	0.00054	*RAD52*
ZBIST_0087	1.228989	6.138748	1.87E-05	0.000351	*N/A*
ZBIST_4570	1.226286	5.347086	0.000829	0.005998	*SDS3*
ZBIST_0569	1.223297	6.459152	4.56E-06	0.000144	*DOA1*

ZBIST_0454	1.220134	7.577184	2.12E-05	0.000379	*N/A*
ZBIST_2142	1.215675	10.10385	1.66E-05	0.000325	*RPT5*
ZBIST_0267	1.214353	10.70896	6.08E-05	0.000771	*KAR2*
ZBIST_4339	1.212713	6.726943	4.44E-06	0.000143	*APA1*
ZBIST_5004	1.20861	10.30016	4.65E-05	0.000641	*DSK2*
ZBIST_1183	1.206055	5.355433	4.22E-05	0.000597	*KEL1*
ZBIST_3052	1.204935	3.018179	0.006153	0.027142	*N/A*
ZBIST_4108	1.203111	2.850921	0.006014	0.026671	*STB3*
ZBIST_4198	1.201236	5.309339	3.84E-05	0.000561	*N/A*
ZBIST_3181	1.198382	3.86134	0.000631	0.004834	*HFM1*
ZBIST_0507	1.198249	7.008277	0.000112	0.001238	*YHR138C*
ZBIST_3859	1.196542	5.21854	6.3E-05	0.000793	*N/A*
ZBIST_3431	1.19592	5.468244	4.91E-05	0.000665	*GLC3*
ZBIST_4846	1.19528	3.775312	0.00205	0.012133	*ATG13*
ZBIST_0049	1.195158	6.498732	2.5E-05	0.000414	*POX1*
ZBIST_4205	1.193766	6.056358	7.61E-06	0.000193	*PFK26*
ZBIST_3182	1.193506	5.063442	0.000201	0.001951	*N/A*
ZBIST_3355	1.190595	4.343592	0.000191	0.001872	*N/A*
ZBIST_0114	1.1869	5.58621	2.67E-05	0.000434	*N/A*
ZBIST_1608	1.185624	5.526653	4.02E-05	0.00058	*SIA1*
ZBIST_0648	1.185276	7.001119	4.04E-06	0.000134	*KSP1*
ZBIST_0056	1.184813	6.857343	1.96E-05	0.000364	*N/A*
ZBIST_0362	1.181762	4.762845	0.000219	0.002078	*N/A*
ZBIST_4903	1.181172	6.032266	1.42E-05	0.000294	*SRX1*
ZBIST_1322	1.179541	6.081734	1.82E-05	0.000343	*STL1*
ZBIST_3337	1.178453	4.719807	0.000166	0.001692	*SIP1*
ZBIST_3582	1.177949	6.049472	4.12E-05	0.000586	*N/A*
ZBIST_4876	1.174896	6.113995	0.000322	0.002856	*N/A*
ZBIST_3729	1.173835	3.95561	0.001021	0.006943	*N/A*
ZBIST_5008	1.165315	6.58597	8.57E-06	0.000206	*SAM4*
ZBIST_0412	1.164152	7.922005	7.77E-06	0.000195	*YGL114W*
ZBIST_1680	1.162192	4.004882	0.001237	0.008003	*YJU2*
ZBIST_3272	1.160149	5.145334	2.87E-05	0.000459	*SPP41*
ZBIST_3486	1.158008	5.537106	4.68E-05	0.000642	*DDI1*
ZBIST_1368	1.158001	4.359342	0.0009	0.006373	*STU2*
ZBIST_4970	1.147932	5.41582	6.33E-05	0.000796	*MPE1*
ZBIST_2573	1.145123	6.780664	5.37E-06	0.00016	*MDJ1*
ZBIST_0486	1.141895	7.696326	6.07E-06	0.00017	*RPN8*
ZBIST_1661	1.141628	7.77617	3.78E-06	0.000128	*VPS1*
ZBIST_2290	1.138336	4.352519	0.00062	0.004764	*RAD14*
ZBIST_4845	1.138305	7.863744	4.63E-05	0.00064	*GDB1*
ZBIST_1683	1.136788	6.710411	1.35E-05	0.000288	*N/A*
ZBIST_4919	1.132252	6.487513	5.12E-05	0.000685	*GTT1*
ZBIST_2069	1.132017	9.604032	2.09E-05	0.000379	*N/A*
ZBIST_2083	1.12826	6.007552	6.94E-05	0.000844	*N/A*
ZBIST_4031	1.127286	9.039378	1.33E-05	0.000283	*APE2*
ZBIST_0956	1.127183	5.666557	9.03E-05	0.001044	*GAL10*
ZBIST_0699	1.127125	5.268499	0.000248	0.002312	*MTG2*
ZBIST_4107	1.122534	6.365548	2.11E-05	0.000379	*N/A*
ZBIST_1285	1.122333	6.762762	3.36E-05	0.000508	*YDR306C*

ZBIST_0962	1.118139	5.500752	0.000129	0.001388	*N/A*
ZBIST_3801	1.117283	4.078986	0.001615	0.009979	*YAP5*
ZBIST_2844	1.1169	6.198641	6.81E-05	0.00084	*CDC34*
ZBIST_1507	1.116212	3.240276	0.005261	0.024361	*PEX15*
ZBIST_0091	1.114098	4.099397	0.003403	0.017832	*HOT1*
ZBIST_1823	1.109452	9.426093	3.82E-05	0.000559	*HSP150*
ZBIST_0485	1.109194	5.20705	0.000281	0.002544	*GPN3*
ZBIST_4534	1.108018	7.029402	9.67E-06	0.000226	*YML082W*
ZBIST_3444	1.10694	5.863529	1.57E-05	0.000317	*ROX3*
ZBIST_3817	1.105527	6.387537	1.74E-05	0.000332	*CAT5*
ZBIST_2608	1.103639	5.911858	0.000211	0.002032	*YPI1*
ZBIST_0944	1.100737	4.218058	0.000938	0.006538	*N/A*
ZBIST_3270	1.096293	6.179137	9.99E-05	0.001139	*PDR8*
ZBIST_1995	1.09391	3.626032	0.011625	0.044603	*DMC1*
ZBIST_4041	1.09155	8.784983	0.000106	0.001192	*INM2*
ZBIST_0357	1.090669	10.30564	2.37E-05	0.000408	*YIM1*
ZBIST_2423	1.090586	5.592835	6.3E-05	0.000793	*CDC53*
ZBIST_1556	1.089337	5.496913	7E-05	0.000845	*SOG2*
ZBIST_2065	1.088423	5.404241	5.4E-05	0.000711	*N/A*
ZBIST_2338	1.087847	7.067163	6.32E-06	0.000171	*ATG2*
ZBIST_4550	1.08778	6.196545	2.12E-05	0.000379	*SUV3*
ZBIST_4448	1.086365	4.423066	0.000906	0.006378	*N/A*
ZBIST_4973	1.080349	4.107527	0.001731	0.010547	*PEX12*
ZBIST_4420	1.077925	8.520287	1.16E-05	0.000257	*RPN3*
ZBIST_3791	1.076942	3.575547	0.005569	0.02531	*IST3*
ZBIST_0489	1.07686	8.116464	1.17E-05	0.000257	*RPT4*
ZBIST_0564	1.074802	7.256318	6.32E-06	0.000171	*MOG1*
ZBIST_1952	1.070525	8.66532	1.41E-05	0.000293	*RPN6*
ZBIST_3157	1.070061	7.263754	1.54E-05	0.000313	*ARE2*
ZBIST_3367	1.067938	3.040889	0.011171	0.043317	*N/A*
ZBIST_3432	1.066734	5.960729	2.62E-05	0.000428	*UBC8*
ZBIST_4772	1.066101	5.557969	5.5E-05	0.000719	*MDL2*
ZBIST_3063	1.065862	5.425103	0.000137	0.001436	*PXA2*
ZBIST_4150	1.065362	7.261306	1.88E-05	0.000351	*MSC1*
ZBIST_4566	1.065322	4.707913	0.000303	0.002721	*CAB2*
ZBIST_4875	1.065003	4.890746	0.000887	0.006303	*PDR15*
ZBIST_3598	1.063415	5.122393	6.83E-05	0.000841	*N/A*
ZBIST_0281	1.063016	11.09958	4.04E-05	0.000581	*SSE1*
ZBIST_0451	1.061713	11.05682	0.0007	0.005264	*N/A*
ZBIST_4731	1.06036	3.600235	0.006883	0.029488	*ERP3*
ZBIST_0197	1.049057	4.768983	0.001621	0.00999	*MID2*
ZBIST_0189	1.046522	6.500741	0.000181	0.001812	*ERV1*
ZBIST_0090	1.042073	9.796364	0.000248	0.002312	*DDR48*
ZBIST_3328	1.034221	7.107578	4.48E-05	0.000623	*RPN9*
ZBIST_0381	1.034122	5.067324	0.001086	0.007295	*RAD4*
ZBIST_2302	1.032868	6.805768	0.000142	0.00148	*MHP1*
ZBIST_0626	1.032835	5.353757	0.000207	0.002005	*TGL3*
ZBIST_0046	1.031596	5.890801	0.000724	0.005401	*AYR1*
ZBIST_2702	1.029577	7.984296	0.00013	0.001396	*WTM2*
ZBIST_2768	1.028129	4.973052	0.004231	0.020999	*SSN3*

ZBIST_4890	1.027394	5.710165	0.000163	0.001671	*SHE9*
ZBIST_3830	1.025942	5.165765	0.000485	0.003922	*N/A*
ZBIST_3891	1.025886	3.373336	0.013141	0.04899	*PRP18*
ZBIST_4899	1.025036	5.248077	0.000136	0.001436	*N/A*
ZBIST_0958	1.022628	7.187338	0.000131	0.001402	*RPT2*
ZBIST_2628	1.021657	10.18311	3.67E-05	0.000545	*SSC1*
ZBIST_2674	1.014535	4.327088	0.001692	0.010376	*PTP2*
ZBIST_3914	1.013292	6.668583	5E-05	0.000673	*RFA1*
ZBIST_0576	1.011021	4.179004	0.005052	0.023773	*FLR1*
ZBIST_1973	1.008021	5.429654	0.005194	0.024251	*N/A*
ZBIST_4264	1.007063	4.548944	0.002695	0.01498	*UFD1*
ZBIST_3967	1.00664	6.715181	0.000182	0.001812	*MAM3*
ZBIST_4923	1.00634	5.602741	0.000134	0.001421	*YPL088W*
ZBIST_3633	1.005674	8.686517	3.53E-05	0.00053	*PRE1*
ZBIST_1895	1.00532	4.877416	0.000481	0.003903	*KIC1*
ZBIST_0943	1.004866	6.173568	0.002818	0.015456	*ADH1*
ZBIST_3566	1.004587	3.35119	0.010907	0.04249	*GIP2*
ZBIST_1622	1.002605	7.511763	1.28E-05	0.000275	*YEA6*
ZBIST_0081	1.000765	6.902877	2.29E-05	0.000398	*CET1*
ZBIST_2865	1.000375	5.777794	4.67E-05	0.000642	*STU1*
ZBIST_0907	0.999901	4.895078	0.000914	0.006427	*TCO89*
ZBIST_3806	0.99861	4.356094	0.000933	0.006513	*ARO10*
ZBIST_2473	0.9979	7.838835	3.81E-05	0.000559	*N/A*
ZBIST_4223	0.99512	8.104422	0.000688	0.005193	*SHP1*
ZBIST_4980	0.994264	6.460523	6.96E-05	0.000844	*UBC7*
ZBIST_0320	0.993592	7.493613	2.24E-05	0.000392	*SMF2*
ZBIST_0502	0.99294	4.692785	0.004744	0.022736	*YPL088W*
ZBIST_2280	0.981474	6.722636	0.000226	0.002146	*N/A*
ZBIST_4795	0.978728	5.334658	0.000751	0.005573	*MSN4*
ZBIST_0971	0.977088	3.96282	0.010095	0.040186	*SPP381*
ZBIST_3043	0.976549	7.014565	3.03E-05	0.000478	*YKT6*
ZBIST_1085	0.976535	8.280456	2.39E-05	0.000409	*CCT6*
ZBIST_4184	0.973408	4.12423	0.002832	0.015514	*QDR2*
ZBIST_2445	0.973315	8.192977	3.82E-05	0.000559	*RPN2*
ZBIST_2158	0.972644	6.864717	6.68E-05	0.000831	*YPS1*
ZBIST_2289	0.972394	6.790971	2.46E-05	0.000413	*VPS27*
ZBIST_1684	0.972056	5.958256	0.000362	0.003113	*TPO1*
ZBIST_2431	0.971596	11.87561	0.000238	0.002232	*CDC48*
ZBIST_1043	0.971201	7.880966	4.53E-05	0.000628	*N/A*
ZBIST_3702	0.968965	5.457538	0.00011	0.001226	*MED1*
ZBIST_4370	0.967627	7.794746	3.15E-05	0.000488	*RPT6*
ZBIST_4146	0.967215	9.949836	5.2E-05	0.00069	*PRE8*
ZBIST_4715	0.967007	5.219621	0.000231	0.002177	*RAD57*
ZBIST_2833	0.961232	10.63748	0.000199	0.001936	*TPI1*
ZBIST_0214	0.958479	6.123439	4.72E-05	0.000646	*UBR2*
ZBIST_3659	0.956693	4.164258	0.006676	0.028993	*N/A*
ZBIST_3754	0.956634	9.156187	3.93E-05	0.000572	*PRE2*
ZBIST_0126	0.956316	7.867352	0.000146	0.00151	*UBP14*
ZBIST_2512	0.956203	6.4297	4.32E-05	0.000606	*TAF4*
ZBIST_2650	0.952364	4.091195	0.003575	0.018518	*ROG1*

ZBIST_2578	0.949039	10.06809	0.000715	0.00534	*WWM1*
ZBIST_2312	0.947767	7.021424	5.02E-05	0.000674	*UMP1*
ZBIST_1600	0.944654	5.499261	0.001173	0.00767	*N/A*
ZBIST_3760	0.937283	6.411223	0.000184	0.001828	*CTT1*
ZBIST_1000	0.934412	3.754208	0.01163	0.044603	*SEC20*
ZBIST_1270	0.932615	7.244949	0.0001	0.001139	*BBC1*
ZBIST_1139	0.931613	5.698507	0.000182	0.001812	*N/A*
ZBIST_4206	0.93144	4.704585	0.001089	0.007302	*MOB1*
ZBIST_4304	0.929627	6.055613	0.000191	0.001872	*AGP1*
ZBIST_2064	0.927463	6.358255	0.000178	0.001791	*CAF130*
ZBIST_0607	0.926623	5.252611	0.000235	0.002216	*XDJ1*
ZBIST_2848	0.926492	6.663378	5.95E-05	0.000763	*SEC18*
ZBIST_1790	0.925952	6.031141	0.000136	0.001435	*YUH1*
ZBIST_0654	0.921956	6.22236	0.000139	0.001451	*N/A*
ZBIST_0644	0.91379	6.135772	0.00015	0.001546	*ERG7*
ZBIST_1655	0.912482	5.654774	0.00029	0.002612	*MEH1*
ZBIST_4346	0.909768	5.224568	0.000491	0.003957	*N/A*
ZBIST_0895	0.908854	7.438998	8.22E-05	0.000962	*YTA12*
ZBIST_3011	0.906587	6.655902	9.21E-05	0.001062	*DAK1*
ZBIST_1320	0.905148	6.349715	0.00105	0.007069	*ARG7*
ZBIST_0563	0.903303	8.562851	8.68E-05	0.001008	*UBA1*
ZBIST_3464	0.902972	6.862175	7.91E-05	0.000931	*PEP1*
ZBIST_0707	0.899559	12.97099	0.000443	0.00363	*ENO1*
ZBIST_0739	0.895995	7.7185	9.43E-05	0.001082	*ASK10*
ZBIST_4326	0.893451	4.746061	0.001075	0.007229	*MGR1*
ZBIST_4516	0.889332	8.353135	0.005635	0.025454	*RPN10*
ZBIST_1926	0.885161	4.603446	0.00331	0.017436	*HUL5*
ZBIST_3179	0.884643	7.428002	0.000112	0.001238	*RPN12*
ZBIST_3265	0.88456	5.180815	0.000961	0.006643	*N/A*
ZBIST_4217	0.882974	8.726369	0.000356	0.003092	*PUP3*
ZBIST_4814	0.882845	6.359915	0.000593	0.004622	*STR3*
ZBIST_4889	0.878455	9.286278	0.000116	0.001272	*RPT3*
ZBIST_4601	0.878343	6.356421	0.000507	0.004051	*N/A*
ZBIST_0023	0.877349	6.442904	0.000122	0.001324	*RHO1*
ZBIST_2121	0.876587	5.148581	0.00076	0.005622	*N/A*
ZBIST_3259	0.87561	5.358808	0.000506	0.004051	*MCM5*
ZBIST_3651	0.87241	7.353425	0.000166	0.001694	*MKT1*
ZBIST_3722	0.871589	4.649047	0.003451	0.018027	*TFC1*
ZBIST_0627	0.868973	8.294319	0.000253	0.002349	*PRE5*
ZBIST_1433	0.868485	9.46561	0.001115	0.007435	*PRC1*
ZBIST_3613	0.867913	4.524613	0.00694	0.029608	*VID28*
ZBIST_4637	0.865965	3.720275	0.012993	0.048587	*N/A*
ZBIST_4445	0.865526	8.601653	0.000274	0.002498	*N/A*
ZBIST_1653	0.86323	6.058934	0.000321	0.00285	*KTR1*
ZBIST_1176	0.862826	4.187229	0.004616	0.022234	*UGA4*
ZBIST_3575	0.862143	7.146828	0.000513	0.004091	*N/A*
ZBIST_0705	0.861801	8.245053	0.000185	0.00183	*PUP2*
ZBIST_4667	0.861555	7.240359	0.000263	0.002415	*N/A*
ZBIST_2562	0.859814	7.901276	0.000673	0.005093	*SBP1*
ZBIST_1744	0.858749	4.371984	0.00308	0.016578	*MMS21*

ZBIST_0282	0.856747	4.44721	0.005261	0.024361	*N/A*
ZBIST_1693	0.856509	8.241534	0.000321	0.00285	*N/A*
ZBIST_3809	0.854386	6.236571	0.000188	0.001852	*RGA1*
ZBIST_4429	0.852228	4.309046	0.005441	0.024893	*YDR131C*
ZBIST_0051	0.849458	8.173348	0.000246	0.002297	*N/A*
ZBIST_1812	0.848298	5.490001	0.000668	0.00507	*YMR1*
ZBIST_3804	0.848133	4.374858	0.00916	0.037212	*TLG2*
ZBIST_4759	0.84693	7.276096	0.000216	0.002066	*MCH4*
ZBIST_2148	0.846721	8.207484	0.002102	0.012366	*GCY1*
ZBIST_1452	0.842701	6.344399	0.000229	0.002165	*NYV1*
ZBIST_0869	0.842341	5.447617	0.000412	0.00341	*SSH4*
ZBIST_4531	0.840355	5.955915	0.000323	0.002859	*N/A*
ZBIST_3853	0.840332	8.389386	0.000267	0.002447	*ARG4*
ZBIST_3638	0.839949	5.164048	0.000573	0.004498	*N/A*
ZBIST_3131	0.839623	5.430802	0.000408	0.003403	*PPG1*
ZBIST_001	0.839443	7.939045	0.000255	0.002355	*N/A*
ZBIST_3826	0.838779	5.389073	0.001306	0.008384	*N/A*
ZBIST_0827	0.838389	6.067163	0.000254	0.002349	*NUP82*
ZBIST_2050	0.836518	4.51209	0.003263	0.017279	*UBP16*
ZBIST_3796	0.834759	7.72387	0.000272	0.002485	*PRE6*
ZBIST_2631	0.833969	5.934719	0.003112	0.016686	*MCM3*
ZBIST_4985	0.833753	5.558624	0.000388	0.003286	*SOK2*
ZBIST_3932	0.833336	4.264588	0.008239	0.034118	*ATE1*
ZBIST_4246	0.833073	5.447478	0.001241	0.008019	*TEX1*
ZBIST_2111	0.832993	7.477932	0.000399	0.003346	*SKY1*
ZBIST_4097	0.832086	5.71339	0.000942	0.006559	*ACF2*
ZBIST_1347	0.828643	4.942023	0.001039	0.007045	*KHA1*
ZBIST_3440	0.827298	6.054913	0.000581	0.004543	*NPP1*
ZBIST_2382	0.825684	11.83178	0.010675	0.041821	*N/A*
ZBIST_0513	0.825288	6.027935	0.000767	0.005648	*CHS7*
ZBIST_0262	0.824297	6.147349	0.000983	0.006759	*PPR1*
ZBIST_3489	0.82263	5.475346	0.001724	0.010538	*DOA4*
ZBIST_3253	0.821143	3.875686	0.012865	0.048218	*N/A*
ZBIST_0779	0.820859	7.59699	0.000339	0.002978	*HYR1*
ZBIST_3635	0.820393	4.875355	0.003688	0.018944	*N/A*
ZBIST_2516	0.819859	5.417738	0.002517	0.014316	*HRD1*
ZBIST_4972	0.819827	11.9968	0.003348	0.01762	*FBA1*
ZBIST_1737	0.819533	4.396058	0.006504	0.028373	*N/A*
ZBIST_0457	0.819116	7.846124	0.000354	0.003079	*FAA1*
ZBIST_1218	0.817618	7.614214	0.000629	0.004829	*NBP35*
ZBIST_1493	0.814642	5.236712	0.002593	0.014594	*SAN1*
ZBIST_0330	0.813854	5.473907	0.001602	0.009924	*GID8*
ZBIST_4016	0.813286	9.144967	0.000361	0.003106	*RPN1*
ZBIST_1925	0.811697	4.832415	0.002432	0.013958	*GPI10*
ZBIST_1174	0.811679	5.227252	0.001522	0.009496	*NAS6*
ZBIST_0965	0.811049	11.84036	0.002426	0.013938	*ADH1*
ZBIST_0510	0.810293	5.612922	0.00827	0.034217	*N/A*
ZBIST_3436	0.809411	5.738214	0.000708	0.005306	*VAC8*
ZBIST_3933	0.808315	4.62729	0.003871	0.019738	*JAC1*
ZBIST_3671	0.808086	5.182723	0.002719	0.015044	*N/A*

ZBIST_0446	0.80771	4.807718	0.005255	0.024361	*MET8*
ZBIST_0410	0.805655	6.224853	0.00041	0.003406	*N/A*
ZBIST_0839	0.803567	6.828193	0.000426	0.003497	*SNF7*
ZBIST_4317	0.803375	9.801596	0.000414	0.003414	*GRX2*
ZBIST_4747	0.803267	10.43411	0.000414	0.003414	*HSP10*
ZBIST_3622	0.803178	7.305197	0.000517	0.004108	*DSN1*
ZBIST_2648	0.802768	8.168927	0.000482	0.003903	*FRD1*
ZBIST_1979	0.801727	5.218385	0.00111	0.007423	*PFS2*
ZBIST_0253	0.801157	5.967226	0.000711	0.005321	*SSL1*
ZBIST_4421	0.798578	7.709267	0.002605	0.014645	*STE24*
ZBIST_3946	0.797135	8.872404	0.000494	0.003973	*TRP5*
ZBIST_3820	0.796784	7.526549	0.000497	0.003993	*SMC5*
ZBIST_1749	0.793365	8.76568	0.000532	0.004204	*YME1*
ZBIST_2556	0.793311	6.934748	0.000919	0.006447	*RKM1*
ZBIST_1824	0.793288	5.672533	0.006931	0.029608	*HSP150*
ZBIST_0054	0.793066	5.700336	0.001785	0.010826	*SEO1*
ZBIST_1612	0.791282	6.921651	0.001043	0.007049	*RVS167*
ZBIST_3422	0.789893	6.69076	0.000532	0.004204	*ADP1*
ZBIST_3802	0.78899	5.427411	0.002674	0.014915	*GAS5*
ZBIST_4269	0.788386	5.206625	0.002938	0.015937	*LYP1*
ZBIST_2410	0.787803	5.216428	0.005969	0.026591	*NPR1*
ZBIST_4207	0.787774	5.354858	0.000954	0.006618	*ALG11*
ZBIST_2188	0.786781	5.273048	0.001496	0.009382	*SGT1*
ZBIST_1355	0.786072	7.846034	0.00061	0.004712	*GSH1*
ZBIST_0685	0.784003	6.158721	0.000597	0.004637	*CCC2*
ZBIST_0240	0.780103	10.38265	0.000603	0.004683	*PRE3*
ZBIST_2583	0.778034	7.225791	0.000881	0.006275	*BLM10*
ZBIST_1916	0.777992	5.702767	0.000816	0.005943	*MSN5*
ZBIST_0885	0.777304	6.127407	0.001896	0.011401	*KTI12*
ZBIST_1269	0.776869	5.105268	0.003783	0.019371	*N/A*
ZBIST_1601	0.776065	9.214394	0.000653	0.004992	*SAM4*
ZBIST_2630	0.774108	9.216481	0.000672	0.005093	*SPF1*
ZBIST_1736	0.772012	6.769434	0.000702	0.005277	*GEA2*
ZBIST_4501	0.771717	5.992178	0.002663	0.014871	*CAR1*
ZBIST_1166	0.770366	5.284776	0.002739	0.015106	*ATG18*
ZBIST_0279	0.770056	8.13289	0.000809	0.0059	*RPT1*
ZBIST_3865	0.767266	5.755447	0.002163	0.012664	*N/A*
ZBIST_3360	0.767199	8.513698	0.000843	0.006081	*UGP1*
ZBIST_2495	0.766615	5.908236	0.002835	0.015514	*N/A*
ZBIST_4316	0.764306	5.067706	0.00689	0.029488	*LSB5*
ZBIST_4709	0.764205	8.761059	0.000844	0.006081	*YRB1*
ZBIST_3926	0.764164	9.329478	0.001004	0.006842	*SCL1*
ZBIST_3759	0.763699	8.616344	0.00173	0.010547	*RPN7*
ZBIST_3738	0.763384	6.600873	0.000807	0.0059	*MEC1*
ZBIST_3452	0.762236	8.553271	0.000902	0.006378	*XRN1*
ZBIST_0836	0.76191	6.903986	0.00313	0.016718	*N/A*
ZBIST_4254	0.761756	5.79802	0.000996	0.006801	*DSL1*
ZBIST_3361	0.761084	5.988886	0.000929	0.006496	*TUL1*
ZBIST_2484	0.756552	7.004102	0.001127	0.007467	*N/A*
ZBIST_0949	0.756462	5.279262	0.004033	0.020336	*RAD28*

ZBIST_0050	0.755377	6.766345	0.000904	0.006378	*GYP5*
ZBIST_3706	0.755152	7.895655	0.000988	0.006776	*RAD16*
ZBIST_4644	0.754796	5.067651	0.002642	0.014801	*RGD2*
ZBIST_3589	0.753934	5.819795	0.001124	0.007457	*CAP2*
ZBIST_3453	0.752265	5.626762	0.001322	0.008463	*NUP49*
ZBIST_4508	0.752169	6.228697	0.000964	0.006654	*MGM101*
ZBIST_3504	0.7518	6.934452	0.009147	0.037205	*N/A*
ZBIST_0549	0.751564	7.703317	0.001028	0.00698	*ARP3*
ZBIST_0188	0.749013	6.345396	0.002345	0.013525	*VRP1*
ZBIST_4424	0.748863	5.19382	0.002529	0.014341	*ECM27*
ZBIST_4744	0.748047	8.517951	0.007106	0.03017	*GPD1*
ZBIST_0107	0.746255	5.168826	0.00456	0.022047	*CEG1*
ZBIST_2364	0.744941	8.601185	0.001168	0.007653	*PHO13*
ZBIST_1652	0.744835	5.790543	0.004727	0.022696	*FOX2*
ZBIST_1239	0.744587	7.441349	0.001124	0.007457	*OXR1*
ZBIST_2063	0.74436	7.411714	0.001277	0.008227	*SUE1*
ZBIST_2839	0.743446	5.036107	0.002707	0.015016	*N/A*
ZBIST_1575	0.742138	9.850925	0.001988	0.011868	*PRE10*
ZBIST_2986	0.742023	6.309791	0.001122	0.007457	*N/A*
ZBIST_1645	0.741878	5.016872	0.004751	0.022745	*TFA1*
ZBIST_0905	0.740618	6.530661	0.001165	0.007648	*CTI6*
ZBIST_2460	0.739947	6.865075	0.001151	0.007576	*SEC6*
ZBIST_1923	0.739485	4.835081	0.004072	0.020445	*N/A*
ZBIST_2656	0.739316	5.883586	0.001346	0.00857	*UFD2*
ZBIST_4230	0.739294	5.742463	0.002932	0.015924	*PIN4*
ZBIST_1442	0.739218	5.544922	0.00162	0.00999	*UBP15*
ZBIST_4872	0.737607	6.716276	0.001876	0.011318	*ISN1*
ZBIST_1691	0.737518	4.404629	0.012662	0.047588	*N/A*
ZBIST_0160	0.736419	4.438778	0.00973	0.038982	*MSI1*
ZBIST_4162	0.735533	4.742081	0.005177	0.024198	*GTR1*
ZBIST_0696	0.733757	6.98321	0.001282	0.008247	*PRP8*
ZBIST_3226	0.732324	6.042317	0.002466	0.014075	*NIF3*
ZBIST_5067	0.732033	4.411874	0.011604	0.044603	*YJR096W*
ZBIST_2773	0.731828	4.633836	0.006878	0.029488	*RMD5*
ZBIST_2370	0.73092	5.867776	0.001516	0.009483	*N/A*
ZBIST_2162	0.729916	6.424116	0.001341	0.008549	*TAF12*
ZBIST_2908	0.729912	6.195332	0.002575	0.014526	*DMA2*
ZBIST_1114	0.729355	7.432058	0.00141	0.008899	*TCP1*
ZBIST_0774	0.725696	6.715542	0.001413	0.008908	*VBA1*
ZBIST_4133	0.724604	7.216461	0.00415	0.020692	*THI3*
ZBIST_3184	0.723492	9.143472	0.001487	0.009338	*PRE4*
ZBIST_4878	0.723266	7.30103	0.00154	0.009587	*RET1*
ZBIST_4792	0.721762	8.765891	0.001608	0.009949	*MNR2*
ZBIST_4675	0.721677	5.38203	0.004306	0.021169	*RIM15*
ZBIST_1777	0.721629	6.8371	0.001551	0.009623	*TIP41*
ZBIST_4668	0.721587	5.812607	0.012795	0.048026	*TUB2*
ZBIST_2442	0.718612	8.723203	0.001697	0.010393	*GET2*
ZBIST_4322	0.718333	5.747312	0.001997	0.011906	*GID7*
ZBIST_4208	0.717685	5.504243	0.002352	0.013546	*SLM1*
ZBIST_3650	0.715706	6.811814	0.001682	0.010328	*CCT2*

ZBIST_3198	0.715679	6.081282	0.001811	0.010969	*CSE1*
ZBIST_0865	0.715461	4.842193	0.005422	0.024849	*YKU80*
ZBIST_4827	0.715311	7.02794	0.001681	0.010328	*MDS3*
ZBIST_2599	0.715282	5.455131	0.004323	0.021169	*ULA1*
ZBIST_1068	0.714449	7.995978	0.001838	0.011102	*MAS1*
ZBIST_1701	0.71356	6.525686	0.003582	0.018533	*KNS1*
ZBIST_1947	0.71278	6.176926	0.003489	0.018166	*N/A*
ZBIST_0854	0.71211	5.803882	0.002971	0.016099	*N/A*
ZBIST_1083	0.712009	6.813734	0.001775	0.010779	*CDD1*
ZBIST_4546	0.711306	5.71359	0.00236	0.013575	*YPL088W*
ZBIST_2299	0.711162	5.870061	0.002049	0.012133	*LEU4*
ZBIST_1875	0.711048	7.739247	0.002451	0.014034	*FSH3*
ZBIST_2863	0.70935	6.110002	0.001902	0.01141	*RIB1*
ZBIST_0069	0.707479	6.837481	0.001887	0.01136	*N/A*
ZBIST_1489	0.70742	7.112764	0.001914	0.011463	*DOP1*
ZBIST_4054	0.705625	8.294554	0.002138	0.012548	*SSD1*
ZBIST_0691	0.705558	7.802737	0.002043	0.012119	*SOL3*
ZBIST_1799	0.705379	5.323531	0.003161	0.016811	*N/A*
ZBIST_4270	0.704894	5.304107	0.004515	0.02187	*CAN1*
ZBIST_1421	0.704638	4.502369	0.0115	0.044279	*N/A*
ZBIST_1294	0.704073	4.572458	0.010129	0.040256	*TFB1*
ZBIST_2254	0.703443	6.61656	0.002527	0.014341	*SCD6*
ZBIST_4726	0.702407	7.446779	0.002095	0.012341	*N/A*
ZBIST_3993	0.701259	7.559966	0.002135	0.012543	*NPC2*
ZBIST_3536	0.698437	5.567941	0.006432	0.028097	*N/A*
ZBIST_3437	0.698272	6.17827	0.005666	0.025565	*EDC3*
ZBIST_3402	0.69626	6.387736	0.002222	0.012947	*LST8*
ZBIST_2851	0.695356	5.780102	0.003438	0.017996	*SPT7*
ZBIST_2999	0.694655	6.533148	0.002236	0.013002	*WAR1*
ZBIST_2510	0.69447	6.233917	0.002281	0.01323	*MVP1*
ZBIST_3730	0.693616	4.754673	0.007983	0.033216	*OPY1*
ZBIST_0875	0.693455	5.957883	0.002531	0.014341	*MUB1*
ZBIST_0568	0.693241	6.788574	0.002296	0.013304	*SAC1*
ZBIST_3485	0.690721	4.924099	0.00669	0.028993	*MAG1*
ZBIST_2348	0.690686	6.354511	0.004416	0.021518	*YNL234W*
ZBIST_3821	0.689576	6.729063	0.00244	0.013989	*SWC3*
ZBIST_1373	0.689073	5.257529	0.006871	0.029488	*FRE8*
ZBIST_1962	0.689032	5.259356	0.004067	0.020441	*RPD3*
ZBIST_1781	0.687212	7.845547	0.005889	0.026307	*GRR1*
ZBIST_4561	0.686982	7.375249	0.002609	0.014653	*PBI2*
ZBIST_2055	0.68659	7.017709	0.00259	0.014591	*DER1*
ZBIST_2170	0.684551	7.294894	0.002688	0.014975	*CTH1*
ZBIST_1479	0.683472	5.529	0.00357	0.018518	*HOG1*
ZBIST_4189	0.682365	6.338922	0.002711	0.015018	*ARP5*
ZBIST_2304	0.682212	10.34849	0.002661	0.014871	*GPM1*
ZBIST_0105	0.681899	4.61598	0.012337	0.046592	*LEA1*
ZBIST_1253	0.680333	7.130498	0.00364	0.018735	*NPY1*
ZBIST_0686	0.67964	6.428152	0.002782	0.015292	*GLO4*
ZBIST_2966	0.678125	8.026774	0.003094	0.016619	*MLP1*
ZBIST_1982	0.677551	8.354512	0.003233	0.017161	*HRT1*

ZBIST_0092	0.677078	5.294988	0.004806	0.022875	*N/A*
ZBIST_4578	0.676662	7.651708	0.005108	0.023921	*N/A*
ZBIST_4194	0.675361	7.170608	0.00302	0.016312	*NUP159*
ZBIST_2982	0.675173	4.77257	0.009499	0.038399	*N/A*
ZBIST_4659	0.674736	6.308044	0.003014	0.016294	*BBC1*
ZBIST_1660	0.673654	5.623958	0.003861	0.01971	*PAP1*
ZBIST_1955	0.672535	5.576966	0.00413	0.020635	*DCP1*
ZBIST_2400	0.670526	5.203865	0.005511	0.025163	*N/A*
ZBIST_0140	0.66979	8.122942	0.003484	0.018166	*N/A*
ZBIST_3786	0.669	6.541769	0.003267	0.017283	*N/A*
ZBIST_1502	0.668609	4.745846	0.01316	0.049024	*SYN8*
ZBIST_0871	0.668502	5.529885	0.010838	0.042289	*N/A*
ZBIST_2032	0.66767	7.519072	0.003487	0.018166	*PET10*
ZBIST_3985	0.667094	6.1598	0.003446	0.018016	*PRP11*
ZBIST_0443	0.666883	6.262752	0.005419	0.024849	*SDS24*
ZBIST_1019	0.66557	5.779382	0.004374	0.021396	*MON2*
ZBIST_4376	0.665318	5.860146	0.005082	0.023844	*ATX2*
ZBIST_1946	0.664678	7.788761	0.00575	0.025824	*GET3*
ZBIST_3894	0.664464	5.89418	0.004951	0.02343	*PEX30*
ZBIST_4151	0.664377	5.823581	0.005616	0.025432	*RSC9*
ZBIST_2313	0.663425	7.414962	0.003638	0.018735	*SWD3*
ZBIST_3374	0.663179	6.037576	0.005548	0.025242	*SYM1*
ZBIST_2976	0.662796	5.602405	0.004581	0.022105	*DOA4*
ZBIST_4094	0.662705	5.939304	0.003903	0.019877	*N/A*
ZBIST_1256	0.66168	4.713803	0.012438	0.046901	*ALG2*
ZBIST_2256	0.66145	7.611522	0.004575	0.022098	*N/A*
ZBIST_3044	0.66067	7.295331	0.004258	0.02105	*MIA40*
ZBIST_2607	0.660077	6.999552	0.003711	0.019023	*NIC96*
ZBIST_2528	0.659289	7.922849	0.003968	0.020117	*N/A*
ZBIST_2318	0.659279	4.860568	0.009602	0.038603	*ATG18*
ZBIST_4474	0.657398	7.308269	0.003954	0.02008	*RPN5*
ZBIST_3862	0.657231	6.659462	0.007173	0.030417	*SCH9*
ZBIST_2159	0.656926	6.267097	0.011009	0.042848	*YPS1*
ZBIST_3086	0.656333	7.91113	0.004121	0.02063	*N/A*
ZBIST_3603	0.655823	7.688369	0.004118	0.02063	*RPB3*
ZBIST_2565	0.655617	5.76666	0.008815	0.036079	*GAT1*
ZBIST_2637	0.655479	7.584957	0.004147	0.020692	*RAD23*
ZBIST_0901	0.655445	7.130406	0.003989	0.020173	*APE1*
ZBIST_2186	0.654113	5.763983	0.00465	0.022368	*VHS3*
ZBIST_0709	0.654069	5.626529	0.005008	0.02363	*FMO1*
ZBIST_1134	0.651184	7.756049	0.0044	0.02148	*MSC3*
ZBIST_1012	0.64735	6.062836	0.005737	0.025815	*RFC3*
ZBIST_3676	0.647141	5.607524	0.005608	0.02542	*SEC8*
ZBIST_4012	0.647006	8.356882	0.004872	0.023125	*IRA2*
ZBIST_4653	0.645328	5.308431	0.010211	0.040515	*N/A*
ZBIST_2279	0.644512	5.722513	0.006974	0.029727	*N/A*
ZBIST_4656	0.644428	6.634785	0.004617	0.022234	*FET5*
ZBIST_2733	0.644262	5.79891	0.005216	0.024288	*N/A*
ZBIST_1054	0.642277	6.286109	0.004792	0.022851	*GAS4*
ZBIST_1993	0.642117	5.674255	0.011999	0.045659	*STE5*

ZBIST_3366	0.642112	7.172924	0.004771	0.022782	*N/A*
ZBIST_3994	0.641744	7.638732	0.004999	0.023613	*SIT4*
ZBIST_3795	0.641585	6.226292	0.004796	0.022851	*N/A*
ZBIST_2233	0.641451	10.19214	0.004706	0.022618	*SCS2*
ZBIST_3899	0.641368	6.559106	0.00474	0.022736	*BUD6*
ZBIST_4597	0.640801	5.803682	0.005636	0.025454	*N/A*
ZBIST_1147	0.636932	9.846269	0.004998	0.023613	*N/A*
ZBIST_3476	0.636795	6.604605	0.005051	0.023773	*GDI1*
ZBIST_1344	0.63676	5.185469	0.010208	0.040515	*DPB11*
ZBIST_2997	0.636353	5.800228	0.006808	0.029296	*ENT3*
ZBIST_0479	0.635401	6.352583	0.005137	0.024037	*BUD7*
ZBIST_1460	0.635202	7.272264	0.005352	0.02471	*N/A*
ZBIST_3618	0.635041	6.193425	0.005247	0.024361	*STS1*
ZBIST_2826	0.633374	7.401998	0.005532	0.025191	*CCT3*
ZBIST_3845	0.632648	5.077991	0.010086	0.040182	*ATG18*
ZBIST_0746	0.632378	7.233274	0.00545	0.024907	*PCP1*
ZBIST_3264	0.631133	5.246315	0.008593	0.035336	*MED4*
ZBIST_3364	0.629741	7.012057	0.00614	0.027134	*GSY1*
ZBIST_2561	0.629624	7.676374	0.005826	0.026072	*YBT1*
ZBIST_1062	0.629215	6.834986	0.005682	0.025614	*OXP1*
ZBIST_2075	0.629104	6.316779	0.005597	0.025415	*XKS1*
ZBIST_1675	0.628674	6.588656	0.005608	0.02542	*UFD4*
ZBIST_2873	0.628472	5.50101	0.008047	0.033433	*N/A*
ZBIST_3695	0.628252	7.493992	0.005976	0.026598	*ISA2*
ZBIST_0795	0.627876	7.818428	0.00608	0.026913	*YPT52*
ZBIST_4741	0.626955	4.962842	0.013209	0.049169	*RPN4*
ZBIST_5066	0.625996	8.314923	0.00643	0.028097	*JLP1*
ZBIST_1654	0.624608	5.85962	0.006683	0.028993	*RSC4*
ZBIST_2515	0.623729	7.635002	0.00635	0.027836	*HTZ1*
ZBIST_2626	0.62278	5.39475	0.008393	0.034637	*N/A*
ZBIST_3469	0.622114	7.840572	0.006551	0.028542	*PIM1*
ZBIST_0881	0.619538	6.20166	0.006507	0.028373	*RAD27*
ZBIST_0526	0.619184	7.941756	0.006801	0.029296	*MDG1*
ZBIST_1330	0.618578	6.086087	0.006646	0.028902	*VMA5*
ZBIST_1527	0.616351	7.321869	0.006773	0.029276	*MYO2*
ZBIST_3552	0.616309	5.385098	0.009384	0.03803	*FEN2*
ZBIST_3289	0.616057	11.19526	0.00669	0.028993	*TSA1*
ZBIST_3139	0.615803	6.169759	0.006723	0.02911	*ATG15*
ZBIST_1483	0.614892	5.345636	0.009523	0.038451	*HPR1*
ZBIST_1625	0.614642	6.494852	0.00676	0.029244	*YKL033W-A*
ZBIST_4203	0.614165	6.15976	0.006894	0.029488	*SEC24*
ZBIST_3739	0.612808	5.457888	0.009744	0.039006	*N/A*
ZBIST_4604	0.611848	7.299839	0.010075	0.040182	*TSL1*
ZBIST_1367	0.611313	10.70828	0.007067	0.030044	*TRX1*
ZBIST_0475	0.610465	6.463135	0.007187	0.030452	*N/A*
ZBIST_3417	0.609976	7.209706	0.007738	0.032368	*RVS161*
ZBIST_1375	0.609098	8.268806	0.007941	0.033076	*SEC26*
ZBIST_3617	0.608814	7.841592	0.007714	0.032295	*SQT1*
ZBIST_0029	0.608681	7.01037	0.007363	0.031118	*SGA1*
ZBIST_3053	0.607649	7.988401	0.009151	0.037205	*RPN13*

ZBIST_3840	0.607285	6.628612	0.007598	0.031916	*IFM1*
ZBIST_0216	0.607235	6.993722	0.008807	0.036075	*TFG1*
ZBIST_2553	0.604403	5.631108	0.01179	0.045041	*N/A*
ZBIST_3032	0.601546	6.589051	0.008076	0.033498	*CMP2*
ZBIST_2577	0.597757	5.98223	0.008991	0.036679	*IES1*
ZBIST_0603	0.597347	7.343005	0.008764	0.035961	*YPT6*
ZBIST_0605	0.597218	10.46074	0.008483	0.034924	*HSP60*
ZBIST_1837	0.594596	8.469368	0.009575	0.038581	*ERG20*
ZBIST_1912	0.593943	7.694545	0.00929	0.037681	*MET2*
ZBIST_3496	0.593136	7.231757	0.009251	0.03755	*TPS2*
ZBIST_1454	0.591495	5.740214	0.010557	0.041554	*IOC2*
ZBIST_4912	0.589686	6.927007	0.009604	0.038603	*ARC19*
ZBIST_1700	0.588833	6.689903	0.009583	0.038582	*N/A*
ZBIST_3216	0.588378	5.890976	0.010518	0.041467	*MXR1*
ZBIST_3741	0.585504	5.78562	0.011133	0.043235	*TWF1*
ZBIST_4574	0.585321	7.849361	0.010644	0.041766	*AVT7*

(1) logFC - logarithm base 2 of fold change.
(2) logCPM - logarithm base 2 of counts per million.
(3) FDR - false discovery rate

Supplementary Table S7. Genes found to have decreased mRNA levels (downregulated) in the *Z. bailii* IST302 *Zbhaa1*Δ deletion mutant strain upon sudden exposure to copper stress compared with unstressed cells.

Z. bailii ORF	logFC (1)	logCPM (2)	PValue	FDR (3)	*S. cerevisiae* Homologue
ZBIST_3609	-5.968404	1.255083	0.00062	0.004764	*N/A*
ZBIST_4639	-5.155931	9.848605	2.03E-13	1E-09	*ZRT1*
ZBIST_5053	-4.286246	2.59097	1.53E-05	0.000313	*HNM1*
ZBIST_5080	-4.264739	1.469136	0.000665	0.00506	*N/A*
ZBIST_5094	-4.079751	2.356976	0.000172	0.001737	*DAL5*
ZBIST_4991	-3.831905	5.791035	6.96E-11	5.72E-08	*TPO3*
ZBIST_1605	-3.567907	3.089209	5.74E-06	0.000166	*PHO3*
ZBIST_5037	-3.28454	3.919125	3.93E-07	3.08E-05	*N/A*
ZBIST_5025	-3.243054	1.352671	0.003399	0.017827	*N/A*
ZBIST_1696	-3.088389	7.258282	5.59E-10	2.51E-07	*N/A*
ZBIST_1597	-2.55411	2.291459	0.000995	0.006801	*N/A*
ZBIST_3512	-2.501576	2.779243	0.000102	0.001149	*AGP3*
ZBIST_5089	-2.491189	6.866842	1.72E-09	6.53E-07	*YJL218W*
ZBIST_5097	-2.476522	5.901158	1.7E-07	1.87E-05	*FIT2*
ZBIST_0771	-2.386944	1.874026	0.002175	0.012704	*N/A*
ZBIST_2523	-2.378824	2.551845	0.000457	0.00373	*N/A*
ZBIST_3714	-2.361202	3.482884	6.82E-06	0.000179	*N/A*
ZBIST_1388	-2.360289	5.849915	1.46E-08	3.27E-06	*ADR1*
ZBIST_1004	-2.312798	2.288125	0.002517	0.014316	*POP3*
ZBIST_3033	-2.288682	9.334614	1.06E-08	2.5E-06	*IMD4*
ZBIST_0055	-2.263473	5.997096	1.74E-08	3.46E-06	*GCV2*
ZBIST_3145	-2.24173	5.585243	0.002069	0.012217	*N/A*
ZBIST_4769	-2.186686	1.941199	0.012245	0.046387	*N/A*
ZBIST_5078	-2.182711	1.713214	0.007832	0.032679	*INP1*
ZBIST_4938	-2.143377	6.215739	6.51E-09	1.78E-06	*MAK11*
ZBIST_1917	-2.118477	3.761122	1.14E-05	0.000251	*RPF1*
ZBIST_3511	-2.105706	4.145055	4.7E-06	0.000144	*VBA2*
ZBIST_4391	-2.099153	5.803632	6.29E-08	9.7E-06	*RPF2*
ZBIST_2858	-2.079871	5.426998	8.84E-08	1.25E-05	*N/A*
ZBIST_0278	-2.077429	5.111823	3.73E-06	0.000128	*RPC25*
ZBIST_1522	-2.052934	5.988276	4.47E-08	7.15E-06	*MAK16*
ZBIST_0325	-1.985153	6.851299	1.8E-08	3.46E-06	*RRB1*
ZBIST_1595	-1.945115	8.021645	2.48E-05	0.000413	*N/A*
ZBIST_3445	-1.917243	4.768839	0.001013	0.006895	*FDH1*
ZBIST_0156	-1.907609	5.781353	4.08E-07	3.15E-05	*RIM2*
ZBIST_2530	-1.886099	5.665785	2.73E-07	2.42E-05	*ENP2*
ZBIST_3959	-1.881112	11.37145	3.65E-06	0.000128	*RPL27a*
ZBIST_1746	-1.865729	6.19605	3.01E-06	0.000116	*N/A*
ZBIST_4679	-1.864357	9.024959	6.5E-08	9.72E-06	*SHM2*
ZBIST_4651	-1.839281	6.364567	4.92E-07	3.57E-05	*BUD20*
ZBIST_1234	-1.834462	2.181667	0.005806	0.026006	*RSA1*
ZBIST_3342	-1.825381	10.6322	1.08E-06	5.81E-05	*RPL12a*
ZBIST_1592	-1.820466	5.04763	3.91E-06	0.000131	*ECM1*
ZBIST_1295	-1.816135	4.475807	3.36E-06	0.000125	*RRP3*
ZBIST_2897	-1.816	5.620645	2.3E-07	2.19E-05	*NOP15*
ZBIST_3356	-1.813219	11.2584	5.28E-05	0.000699	*RPS20*
ZBIST_4454	-1.796819	5.206819	6.47E-06	0.000174	*N/A*

ZBIST_0523	-1.790099	2.823383	0.002219	0.012947	*DCD1*
ZBIST_1205	-1.788843	5.388547	6.09E-07	4.01E-05	*N/A*
ZBIST_5119	-1.770949	8.092633	2.03E-07	2.1E-05	*N/A*
ZBIST_0258	-1.754373	5.886622	8.01E-08	1.16E-05	*RLP24*
ZBIST_0268	-1.733392	3.138749	0.00041	0.003406	*PML1*
ZBIST_3876	-1.730685	1.991053	0.008785	0.036016	*SIT1*
ZBIST_4768	-1.72697	2.836874	0.004414	0.021518	*DIM1*
ZBIST_2120	-1.718613	5.240483	6.99E-07	4.43E-05	*YTM1*
ZBIST_2521	-1.709335	12.10174	2E-06	8.49E-05	*RPL30*
ZBIST_4179	-1.70446	6.879323	5.47E-07	3.75E-05	*RPL9b*
ZBIST_4187	-1.704333	4.196447	5.19E-05	0.00069	*GCD10*
ZBIST_4893	-1.699759	6.375929	7.35E-07	4.49E-05	*PNO1*
ZBIST_0921	-1.693026	10.68519	1.95E-07	2.09E-05	*RPL4a*
ZBIST_4021	-1.691382	5.532754	0.000617	0.00476	*CIC1*
ZBIST_1854	-1.686097	7.34358	3.44E-07	2.88E-05	*NSA2*
ZBIST_1039	-1.678883	5.85227	5.39E-07	3.75E-05	*TRM11*
ZBIST_1359	-1.675254	3.945263	0.000307	0.002745	*DBP7*
ZBIST_5079	-1.667946	5.756356	3.76E-07	3.01E-05	*GCN4*
ZBIST_2196	-1.663718	9.713364	5.25E-07	3.75E-05	*RPL3*
ZBIST_4705	-1.662308	7.755174	3.15E-05	0.000488	*HTA2*
ZBIST_1194	-1.660715	8.022173	8.2E-07	4.82E-05	*ADE13*
ZBIST_2548	-1.658754	8.742229	1.2E-06	6.24E-05	*NSR1*
ZBIST_3158	-1.653551	4.640979	5.31E-06	0.000159	*BUD23*
ZBIST_3523	-1.65141	6.187538	4.34E-07	3.21E-05	*NOG2*
ZBIST_4392	-1.644116	5.644442	1.89E-06	8.46E-05	*MTD1*
ZBIST_2901	-1.633783	3.053566	0.002014	0.011994	*DBP2*
ZBIST_2426	-1.633752	11.71964	1.28E-06	6.33E-05	*RPP1b*
ZBIST_4067	-1.627408	6.83847	5.96E-07	3.97E-05	*UTP10*
ZBIST_2900	-1.613663	9.029705	3.96E-06	0.000132	*DBP2*
ZBIST_2236	-1.612301	6.411381	2.29E-07	2.19E-05	*NOC4*
ZBIST_2456	-1.600315	11.12632	1.68E-05	0.000325	*RPS24b*
ZBIST_1909	-1.598844	6.651093	2.22E-07	2.19E-05	*N/A*
ZBIST_0309	-1.598641	10.18708	2.03E-06	8.49E-05	*RPL15b*
ZBIST_1907	-1.587593	9.121278	0.000862	0.006187	*GAL2*
ZBIST_3165	-1.579964	4.729469	1.6E-05	0.000319	*SMM1*
ZBIST_2029	-1.574848	7.461109	1.19E-07	1.51E-05	*N/A*
ZBIST_1035	-1.572694	11.33487	6.88E-06	0.000179	*RPL18a*
ZBIST_4706	-1.568751	8.784843	9.67E-07	5.42E-05	*HTB1*
ZBIST_2677	-1.565728	5.531826	3.11E-05	0.000484	*NAN1*
ZBIST_0936	-1.564265	6.023834	4.25E-07	3.21E-05	*GCV1*
ZBIST_1382	-1.560129	4.573065	8.37E-06	0.000204	*MNN10*
ZBIST_2690	-1.55224	11.00152	3.78E-06	0.000128	*RPL5*
ZBIST_0674	-1.545744	3.906579	0.00012	0.001301	*EXO1*
ZBIST_0903	-1.545261	8.484604	1.25E-07	1.51E-05	*ADH3*
ZBIST_3893	-1.543672	9.655463	0.004005	0.020211	*RPL38*
ZBIST_4447	-1.53879	7.081901	1.35E-07	1.55E-05	*N/A*
ZBIST_0589	-1.531256	7.424939	5.44E-07	3.75E-05	*RRP5*
ZBIST_0067	-1.529986	4.60232	3.02E-05	0.000478	*YAR1*
ZBIST_3428	-1.528896	5.573452	3.55E-06	0.000128	*SNF3*
ZBIST_2866	-1.526901	4.127488	0.0001	0.001139	*POL12*

ZBIST_1164	-1.522381	7.578245	2.6E-07	2.38E-05	*ILV3*
ZBIST_0098	-1.519165	12.01464	1.83E-06	8.35E-05	*RPL1a*
ZBIST_2758	-1.515111	10.17776	4.2E-06	0.000136	*RPL9b*
ZBIST_0688	-1.514316	5.491289	5.76E-06	0.000166	*SDA1*
ZBIST_2251	-1.513862	12.8198	0.000117	0.001279	*RPS23b*
ZBIST_0748	-1.507934	6.912631	2.45E-07	2.28E-05	*NOP7*
ZBIST_1042	-1.504315	7.405394	3.48E-07	2.88E-05	*MDH2*
ZBIST_4312	-1.496472	4.686918	3.29E-05	0.0005	*RRP7*
ZBIST_0772	-1.487063	5.437837	7.38E-06	0.000189	*ENP1*
ZBIST_0890	-1.486414	2.533458	0.006001	0.026637	*CTF13*
ZBIST_1667	-1.468185	11.32922	3.67E-05	0.000545	*RPL14a*
ZBIST_3258	-1.467445	6.092381	8.55E-06	0.000206	*DBP9*
ZBIST_4293	-1.462214	11.33023	9.86E-06	0.000227	*RPL37a*
ZBIST_0512	-1.459487	12.33384	1.66E-05	0.000325	*RPL42a*
ZBIST_4780	-1.45769	6.239385	1.53E-06	7.27E-05	*ERB1*
ZBIST_2326	-1.455413	8.266002	2.22E-07	2.19E-05	*NOG1*
ZBIST_2929	-1.451572	6.249569	7.12E-06	0.000183	*KRE33*
ZBIST_3105	-1.441224	6.020852	1.27E-06	6.33E-05	*TSR1*
ZBIST_4088	-1.43857	5.235575	5.67E-05	0.000733	*N/A*
ZBIST_3610	-1.436343	11.93151	1.64E-05	0.000325	*RPL2b*
ZBIST_4915	-1.433321	4.931594	2.35E-05	0.000407	*URB1*
ZBIST_3295	-1.431308	4.758957	7.89E-05	0.000931	*UTP6*
ZBIST_2511	-1.431221	5.647857	0.004435	0.02157	*RCL1*
ZBIST_1991	-1.43041	6.114801	6.5E-06	0.000174	*ARX1*
ZBIST_3976	-1.429591	6.096314	6.03E-06	0.00017	*DBP10*
ZBIST_4805	-1.428367	3.248036	0.002309	0.013361	*ASK1*
ZBIST_2892	-1.427373	8.859872	1.67E-06	7.76E-05	*LEU4*
ZBIST_2809	-1.42585	5.607261	6.87E-06	0.000179	*NOP16*
ZBIST_1172	-1.425778	4.446297	0.004127	0.020635	*N/A*
ZBIST_1296	-1.425728	5.822556	1.31E-05	0.000282	*SSF1*
ZBIST_2913	-1.425146	3.739589	0.001386	0.008785	*COX23*
ZBIST_1395	-1.415786	8.447677	8.03E-07	4.78E-05	*N/A*
ZBIST_0464	-1.412995	10.33987	1.4E-05	0.000293	*RPL20b*
ZBIST_5142	-1.411891	9.975031	3.37E-06	0.000125	*N/A*
ZBIST_0945	-1.408046	10.45415	1.97E-06	8.49E-05	*RPS11a*
ZBIST_1036	-1.407529	11.7841	4.66E-06	0.000144	*RPS19a*
ZBIST_1242	-1.406541	12.04848	3.36E-06	0.000125	*RPL7a*
ZBIST_3202	-1.405397	8.683264	1.01E-06	5.56E-05	*ADE5,7*
ZBIST_4983	-1.402535	4.394753	6.26E-05	0.000792	*PRI2*
ZBIST_2119	-1.402312	7.443211	1.67E-06	7.76E-05	*FSF1*
ZBIST_1584	-1.399687	3.41884	0.003973	0.020117	*RAD17*
ZBIST_2747	-1.39271	3.712382	0.001044	0.007049	*N/A*
ZBIST_3345	-1.392655	5.529167	6.66E-06	0.000176	*BUD22*
ZBIST_0179	-1.392638	11.1722	3.45E-05	0.00052	*RPL26a*
ZBIST_0269	-1.392153	5.350131	9.88E-06	0.000227	*HCA4*
ZBIST_2876	-1.391211	9.607114	6.39E-07	4.15E-05	*RPS7a*
ZBIST_0852	-1.386405	11.50521	3.46E-05	0.00052	*ASC1*
ZBIST_0132	-1.38323	3.096024	0.003063	0.016508	*N/A*
ZBIST_0340	-1.383091	12.99762	0.000546	0.004301	*RPP1a*
ZBIST_0979	-1.380928	4.810457	3.28E-05	0.0005	*TRM10*

ZBIST_1585	-1.379499	12.70844	0.000582	0.004543	*RPS12*
ZBIST_0110	-1.371997	5.541152	3.79E-06	0.000128	*NIP7*
ZBIST_3454	-1.371895	4.759599	3.3E-05	0.000502	*ROK1*
ZBIST_3024	-1.3634	4.479706	0.000135	0.00143	*OGG1*
ZBIST_2711	-1.361146	5.268363	1.43E-05	0.000295	*NOP53*
ZBIST_1561	-1.360817	3.973813	0.000905	0.006378	*GDS1*
ZBIST_1187	-1.360648	10.61497	2.94E-06	0.000114	*ILV5*
ZBIST_2835	-1.359713	5.731994	2.72E-06	0.000108	*N/A*
ZBIST_3001	-1.359369	8.546734	5.94E-07	3.97E-05	*FPR3*
ZBIST_3286	-1.351716	5.061164	6.56E-06	0.000175	*GUK1*
ZBIST_0651	-1.350432	4.743023	4.07E-05	0.000582	*LRP1*
ZBIST_0583	-1.346869	5.495731	5.84E-06	0.000168	*RRS1*
ZBIST_3209	-1.345245	3.54258	0.001324	0.008463	*N/A*
ZBIST_2591	-1.343746	6.47845	2.21E-05	0.000389	*RRP12*
ZBIST_1074	-1.338694	4.008405	0.002692	0.01498	*SAS4*
ZBIST_0961	-1.33709	7.444455	0.000566	0.004458	*SUP45*
ZBIST_4807	-1.336812	7.239094	3.56E-06	0.000128	*LCP5*
ZBIST_4188	-1.33625	6.862317	7E-07	4.43E-05	*NOP2*
ZBIST_5112	-1.334115	4.862818	0.000341	0.002993	*ARR3*
ZBIST_2178	-1.332964	3.386686	0.008598	0.035336	*RSB1*
ZBIST_1980	-1.33294	6.949253	6.19E-06	0.000171	*PFK27*
ZBIST_2998	-1.332358	3.397497	0.002897	0.015759	*BET5*
ZBIST_0957	-1.326603	3.384388	0.005241	0.024357	*GAL7*
ZBIST_4280	-1.325894	10.51684	1.94E-06	8.49E-05	*GOR1*
ZBIST_4672	-1.322265	11.36979	7.92E-05	0.000931	*RPL22a*
ZBIST_1240	-1.320661	5.268762	8.29E-06	0.000202	*DBP3*
ZBIST_2276	-1.318161	9.322356	2.03E-06	8.49E-05	*RPL36b*
ZBIST_1589	-1.318039	5.407174	1.55E-05	0.000315	*NUD1*
ZBIST_3297	-1.317583	11.28381	8.25E-06	0.000202	*RPS17a*
ZBIST_1439	-1.313823	6.631536	2.38E-05	0.000409	*ADE4*
ZBIST_1787	-1.312662	11.97008	2.26E-05	0.000395	*RPL43a*
ZBIST_1300	-1.304191	4.175278	0.000774	0.005694	*IPK1*
ZBIST_1717	-1.303418	6.364077	7.75E-05	0.000917	*RPA135*
ZBIST_2227	-1.300699	13.0264	1.23E-05	0.000268	*RPL23b*
ZBIST_2944	-1.299686	4.709142	0.000192	0.001873	*RPC31*
ZBIST_0466	-1.293514	8.399602	4.49E-06	0.000143	*NOP58*
ZBIST_2592	-1.292118	5.916886	2.73E-05	0.000442	*MRPS16*
ZBIST_2813	-1.287402	7.991422	2.23E-06	9.12E-05	*NUG1*
ZBIST_4061	-1.284048	5.970091	4.99E-06	0.000151	*SUR2*
ZBIST_2604	-1.283244	4.793992	0.000253	0.002349	*LOC1*
ZBIST_0476	-1.279376	8.052549	1.9E-06	8.46E-05	*CPA1*
ZBIST_4735	-1.278814	6.963943	8.63E-06	0.000207	*ADE1*
ZBIST_0719	-1.277257	11.44466	9.76E-06	0.000226	*RPS22a*
ZBIST_2861	-1.268307	12.17896	5.52E-05	0.000719	*PET9*
ZBIST_2218	-1.264369	8.585459	2.06E-05	0.000375	*KAP123*
ZBIST_3753	-1.260261	10.94164	6.3E-06	0.000171	*RPL11a*
ZBIST_1501	-1.254538	5.749835	7.69E-05	0.000915	*NOP12*
ZBIST_1128	-1.252464	5.715188	1.21E-05	0.000264	*IFH1*
ZBIST_4240	-1.252256	5.974065	8.01E-06	0.0002	*JJJ3*
ZBIST_1349	-1.252021	5.325055	2.4E-05	0.000409	*MRPL49*

ZBIST_0586	-1.24693	10.47205	2.58E-05	0.000422	*RPS10a*
ZBIST_0720	-1.246478	12.52441	0.000304	0.002724	*RPS14b*
ZBIST_1078	-1.246013	12.93967	0.001394	0.008822	*RPS31*
ZBIST_3766	-1.245919	5.973731	4.16E-06	0.000136	*ACO1*
ZBIST_0727	-1.245163	5.074785	0.000197	0.001922	*UTP22*
ZBIST_4621	-1.243461	3.309903	0.005427	0.024849	*N/A*
ZBIST_0892	-1.241516	5.506628	0.000187	0.001841	*UTP15*
ZBIST_0547	-1.241261	5.893989	9.56E-06	0.000226	*RPA12*
ZBIST_0954	-1.239584	6.990885	2.57E-05	0.000422	*N/A*
ZBIST_0953	-1.237838	8.132543	2.75E-05	0.000444	*YRO2*
ZBIST_0824	-1.233189	5.9427	2.02E-05	0.000371	*REI1*
ZBIST_1420	-1.233151	5.865285	1.75E-05	0.000332	*HAS1*
ZBIST_0695	-1.230153	3.156146	0.005362	0.02471	*N/A*
ZBIST_2706	-1.229705	10.84279	2.42E-05	0.000411	*RPL33a*
ZBIST_4164	-1.228997	7.589226	4.67E-06	0.000144	*MSK1*
ZBIST_0575	-1.228505	3.054927	0.005213	0.024288	*N/A*
ZBIST_2857	-1.223358	11.18937	1.46E-05	0.000301	*RPL19a*
ZBIST_1358	-1.222697	6.615024	3.73E-06	0.000128	*RPC37*
ZBIST_0185	-1.221691	12.44592	0.000108	0.001211	*RPP0*
ZBIST_3713	-1.220716	14.24842	0.000696	0.005244	*N/A*
ZBIST_4987	-1.220443	5.521761	9.5E-05	0.001088	*YCT1*
ZBIST_0342	-1.220013	11.34746	4.12E-05	0.000586	*RPS16a*
ZBIST_1720	-1.215828	3.16573	0.00841	0.03465	*N/A*
ZBIST_3089	-1.209617	3.951058	0.000928	0.006496	*N/A*
ZBIST_1332	-1.207287	6.00875	2.77E-05	0.000445	*RRP14*
ZBIST_0296	-1.199472	7.506091	6.92E-06	0.000179	*SHM1*
ZBIST_2330	-1.198141	10.81504	8.19E-06	0.000202	*RPS6a*
ZBIST_3977	-1.196943	3.960072	0.002078	0.012255	*SLM3*
ZBIST_4100	-1.196781	4.332927	0.00061	0.004712	*TRM82*
ZBIST_0812	-1.195434	6.265188	3.28E-05	0.0005	*UTP18*
ZBIST_4172	-1.189732	10.0445	5.51E-06	0.000162	*RPL16a*
ZBIST_4525	-1.189112	4.919369	4.44E-05	0.000621	*UTP9*
ZBIST_2010	-1.187531	10.67019	5.13E-05	0.000685	*RPS21b*
ZBIST_1350	-1.186196	5.564187	4.93E-05	0.000665	*N/A*
ZBIST_1204	-1.185663	8.882274	1.4E-05	0.000293	*ADE6*
ZBIST_0922	-1.183755	6.654878	9.7E-06	0.000226	*HMT1*
ZBIST_1544	-1.18221	7.370466	3.67E-06	0.000128	*RPA190*
ZBIST_3860	-1.180188	12.36715	5.88E-05	0.000757	*RPS4a*
ZBIST_0155	-1.180112	10.29565	9.11E-06	0.000217	*RPL21a*
ZBIST_0872	-1.179684	7.329236	1.76E-05	0.000333	*OAC1*
ZBIST_1241	-1.179358	6.231536	2.23E-05	0.000392	*HNM1*
ZBIST_3740	-1.177299	6.780079	5.95E-06	0.000169	*N/A*
ZBIST_0572	-1.176732	3.724132	0.004177	0.020763	*YMC1*
ZBIST_1044	-1.174352	11.06053	0.000167	0.001699	*RPL25*
ZBIST_2563	-1.172009	10.99638	0.000106	0.001192	*RPL8b*
ZBIST_1498	-1.171684	11.84621	0.000116	0.001271	*RPP2a*
ZBIST_0783	-1.171281	14.16122	0.000445	0.003644	*RPL28*
ZBIST_2234	-1.170852	10.822	1.56E-05	0.000316	*RPL32*
ZBIST_3002	-1.170059	10.96446	1.69E-05	0.000325	*RPL6b*
ZBIST_3608	-1.169443	9.967482	1.03E-05	0.000235	*N/A*

ZBIST_0193	-1.166805	12.30759	4.79E-05	0.000652	*RPS25a*
ZBIST_1914	-1.165924	10.0683	4.25E-05	0.0006	*GAR1*
ZBIST_3412	-1.164847	5.112156	6.71E-05	0.000832	*MRPL32*
ZBIST_2481	-1.160228	11.50675	3.62E-05	0.00054	*RPL17b*
ZBIST_3092	-1.157903	9.106505	0.000255	0.002355	*COX9*
ZBIST_3527	-1.155744	9.656202	1.1E-05	0.000246	*LYS9*
ZBIST_3055	-1.154453	4.682662	0.000465	0.003785	*DPH2*
ZBIST_1274	-1.154252	5.312598	4.17E-05	0.000591	*BFR2*
ZBIST_0473	-1.153925	4.911036	0.010718	0.04192	*RNT1*
ZBIST_4483	-1.153652	4.268618	0.001148	0.007565	*N/A*
ZBIST_1586	-1.150259	5.757379	3.04E-05	0.000478	*MRS6*
ZBIST_3079	-1.150126	10.12545	2.15E-05	0.000381	*RPL31a*
ZBIST_1214	-1.148972	4.839286	0.000102	0.001149	*RIX7*
ZBIST_0420	-1.146441	11.88053	7.45E-05	0.00089	*RPS2*
ZBIST_4432	-1.143716	6.260164	3.98E-05	0.000576	*RPA34*
ZBIST_1301	-1.141416	7.468491	1.37E-05	0.00029	*DYS1*
ZBIST_3539	-1.138393	4.920862	0.000392	0.003308	*N/A*
ZBIST_0960	-1.135814	5.971235	9.6E-06	0.000226	*MAK5*
ZBIST_3427	-1.134251	4.632781	0.001121	0.007457	*N/A*
ZBIST_2518	-1.134131	5.333895	2.45E-05	0.000413	*CGR1*
ZBIST_1674	-1.133454	7.148927	1.24E-05	0.000268	*MRT4*
ZBIST_4818	-1.130225	10.26061	2.13E-05	0.000379	*RPS26a*
ZBIST_1620	-1.129104	13.85677	0.002756	0.015184	*RPP2b*
ZBIST_0998	-1.125788	6.043272	1.06E-05	0.000238	*PUF6*
ZBIST_4343	-1.125122	6.384081	8.88E-05	0.001029	*SPB1*
ZBIST_0257	-1.124365	7.037206	1.05E-05	0.000238	*PAM18*
ZBIST_1133	-1.121467	8.397884	8.29E-06	0.000202	*HTA2*
ZBIST_0959	-1.118452	3.603209	0.011207	0.043352	*N/A*
ZBIST_3104	-1.116083	11.45812	9.31E-05	0.001071	*RPS29b*
ZBIST_1058	-1.115536	3.268047	0.00777	0.032448	*N/A*
ZBIST_4291	-1.114664	6.936891	7.5E-06	0.000191	*EMG1*
ZBIST_4320	-1.111299	5.522966	2.46E-05	0.000413	*SRO9*
ZBIST_4387	-1.109884	3.882618	0.007259	0.030731	*CDC21*
ZBIST_4349	-1.109439	3.509811	0.006833	0.029379	*N/A*
ZBIST_1144	-1.10718	5.129626	0.000135	0.001429	*COX20*
ZBIST_1985	-1.106478	3.967497	0.008728	0.03584	*TMT1*
ZBIST_2268	-1.101127	5.434209	5.51E-05	0.000719	*CLB5*
ZBIST_0341	-1.10057	10.85567	2.4E-05	0.000409	*RPL13a*
ZBIST_1543	-1.100139	5.74052	4.46E-05	0.000622	*RPA43*
ZBIST_3416	-1.100138	5.413392	0.000367	0.003132	*N/A*
ZBIST_4671	-1.099894	5.761382	2.05E-05	0.000375	*N/A*
ZBIST_2853	-1.096365	5.392035	3.95E-05	0.000574	*MAK21*
ZBIST_4294	-1.093666	4.978892	0.00038	0.003231	*PLM2*
ZBIST_2005	-1.089004	3.14572	0.009953	0.039746	*RPB4*
ZBIST_1249	-1.085988	7.417928	2.12E-05	0.000379	*MNP1*
ZBIST_1838	-1.085523	6.796247	1.72E-05	0.000329	*EBP2*
ZBIST_3358	-1.081411	5.226783	0.000107	0.001199	*N/A*
ZBIST_3020	-1.079494	12.62657	0.001524	0.009496	*RPS1a*
ZBIST_3404	-1.077426	8.144826	1.7E-05	0.000326	*CBF5*
ZBIST_0801	-1.077215	6.172779	1.66E-05	0.000325	*SMC3*

ZBIST_4239	-1.07468	4.691351	0.000259	0.002385	*N/A*
ZBIST_4471	-1.074002	4.45593	0.002485	0.014165	*RPC53*
ZBIST_0299	-1.073803	3.80689	0.011207	0.043352	*CHK1*
ZBIST_2965	-1.07351	10.33923	7.52E-05	0.000897	*RPL40b*
ZBIST_2380	-1.067532	11.42917	4.27E-05	0.000601	*SSB2*
ZBIST_4298	-1.06069	5.209964	0.000125	0.001356	*LPP1*
ZBIST_4297	-1.059971	8.782969	6.79E-05	0.00084	*SAM2*
ZBIST_3149	-1.057717	5.374762	0.00021	0.00203	*PWP2*
ZBIST_1094	-1.057357	6.018555	0.000393	0.003308	*MSS116*
ZBIST_1422	-1.053132	5.368619	0.000277	0.00251	*TDA1*
ZBIST_2146	-1.053094	3.413626	0.006075	0.026913	*RIO1*
ZBIST_1626	-1.050329	6.225411	1.68E-05	0.000325	*PRS3*
ZBIST_1070	-1.048991	4.365547	0.002552	0.014431	*CSN9*
ZBIST_0378	-1.048674	6.6197	0.003684	0.018942	*RLI1*
ZBIST_3479	-1.0466	6.053104	0.000143	0.001485	*N/A*
ZBIST_5090	-1.045934	5.612866	6.93E-05	0.000844	*DAL3*
ZBIST_2480	-1.039921	9.066376	0.000144	0.001497	*MMF1*
ZBIST_0527	-1.03881	5.411036	0.000291	0.002612	*NOP13*
ZBIST_0729	-1.038673	5.436419	6.95E-05	0.000844	*RPC40*
ZBIST_1883	-1.035102	9.844115	0.000111	0.001234	*GUA1*
ZBIST_2517	-1.034461	5.406024	0.000185	0.001833	*RRN11*
ZBIST_1562	-1.031214	6.482932	7.36E-05	0.000881	*CIR2*
ZBIST_0154	-1.031188	11.52095	0.000363	0.003115	*RPS9a*
ZBIST_4869	-1.024847	4.862114	0.00019	0.00187	*SME1*
ZBIST_3522	-1.024781	5.298802	7.17E-05	0.000863	*ESF2*
ZBIST_3761	-1.023037	6.376911	0.001746	0.010624	*ELO2*
ZBIST_2697	-1.022092	6.830435	1.6E-05	0.000319	*RPB8*
ZBIST_4449	-1.021672	6.596544	5.94E-05	0.000763	*NOP56*
ZBIST_2981	-1.020649	5.752269	0.000888	0.006303	*POT1*
ZBIST_4677	-1.020277	8.61456	7.72E-05	0.000915	*HAC1*
ZBIST_3101	-1.019398	5.804806	7.98E-05	0.000936	*N/A*
ZBIST_0397	-1.018819	6.113679	0.003151	0.016777	*N/A*
ZBIST_0599	-1.018607	9.657122	0.000102	0.001149	*RPS28b*
ZBIST_2455	-1.016347	7.902548	1.66E-05	0.000325	*YOS1*
ZBIST_3031	-1.013636	5.509596	0.000312	0.002788	*TSR2*
ZBIST_4470	-1.007733	6.484449	4.77E-05	0.00065	*SAS10*
ZBIST_4518	-1.007673	9.179754	0.000363	0.003113	*MET5*
ZBIST_0445	-1.007392	8.484338	5.33E-05	0.000704	*OLE1*
ZBIST_3293	-1.005413	10.51751	4.06E-05	0.000582	*RPS18b*
ZBIST_2635	-1.005152	12.61	0.000741	0.005517	*HYP2*
ZBIST_4473	-1.002201	6.274558	5.57E-05	0.000721	*NOP14*
ZBIST_2961	-1.000789	3.160172	0.012065	0.04581	*PAN6*
ZBIST_0861	-0.999201	7.879665	6.98E-05	0.000845	*ILV2*
ZBIST_2902	-0.997982	6.161887	3.03E-05	0.000478	*RPC19*
ZBIST_3455	-0.996431	7.210349	0.000857	0.006164	*ATP20*
ZBIST_0602	-0.996409	6.547054	0.00018	0.001804	*N/A*
ZBIST_3275	-0.99539	4.211425	0.003298	0.017412	*MRPL28*
ZBIST_2418	-0.994772	5.132222	0.001194	0.007776	*N/A*
ZBIST_3480	-0.994588	10.54588	0.000236	0.002218	*RPS13*
ZBIST_0224	-0.992815	3.673276	0.008139	0.033732	*YEH2*

ZBIST_006	-0.988021	11.72999	0.000132	0.00141	*N/A*
ZBIST_2205	-0.98652	11.96823	0.00028	0.002532	*RPS8b*
ZBIST_3559	-0.984439	11.00618	0.000115	0.001269	*RPL34b*
ZBIST_2898	-0.983998	9.067738	6.05E-05	0.00077	*CYB5*
ZBIST_0763	-0.983312	4.808023	0.000872	0.006239	*UTP11*
ZBIST_1697	-0.98166	7.137886	2.06E-05	0.000375	*SPA2*
ZBIST_2675	-0.981606	3.990552	0.01251	0.047064	*KAP120*
ZBIST_4371	-0.972015	8.530321	6.51E-05	0.000815	*TIF4631*
ZBIST_4227	-0.971791	5.034532	0.000807	0.0059	*N/A*
ZBIST_0701	-0.971517	4.893311	0.000349	0.003052	*NOP19*
ZBIST_2487	-0.969658	5.357333	0.000405	0.003385	*UTP23*
ZBIST_1430	-0.969315	4.65155	0.000778	0.005714	*YGR272C*
ZBIST_2471	-0.967852	3.861896	0.009079	0.037007	*ICL1*
ZBIST_2351	-0.967365	5.627124	0.000116	0.001272	*PDR16*
ZBIST_0834	-0.967044	4.861616	0.000491	0.003957	*ZAP1*
ZBIST_3601	-0.966925	7.850385	3.06E-05	0.00048	*TIM44*
ZBIST_2231	-0.965271	5.33974	0.000378	0.003224	*MRP21*
ZBIST_2728	-0.964521	4.62811	0.001134	0.007502	*RAD53*
ZBIST_1079	-0.964444	4.845004	0.000758	0.00562	*N/A*
ZBIST_0826	-0.964385	5.884074	0.00023	0.002174	*MRPL37*
ZBIST_0318	-0.964216	5.682401	0.000335	0.002943	*ECM16*
ZBIST_1499	-0.963785	10.82055	6.96E-05	0.000844	*RPS15*
ZBIST_2132	-0.962499	5.038912	0.000351	0.003067	*N/A*
ZBIST_4377	-0.961905	5.174845	0.000387	0.003284	*BUD21*
ZBIST_1056	-0.960921	5.673248	0.000407	0.003395	*EMW1*
ZBIST_2504	-0.959612	6.412783	7.29E-05	0.000876	*TOP1*
ZBIST_4603	-0.958965	9.669443	0.000452	0.003693	*TIF11*
ZBIST_1546	-0.958693	6.332294	5.54E-05	0.000719	*RBG1*
ZBIST_1726	-0.954995	9.360239	6.68E-05	0.000831	*SNU13*
ZBIST_1571	-0.95439	4.913613	0.000594	0.004627	*N/A*
ZBIST_4902	-0.953422	8.409935	0.000356	0.003092	*MDH1*
ZBIST_3772	-0.952217	4.039273	0.00312	0.016705	*N/A*
ZBIST_2287	-0.951089	6.016833	8.56E-05	0.000996	*CLN2*
ZBIST_2769	-0.950453	6.094217	0.000129	0.001388	*NOP4*
ZBIST_4276	-0.949872	4.278661	0.002738	0.015106	*CLN3*
ZBIST_4351	-0.949811	5.998089	0.000147	0.001513	*MRC1*
ZBIST_4145	-0.947624	6.888994	0.000169	0.00171	*UTP14*
ZBIST_1129	-0.943415	5.632246	0.000395	0.003317	*UTP13*
ZBIST_0343	-0.941292	4.71253	0.000581	0.004543	*N/A*
ZBIST_4412	-0.934859	10.80966	0.000359	0.0031	*RPS5*
ZBIST_2362	-0.934133	8.277438	0.000152	0.001565	*ADE12*
ZBIST_0969	-0.932856	6.565552	4.93E-05	0.000665	*RPB5*
ZBIST_0702	-0.932707	6.236472	0.000167	0.001699	*NMD3*
ZBIST_0089	-0.932497	7.277141	0.004233	0.020999	*N/A*
ZBIST_3213	-0.932199	9.147907	0.004177	0.020763	*SAH1*
ZBIST_3653	-0.931556	4.993996	0.00121	0.007866	*SNN1*
ZBIST_2522	-0.93122	11.45961	0.000331	0.002913	*RPL24b*
ZBIST_4719	-0.931073	11.97995	0.000506	0.004051	*HHF2*
ZBIST_2417	-0.927428	6.474368	0.001461	0.009198	*MRPL22*
ZBIST_1935	-0.925558	4.055394	0.00507	0.02381	*MME1*

ZBIST_1393	-0.924464	7.94397	6.92E-05	0.000844	*ELO3*
ZBIST_2248	-0.922286	4.885829	0.005409	0.024845	*N/A*
ZBIST_2360	-0.918739	4.189411	0.003589	0.01855	*N/A*
ZBIST_0976	-0.91481	5.918944	0.000994	0.006801	*MSH2*
ZBIST_0807	-0.913934	4.502769	0.005267	0.024366	*ARG2*
ZBIST_3019	-0.906214	3.947822	0.011922	0.045409	*TEM1*
ZBIST_2298	-0.902929	4.954448	0.00777	0.032448	*URM1*
ZBIST_1374	-0.899101	12.0977	0.000873	0.006239	*RPS0a*
ZBIST_1615	-0.898569	11.28834	0.000831	0.006007	*EFT1*
ZBIST_4006	-0.898234	4.091291	0.011191	0.043352	*MDM20*
ZBIST_0554	-0.89576	5.409524	0.00087	0.006229	*LOS1*
ZBIST_0037	-0.895023	7.123254	0.006258	0.027554	*TUF1*
ZBIST_2477	-0.89225	7.228838	0.001221	0.007916	*FCY2*
ZBIST_4629	-0.892181	5.246024	0.001902	0.01141	*GRE2*
ZBIST_4213	-0.890667	4.102543	0.004915	0.023284	*DPH1*
ZBIST_4824	-0.888874	3.925105	0.010422	0.041123	*N/A*
ZBIST_0099	-0.888017	4.794504	0.001367	0.008684	*PCL10*
ZBIST_0389	-0.884296	6.110057	0.001091	0.007306	*RRP8*
ZBIST_4034	-0.882042	4.204608	0.007108	0.03017	*RNH202*
ZBIST_3090	-0.880329	5.336691	0.013236	0.049234	*AFG2*
ZBIST_5017	-0.878728	5.829602	0.001503	0.009412	*MRPL33*
ZBIST_2912	-0.875107	4.38189	0.003974	0.020117	*NCS2*
ZBIST_4248	-0.874597	5.975138	0.009905	0.039619	*GIS2*
ZBIST_1739	-0.874515	8.848656	0.000138	0.001445	*TIM9*
ZBIST_4347	-0.873504	4.209002	0.007904	0.032952	*PRD1*
ZBIST_1894	-0.872468	5.953404	0.000569	0.004476	*N/A*
ZBIST_0384	-0.871453	6.443916	0.000679	0.005133	*RRP1*
ZBIST_3093	-0.871046	9.014703	0.004281	0.02109	*COX8*
ZBIST_3645	-0.869845	4.973979	0.004772	0.022782	*SAL1*
ZBIST_0776	-0.868166	5.071289	0.000761	0.005624	*LSG1*
ZBIST_4319	-0.86371	5.808373	0.000411	0.003406	*N/A*
ZBIST_3654	-0.859754	7.047386	0.00018	0.001804	*N/A*
ZBIST_0212	-0.859161	8.177662	0.000217	0.002066	*QCR9*
ZBIST_2914	-0.858536	8.365809	0.002985	0.016157	*TOM70*
ZBIST_2444	-0.856519	5.03357	0.000915	0.006427	*UTP7*
ZBIST_0338	-0.856508	5.837958	0.000488	0.003945	*CAR2*
ZBIST_3305	-0.854194	7.47505	0.000199	0.001936	*APT1*
ZBIST_4523	-0.854148	4.421406	0.005921	0.026423	*RIX1*
ZBIST_1378	-0.853178	7.546344	0.000212	0.002038	*NHP2*
ZBIST_1423	-0.852429	7.637785	0.002648	0.014819	*FOL2*
ZBIST_2414	-0.852182	8.127356	0.000366	0.003132	*RHO5*
ZBIST_2249	-0.852107	6.743949	0.000271	0.002477	*TOM5*
ZBIST_0084	-0.85118	7.488042	0.000213	0.002042	*NEW1*
ZBIST_1885	-0.850761	4.781854	0.003139	0.016745	*GAS3*
ZBIST_2133	-0.849981	4.241388	0.01066	0.041794	*CTM1*
ZBIST_4975	-0.849091	5.701534	0.000462	0.003759	*MRPL3*
ZBIST_4245	-0.848725	6.049183	0.003789	0.019381	*MRPL17*
ZBIST_0104	-0.847798	5.013948	0.001339	0.008549	*THI6*
ZBIST_4600	-0.846105	5.490419	0.000389	0.003286	*PET111*
ZBIST_4350	-0.845127	5.669181	0.000517	0.004108	*KRR1*

ZBIST_1960	-0.844128	5.678195	0.000658	0.005018	*NOP8*
ZBIST_4482	-0.84328	6.564398	0.000325	0.002865	*NOC2*
ZBIST_2462	-0.841846	6.895185	0.001943	0.011626	*RNR1*
ZBIST_2308	-0.841323	6.945081	0.000829	0.005998	*YJL045W*
ZBIST_4395	-0.839763	4.975723	0.000955	0.006618	*TRZ1*
ZBIST_0344	-0.835332	6.780455	0.000379	0.003228	*SUB2*
ZBIST_2957	-0.835259	4.814603	0.006806	0.029296	*PXL1*
ZBIST_0639	-0.835025	5.509314	0.004484	0.021744	*UTP8*
ZBIST_2581	-0.833999	6.614868	0.000276	0.002506	*N/A*
ZBIST_0428	-0.832732	5.445459	0.001044	0.007049	*MET13*
ZBIST_1662	-0.830799	8.248773	0.000315	0.002806	*MET14*
ZBIST_4887	-0.828578	5.083309	0.006	0.026637	*SXM1*
ZBIST_1210	-0.826188	8.747606	0.001725	0.010538	*RPS22a*
ZBIST_0659	-0.822252	5.346502	0.001949	0.011644	*UTP4*
ZBIST_2296	-0.821363	8.929724	0.000358	0.0031	*TOM40*
ZBIST_1069	-0.821117	7.484614	0.001219	0.007914	*SHH4*
ZBIST_4728	-0.818297	7.858305	0.00064	0.0049	*NOP1*
ZBIST_0619	-0.817203	7.161434	0.000359	0.0031	*NIP1*
ZBIST_4702	-0.816632	5.536166	0.001398	0.008834	*UTP20*
ZBIST_1913	-0.811032	6.62393	0.000413	0.003414	*MRPS28*
ZBIST_0376	-0.80595	6.295772	0.001523	0.009496	*PAB1*
ZBIST_0735	-0.80212	5.038661	0.005198	0.024251	*RRP46*
ZBIST_2412	-0.801853	4.814838	0.00331	0.017436	*IPI3*
ZBIST_1954	-0.800956	4.247622	0.010083	0.040182	*N/A*
ZBIST_4873	-0.800625	5.650006	0.003283	0.01735	*PDR15*
ZBIST_2594	-0.798808	4.386069	0.007493	0.031613	*CHL1*
ZBIST_2415	-0.79813	12.0124	0.000954	0.006618	*RPS3*
ZBIST_1293	-0.796611	8.726308	0.000518	0.004108	*SSZ1*
ZBIST_4238	-0.79488	6.56997	0.000528	0.004181	*YNL247W*
ZBIST_4859	-0.793384	5.315559	0.002332	0.013461	*RRP17*
ZBIST_2361	-0.785144	5.093264	0.003907	0.019877	*POP1*
ZBIST_2163	-0.783251	6.840968	0.000609	0.004712	*DIP2*
ZBIST_1609	-0.782497	8.555746	0.000665	0.00506	*IDH2*
ZBIST_0194	-0.782411	6.007719	0.001767	0.010743	*N/A*
ZBIST_0232	-0.781212	7.178906	0.001145	0.007553	*DRS1*
ZBIST_3383	-0.780817	11.05629	0.000708	0.005306	*HEF3*
ZBIST_4700	-0.779926	7.613859	0.011644	0.044622	*ATP16*
ZBIST_4545	-0.778301	4.876766	0.005772	0.025877	*N/A*
ZBIST_1594	-0.774838	11.13411	0.001542	0.009589	*GDH3*
ZBIST_3153	-0.773381	4.533804	0.006152	0.027142	*MRPL50*
ZBIST_0655	-0.772586	6.77636	0.006281	0.027582	*N/A*
ZBIST_0153	-0.77191	6.821477	0.000763	0.005628	*LYS21*
ZBIST_2507	-0.771356	4.96611	0.002465	0.014075	*N/A*
ZBIST_3347	-0.770818	6.716836	0.000743	0.005524	*CLU1*
ZBIST_0251	-0.770548	5.958034	0.000893	0.006332	*CMS1*
ZBIST_1807	-0.770436	5.002313	0.002244	0.013029	*N/A*
ZBIST_4729	-0.76848	6.495687	0.001165	0.007648	*TSC13*
ZBIST_1471	-0.767475	7.824855	0.000825	0.005989	*MDN1*
ZBIST_0211	-0.766843	6.345422	0.000825	0.005989	*TIM13*
ZBIST_0184	-0.76666	4.832155	0.005044	0.023773	*N/A*

ZBIST_1258	-0.765649	7.032676	0.000808	0.0059	*N/A*
ZBIST_2694	-0.765591	6.428828	0.000809	0.0059	*MCT1*
ZBIST_1141	-0.760803	5.960184	0.000988	0.006776	*TRM8*
ZBIST_4589	-0.759984	4.237143	0.012434	0.046901	*N/A*
ZBIST_2353	-0.759475	4.991696	0.013114	0.048929	*URE2*
ZBIST_4398	-0.75938	5.931675	0.001552	0.009623	*N/A*
ZBIST_0483	-0.758443	6.076425	0.000967	0.006656	*RBL2*
ZBIST_2993	-0.757405	4.831495	0.003532	0.018352	*DUS1*
ZBIST_5013	-0.75567	5.573039	0.010603	0.041669	*AEP2*
ZBIST_2818	-0.755036	7.195532	0.001114	0.007435	*SUI2*
ZBIST_4858	-0.75477	7.052678	0.000965	0.006654	*ERD1*
ZBIST_4171	-0.752287	4.890833	0.007522	0.03168	*YIA6*
ZBIST_0152	-0.751416	5.435556	0.002068	0.012217	*MOT1*
ZBIST_0624	-0.751098	7.467584	0.001049	0.007069	*ZUO1*
ZBIST_0553	-0.750585	5.005669	0.002844	0.015533	*EAP1*
ZBIST_2429	-0.750201	7.559725	0.001191	0.007767	*VCX1*
ZBIST_3662	-0.748255	6.51253	0.004322	0.021169	*N/A*
ZBIST_0183	-0.747375	8.512741	0.001374	0.008719	*N/A*
ZBIST_0405	-0.74509	6.173466	0.001144	0.007553	*NSA1*
ZBIST_2457	-0.742892	7.117002	0.001173	0.00767	*ALD5*
ZBIST_4620	-0.74223	6.877189	0.011141	0.043235	*SCS7*
ZBIST_3109	-0.741729	6.439683	0.001188	0.007758	*IKI3*
ZBIST_1621	-0.740172	5.992961	0.003857	0.019709	*ORT1*
ZBIST_1397	-0.739396	6.866014	0.001256	0.008101	*LHP1*
ZBIST_2093	-0.739347	7.111015	0.001222	0.007916	*ADE3*
ZBIST_0101	-0.733374	7.549126	0.001358	0.008639	*BMS1*
ZBIST_2582	-0.733226	5.11602	0.003113	0.016686	*SMC1*
ZBIST_2695	-0.731931	9.850277	0.001318	0.00845	*ODC2*
ZBIST_0724	-0.730526	5.810688	0.002551	0.014431	*CDC6*
ZBIST_1942	-0.730092	4.708208	0.006345	0.027836	*POL3*
ZBIST_2019	-0.728736	8.30581	0.003141	0.016745	*URA2*
ZBIST_1049	-0.726737	5.826805	0.006644	0.028902	*MNR2*
ZBIST_1698	-0.724717	4.931236	0.004426	0.021544	*MRPL15*
ZBIST_3326	-0.72312	9.379229	0.00148	0.009304	*TIF35*
ZBIST_4787	-0.720758	10.68521	0.003515	0.018284	*YNK1*
ZBIST_4082	-0.720449	5.676448	0.004853	0.023056	*RPA14*
ZBIST_2424	-0.718223	6.197682	0.00203	0.01206	*LYS21*
ZBIST_2448	-0.716047	4.496115	0.011759	0.04496	*N/A*
ZBIST_1303	-0.715493	5.309914	0.003601	0.018591	*RRP4*
ZBIST_0646	-0.714239	7.852422	0.001838	0.011102	*NOP10*
ZBIST_4892	-0.713892	4.917704	0.005275	0.024378	*MDM32*
ZBIST_3948	-0.711498	5.065333	0.004268	0.021052	*CWH41*
ZBIST_4076	-0.711088	5.072043	0.004244	0.021011	*RFM1*
ZBIST_4591	-0.710833	6.70522	0.003126	0.016718	*IDH1*
ZBIST_0225	-0.710451	4.993215	0.005759	0.025843	*SOF1*
ZBIST_0678	-0.707792	5.694464	0.002699	0.014985	*N/A*
ZBIST_3866	-0.706364	8.086416	0.004064	0.020441	*FAU1*
ZBIST_0468	-0.705877	5.474663	0.004444	0.02159	*SLY41*
ZBIST_2941	-0.704473	5.051516	0.005357	0.02471	*N/A*
ZBIST_1974	-0.703887	7.196884	0.005105	0.023921	*CDC33*

ZBIST_3344	-0.703818	7.668312	0.002146	0.012579	*ERG5*
ZBIST_0536	-0.703627	6.485922	0.005525	0.02518	*SUR7*
ZBIST_0810	-0.701532	6.359312	0.01057	0.041574	*MRPL27*
ZBIST_0488	-0.701456	5.035929	0.008991	0.036679	*GCD1*
ZBIST_1796	-0.700989	7.413142	0.002169	0.012684	*URA8*
ZBIST_4165	-0.699932	4.514225	0.013071	0.048805	*FRE3*
ZBIST_0782	-0.698934	7.12523	0.002227	0.01296	*N/A*
ZBIST_0524	-0.698566	7.771569	0.002317	0.013393	*PSD1*
ZBIST_1160	-0.698353	5.98638	0.002463	0.014075	*MRPL9*
ZBIST_2723	-0.697638	5.793023	0.002737	0.015106	*PUS7*
ZBIST_2025	-0.69661	5.713204	0.002899	0.015759	*GCD14*
ZBIST_3547	-0.695517	5.29053	0.007316	0.030944	*MNN5*
ZBIST_3883	-0.694151	4.700663	0.011264	0.043537	*HSP31*
ZBIST_4236	-0.69228	5.908459	0.007017	0.029884	*SUI1*
ZBIST_4576	-0.691808	5.744258	0.003042	0.016412	*ICE2*
ZBIST_4896	-0.690352	5.131088	0.009678	0.038808	*THI80*
ZBIST_1711	-0.690283	7.163537	0.004001	0.020211	*ACO1*
ZBIST_2815	-0.688546	5.134023	0.006358	0.027845	*TMA20*
ZBIST_4022	-0.684825	6.237752	0.008346	0.034501	*ELF1*
ZBIST_0111	-0.684236	6.213524	0.002813	0.015444	*SRP72*
ZBIST_1331	-0.683868	9.571578	0.002629	0.014746	*CAM1*
ZBIST_4720	-0.683727	10.94415	0.004061	0.020441	*HHT2*
ZBIST_0556	-0.68369	6.617392	0.002764	0.01521	*LIA1*
ZBIST_3834	-0.683525	5.169749	0.005411	0.024845	*N/A*
ZBIST_4003	-0.681269	4.910845	0.009532	0.038451	*PAC10*
ZBIST_2144	-0.679583	6.69636	0.004531	0.021925	*ESF1*
ZBIST_3867	-0.677883	9.771268	0.002845	0.015533	*BAT1*
ZBIST_0308	-0.676968	8.484239	0.003217	0.017094	*ADE17*
ZBIST_0323	-0.674551	8.534383	0.007594	0.031916	*COX12*
ZBIST_1618	-0.673611	7.600833	0.003245	0.017202	*ATO3*
ZBIST_0571	-0.670225	10.96083	0.003364	0.017686	*MIR1*
ZBIST_2888	-0.668623	6.457853	0.003391	0.017805	*POL1*
ZBIST_4480	-0.666664	6.281781	0.00446	0.021646	*RET1*
ZBIST_2847	-0.665454	8.60649	0.003694	0.018954	*RPG1*
ZBIST_3670	-0.664849	5.168691	0.006784	0.029296	*TIM12*
ZBIST_4249	-0.664599	6.468224	0.003575	0.018518	*FOL1*
ZBIST_4965	-0.662997	11.41116	0.009636	0.038682	*TMA19*
ZBIST_1767	-0.66161	5.282181	0.006099	0.026974	*N/A*
ZBIST_1209	-0.660449	4.793813	0.011887	0.045307	*VHT1*
ZBIST_2759	-0.660238	5.975085	0.004088	0.020506	*N/A*
ZBIST_4241	-0.659527	6.332272	0.00391	0.019877	*RPA49*
ZBIST_2784	-0.656984	8.285093	0.00426	0.02105	*KRE2*
ZBIST_3807	-0.655099	7.469259	0.004167	0.020754	*ADE2*
ZBIST_0171	-0.653897	8.04922	0.004379	0.021402	*N/A*
ZBIST_1788	-0.653323	6.867671	0.007685	0.0322	*SFC1*
ZBIST_4170	-0.652628	7.453194	0.00431	0.021169	*TOM7*
ZBIST_3378	-0.652065	6.421254	0.00431	0.021169	*DPH5*
ZBIST_1400	-0.65176	7.048797	0.004243	0.021011	*FBP1*
ZBIST_3707	-0.64952	7.09148	0.005745	0.025824	*LYS2*
ZBIST_1162	-0.649037	5.421343	0.006856	0.029451	*IMP3*

ZBIST_4825	-0.647463	8.422795	0.004894	0.023205	*GCN1*
ZBIST_4408	-0.645064	7.492809	0.004831	0.022973	*GCD11*
ZBIST_4222	-0.641339	11.17172	0.004771	0.022782	*MET6*
ZBIST_0700	-0.640726	5.314982	0.007677	0.032197	*DBP8*
ZBIST_3269	-0.639779	7.325606	0.005064	0.023805	*YRM1*
ZBIST_2300	-0.638048	6.083847	0.005385	0.024796	*NUP192*
ZBIST_3133	-0.636663	6.717883	0.005401	0.024845	*RSA4*
ZBIST_1148	-0.636264	9.100441	0.005225	0.024309	*LYS4*
ZBIST_2294	-0.633883	7.619472	0.00563	0.025454	*ERG2*
ZBIST_3074	-0.631024	6.685646	0.00569	0.025627	*UTP21*
ZBIST_1648	-0.62929	8.226446	0.006222	0.02742	*CTP1*
ZBIST_3924	-0.629086	7.185162	0.005847	0.026142	*ERG4*
ZBIST_3715	-0.628271	5.909389	0.011858	0.045231	*MRL1*
ZBIST_2945	-0.627994	6.452405	0.006276	0.027582	*N/A*
ZBIST_1448	-0.627006	6.305058	0.012978	0.048568	*PXR1*
ZBIST_2320	-0.626817	7.960801	0.006277	0.027582	*MGR2*
ZBIST_1588	-0.626498	5.231678	0.009535	0.038451	*NDD1*
ZBIST_1142	-0.626079	6.749919	0.00599	0.026637	*MRPL11*
ZBIST_1228	-0.624291	5.675065	0.007509	0.031653	*N/A*
ZBIST_1291	-0.623225	5.897841	0.007045	0.029978	*SUM1*
ZBIST_5076	-0.622571	5.628668	0.008061	0.033464	*N/A*
ZBIST_1292	-0.620461	6.388291	0.006413	0.028063	*PAN5*
ZBIST_0544	-0.618061	6.917309	0.006807	0.029296	*MNN4*
ZBIST_0868	-0.615473	5.886871	0.007638	0.03206	*RRN3*
ZBIST_2182	-0.614027	5.778504	0.008357	0.034519	*MET1*
ZBIST_4701	-0.613242	5.505193	0.009639	0.038682	*MCD1*
ZBIST_4302	-0.611881	7.307817	0.007427	0.031358	*GIN4*
ZBIST_0370	-0.608307	5.621744	0.009462	0.038284	*MSH6*
ZBIST_2244	-0.603433	6.413494	0.007988	0.033216	*RRP9*
ZBIST_4274	-0.602744	5.284506	0.012049	0.045785	*TOF1*
ZBIST_1646	-0.602032	5.979057	0.00895	0.036571	*TCD2*
ZBIST_1447	-0.601662	11.65824	0.010374	0.040965	*GAS1*
ZBIST_2884	-0.601159	5.41426	0.011396	0.04398	*OCA1*
ZBIST_4885	-0.600586	7.071992	0.0084	0.03464	*UTP5*
ZBIST_2433	-0.59859	6.322754	0.009923	0.039658	*DLD2*
ZBIST_3338	-0.597926	5.998096	0.009449	0.03826	*GIS4*
ZBIST_3873	-0.597768	7.138672	0.010326	0.04084	*N/A*
ZBIST_0348	-0.597694	8.033288	0.009125	0.037161	*TIF34*
ZBIST_2026	-0.586866	6.73869	0.01025	0.040573	*LSM1*
ZBIST_2896	-0.58607	5.838317	0.013046	0.048745	*N/A*
ZBIST_3668	-0.585687	7.160955	0.010347	0.040889	*POL30*

(1) logFC - logarithm base 2 of fold change.
(2) logCPM - logarithm base 2 of counts per million.
(3) FDR - false discovery rate
